# Engineering Robust Supramolecular Nanoassemblies from Amino‐Acid Functionalized Stiff‐Stilbene Amphiphiles

**DOI:** 10.1002/smsc.70305

**Published:** 2026-05-11

**Authors:** Khloe Shuk‐Ying Kwan, Ming‐Hin Chau, Wai‐Ki Wong, Takashi Kajitani, Franco King‐Chi Leung

**Affiliations:** ^1^ Department of Applied Biology and Chemical Technology Research Institute for Future Food The Hong Kong Polytechnic University Hong Kong China; ^2^ Centre for Eye and Vision Research Hong Kong China; ^3^ Open facility Development Office Open Facility Center Institute of Science Tokyo Yokohama Japan

**Keywords:** biocompatible, hydrogel, self‐assembly, stiff‐stilbene amphiphile, structural stability

## Abstract

The unique functional properties and remarkable structural stability of proteins under extreme environments and conditions are largely predetermined by their constituent amino acid groups. However, the specific influence of molecular interactions arising from individual amino acids on overall structural stability of the resulting nanostructures remains insufficiently explored. Herein, we report a newly designed stiff‐stilbene amphiphile functionalized with alanine amino acid groups (**SA**_**Ala**_). The molecular design of **SA**_**Ala**_ incorporates advanced *π–π* interactions between stiff‐stilbene cores and amide hydrogen bonding, which together dictate the intermolecular distance and enable the construction of supramolecular nanostructures with pH and thermal stability at temperature up to 95°C. X‐ray diffraction studies of the macroscopic soft scaffold of **SA**_**Ala**_ further reveal that the presence of higher order tubular packing within the nanotubes of **SA**_**Ala**_ serves as one of the crucial factors in extraordinary stability against structural deformation by external stimulations. Modifications on the steric bulkiness of the amino‐acid side chain from methyl‐groups to isopropyl‐groups can reduce the structural stability of the corresponding nanostructures and macroscopic soft scaffolds stabilities towards light stimulations at macroscopic level. Combined with the demonstrated biocompatibility of **SA**_**Ala**_, these findings pave the way for the next generation design of biocompatible robust supramolecular materials.

## Graphical abstract

This study reports a newly designed stiff‐stilbene amphiphile functionalized with alanine amino acid groups (**SA**_**Ala**_), incorporating advanced *π–π* interactions between stiff‐stilbene cores and amide hydrogen bonding to dictate the intermolecular distance and enable the construction of supramolecular nanostructures with pH and thermal stability, achieved solely through supramolecular interactions without the assist of covalent backbone.

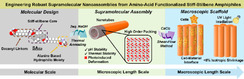

## 1 Introduction

Self‐assembly is a ubiquitous phenomenon of autonomous arrangements of building blocks to form higher order hierarchical structures at multiple length‐scales [1, 2, 3]. Supramolecular assembly involves noncovalent interactions between molecules to create supramolecular nanostructures through minimizing free energy by various forces, in particular, hydrophobic interaction, hydrogen bonds, electrostatic interaction, or van der Waals forces [4, 5, 6, 7, 8, 9, 10]. Inspired by nature, peptides and proteins are exclusive examples of naturally existing sophisticated supramolecular systems, capable of precise formations and folding into various nanostructures by defined sequences [11, 12, 13, 14]. In general, the intrinsic structural stability of proteins are in response to various environmental stimulus including temperature, pH changes, and shear stress for denature of the protein hierarchical structures, yet remarkably structural stable proteins are discovered naturally within thermophilic bacteria, for example, Bacillus stearothermophilus and Thermus aquaticus [15]. Although detailed comparisons between amino acid compositions of enzymes presence in mesophilic bacteria and the analogous ones in thermophilic bacteria cannot be fully explained for the cause of such extraordinary stability, systematic trends are revealed that the abundance of hydrophobic amino acids against its analogous can lead to less thermally stable proteins [16]. Research shows that the origin of extraordinary stability in proteins is thoroughly associated with well‐balanced hydrophobic interactions, hydrogen bonding, and electrostatic interactions [17, 18]. However, the relationship between molecular interactions arises from the individual amino acids on the monomer and overall stability of the resulting nanostructures formed has not yet been fully explored. It inspires us to develop an artificial supramolecular system with superior resistance against external stimulus.

Mimic the features of life‐like materials have drawn strong attention to scientists in developing stimuli‐responsive artificial soft materials under aqueous environments due to their unique and tunable structural‐functional properties [19, 20, 21, 22, 23]. External stimulus, e.g., light, pH, and ion, plays an important role in manipulating the properties of supramolecular soft materials which results in morphological transformations [24, 25, 26], photogating systems [27], and cellular uptake [28, 29, 30, 31, 32, 33, 34]. In the past decade, plenty of examples on stimuli‐responsive soft materials were reported [35, 36, 37, 38], in particular, Feringa's group has studied the assembly structural properties and functionalization of a series of molecular motor amphiphiles [39, 40, 41, 42]. In considering the intrinsic structural‐functional properties of molecular motor amphiphiles in versatile applications, multiple stimuli‐responsive soft materials have been pioneered [43, 44, 45, 46, 47, 48, 49]. First‐generation molecular motor amphiphiles demonstrated superior stimuli‐responsive properties except that the steric effects associated with the presence of methyl groups on the first‐generation molecular motors prohibited the formation of supramolecular structures with large aspect ratio, for example, it is reported that first‐generation molecular motor amphiphiles functionalized with quaternary ammonium cations lead to formation of worm‐like micelles attributed to the nonplanar geometry of the motor‐core [50]. Substitution of the quaternary ammonium cations to carboxylic anion as the hydrophilic motifs of first‐generation molecular motor amphiphiles allows the formation of nanosheets, and morphological transformations were observed upon photoirradiation or altering the pH conditions [51]. The molecular design strategies of the first‐generation molecular motor are yet an ideal hydrophobic core for fabricating a structural stable supramolecular system against external stimulus.

In comparing to the nonplanar geometry of the molecular motor designs, stiff‐stilbene, featured with five‐membered fused rings as a planar aromatic motif, possesses with advanced aromatic *π–π* interaction and high propensity for nanostructures formations with high aspect ratio [52, 53, 54, 55, 56, 57]. We recently demonstrated that stiff‐stilbene amphiphiles (**SAs**) implemented with complementary anionic phosphite and cationic quaternary ammonium groups underwent hierarchical assembly into nanotubes and further coassemble into nanoribbons with greater packing order [58]. **SA** functionalized with histidine amino acid afforded a multicontrolled supramolecular assembling system with good biocompatibility, yet **SA** functionalized with histidine has been limited by photooxidation [59]. We envision that insertions of a hydrophobic amino acid side chain attached on the stiff stilbene core can further strengthen the intermolecular interactions and packing order of the assembled nanostructures, thus improving the overall stability of **SAs** in aqueous medium against external stimulus.

**SCHEME 1:**
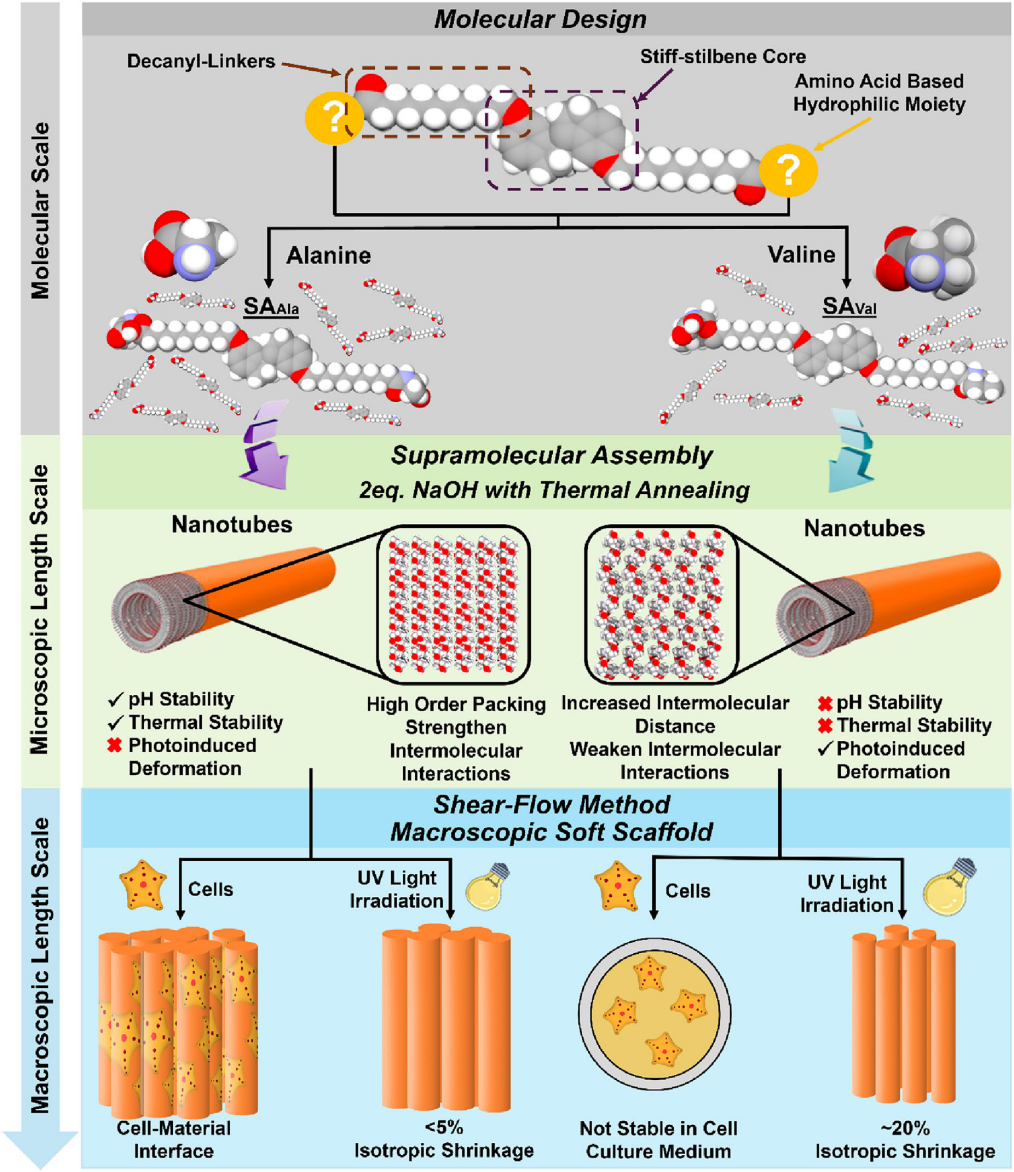
Schematic illustration of stiff‐stilbene amphiphiles (SAs) at multiple length scales.

Herein, we report a new series of biocompatible **SAs** functionalized with various amino acid charged end‐groups, i.e., alanine and valine, where alanine serves with reduced steric bulkiness of the amino acid side chain (methyl‐group) and valine serves as a comparative example with increased steric bulkiness of the amino acid side chain (isopropyl‐group) (Scheme 1). To explore the structural properties of self‐assembled **SAs** with different amino acid side chain attached, structural stability of the nanostructures formed in aqueous medium against various external stimulus including pH environment, temperatures, counterions, and light are extensively investigated. Through meticulous circular dichroism spectroscopy and electron microscopy analyzes, nanotubes formed by **SA** featured with alanine terminal group (**SA**_**Ala**_) demonstrate extraordinary stability in aqueous medium against different external stimulus at microscopic scale. X‐ray diffraction studies of the macroscopic soft scaffold of **SA**_**Ala**_ reveal that higher order tubular packing presence in the nanotubes of **SA**_**Ala**_ serves as one of the crucial factors in extraordinary structural stability against structural deformation by external stimulations. In addition, macroscopic soft scaffold of **SA**_**Ala**_ shows good biocompatibility to function as a cell‐material interfacial platform. By elucidation of the key design of structural stable supramolecular amphiphilic systems, this could open up new prospects towards the development of biocompatible robust supramolecular materials.

## 2 Results and Discussion

### 2.1 Molecular Design and Synthesis

With the structural features of the stiff‐stilbene core, **SAs** were designed and functionalized with different amino acid groups, connected via decanyl‐linkers on both sides of a five‐membered fused ring stiff‐stilbene core to afford a bola‐amphiphilic structural design. This symmetrical stiff‐stilbene design with two alkyl‐chains connected can maximize the planarity of the overall molecular structures, in affording supramolecular nanostructures with large aspect ratio. For the terminal hydrophilic moiety, amino acid group is selected instead of simply carboxylic group due to the fact that **SAs** functionalized by carboxylic groups were commonly shown with low aqueous solubilities. Among all naturally existing *L*‐amino acids, *L*‐alanine is considered one of the less hydrophobic amino acids with its side chain consisting of a single methyl group and *L*‐valine serving as a comparative example of **SA** attached with a more hydrophobic isopropyl group on its side chain. We anticipate that a combination of *π–π* interaction between stiff‐stilbene cores and intermolecular hydrogen bonding between the amide motifs of amino acid terminal group can lead to formation of nanostructures with large aspect ratio, furthermore, tuning the steric bulkiness of the side chain of amino acid terminal group can alter the structural properties of nanostructures formed, thus affecting the overall stability against external stimulus.

**SCHEME 2:**
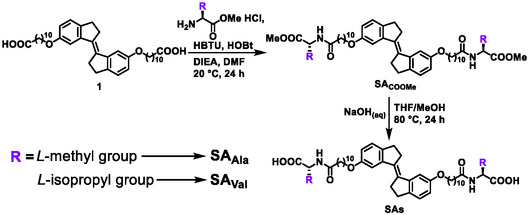
Synthetic Scheme of **SAs**.

*Trans*‐stiff‐stilbene **1** was prepared according to the previously reported procedure. *Trans*‐stiff‐stilbene precursors (**SA**_**COOMe**_) were synthesized by an amide‐bond coupling reaction with different amino acids methyl‐ester, mediated by hexafluorophosphate benzotriazole tetramethyl uranium (HBTU) in dimethylformamide. The obtained precursors (**SA**_**COOMe**_) were subjected to hydrolysis under basic conditions to afford a series of **SAs** (**SA**_**Ala**_ and **SA**_**Val**_) (Scheme 2). The structural characterizations of all newly obtained compounds were summarized in the supporting information, including ^1^H‐, ^13^C‐nuclear magnetic resonance (NMR) and high‐resolution electrospray ionization‐mass spectrometry (ESI‐MS) (Figures S35–S42).

### 2.2 Photoisomerization of SAs

**FIGURE 1:**
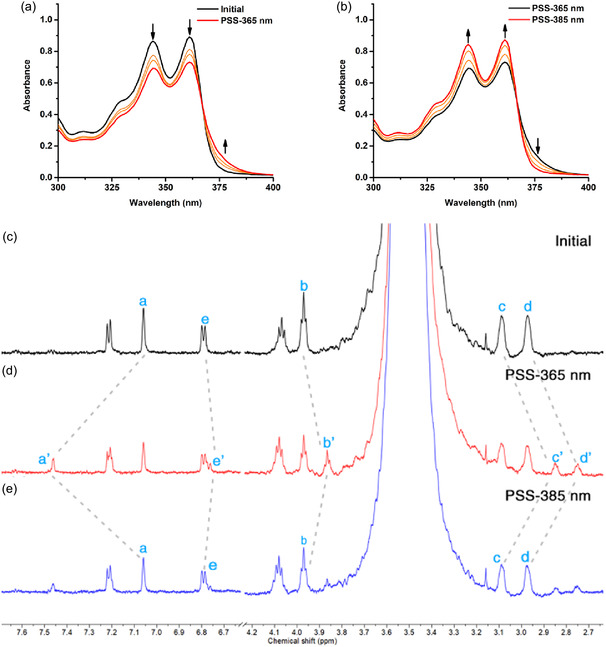
UV–vis absorption spectra of **SA**_**Ala**_ (65 µM), from *trans*‐isomer to *cis*‐isomer and *cis*‐isomer to *trans*‐isomer in DMSO. Absorption spectra of **SA**_**Ala**_ in photostationary state (PSS) (red) (a) after 365 nm irradiation for 1 min (b) after 385 nm irradiation for 2 min. Aromatic and aliphatic region in the ^1^H‐NMR spectra during isomerization processes of **SA**_**Ala**_ (5.5 mM, DMSO‐*d*_6_, 600 MHz), (c) before photoirradiation, (d) irradiated with 365 nm for 1 h at 20°C, (e) followed by 385 nm irradiation for 1 h at 20°C.

A DMSO solution of **SA**_**Ala**_ (65 µM) shows a strong absorption band at 320–370 nm in the UV–vis absorption spectrum at 20°C (Figure 1a). Upon 365 nm photoirradiation, a reduced absorbance was observed at 344 nm with a concomitant spectral shift to obtain a new absorption shoulder peak at 385 nm and a clear isosbestic point appeared at 370 nm, indicating a selective photoisomerization process from *trans*‐**SA**_**Ala**_ to *cis*‐**SA**_**Ala**_. The irradiated solution showed a reverse switching process to *trans*‐**SA**_**Ala**_ after a subsequent irradiation with 385 nm light at 20°C (Figure 1b). The identical photoswitching process of **SA**_**Ala**_ in DMSO‐*d*_6_ solution (5.5 mM) were examined by ^1^H‐NMR studies (Figure 1c and Figure S1). H_a_ (δ = 7.06 ppm) was shifted downfield to δ = 7.46 ppm, while the proton signals of H_b_ (δ = 3.97 ppm), H_c_ (δ = 3.09 ppm), H_d_ (δ = 2.97 ppm), and H_e_ (δ = 6.80 ppm) were shifted upfield to δ = 3.87 ppm, δ = 2.85 ppm, δ = 2.75 ppm and δ = 6.76 ppm, respectively (Figure 1d,e), attaining a *trans*‐**SA**_**Ala**_/*cis*‐**SA**_**Ala**_ isomer ratio of 75:25, upon 365 nm photoirradiation for 1 h at 20°C. The obtained *cis*‐**SA**_**Ala**_ isomer was partially switched back to *trans*‐**SA**_**Ala**_ by photoirradiation of 385 nm for 1 h at 20°C, attaining a *trans*‐**SA**_**Ala**_/*cis*‐**SA**_**Ala**_ isomer ratio of 85:15 (Figure 1e and Figure S1c). A DMSO solution of **SA**_**Val**_ has been studied with UV–vis absorption spectroscopy (Figure S2). **SA**_**Val**_ showed comparable photoswitchabilities to that of observed for **SA**_**Ala**_ (Figure 1a,b). In addition, the photoisomerization processes of DMSO‐*d*_6_ solutions of **SA**_**Val**_ showed similar peak shifting patterns (Figure S3) to that of observed in ^1^H‐NMR spectra of **SA**_**Ala**_ (Figure 1c–e and Figure S1), indicating that changing of amino acid side chain have limited effects on the photoisomerization efficiency of **SAs** in organic medium.

An aqueous solution of **SA**_**Ala**_ shows absorption bands at 320–355 nm and 360–375 nm in the UV–vis spectrum (Figure S4). After 365 nm irradiation at 20°C, the absorption bands at 320–355 nm and 360–375 nm were decreased with a new absorption band appeared at 380–410 nm. No further significant spectral shift was observed upon 385/405 nm irradiation, implying that the reverse switching process of **SA**_**Ala**_ was hindered (Figure S4). With reference to our previous work, a similar phenomenon was observed that the backward photoswitching process of both cationic and anionic modified **SAs** were hindered in aqueous media associated with a highly packed supramolecular structures of *cis*‐**SA**. In this connection, the photoisomerization processes of *cis*‐**SA**_**Ala**_ to *trans*‐**SA**_**Ala**_ should be also hindered due to the formation of highly packed aggregates. In contrast to **SA**_**Ala**_, only decreased absorption bands at 320–355 nm and 360–375 nm was observed over a course of 365 nm irradiation of **SA**_**Val**_ in aqueous media (Figure S5). The decrease in absorption bands upon 365 nm irradiation of **SA**_**Val**_ is likely caused by photodecomposition of stiff‐stilbene core. It indicates that insertions of less bulky amino acid end groups could lead to a partial photoisomerization process instead of entirely decomposed upon 365 nm irradiation of **SA**_**Ala**_, probably associated with the supramolecular structures formed in aqueous media. This further allows for in‐depth investigations into the supramolecular packing of **SAs** attached with different amino acid groups.

### 2.3 Supramolecular Assembly of SAs in Aqueous Media

**FIGURE 2:**
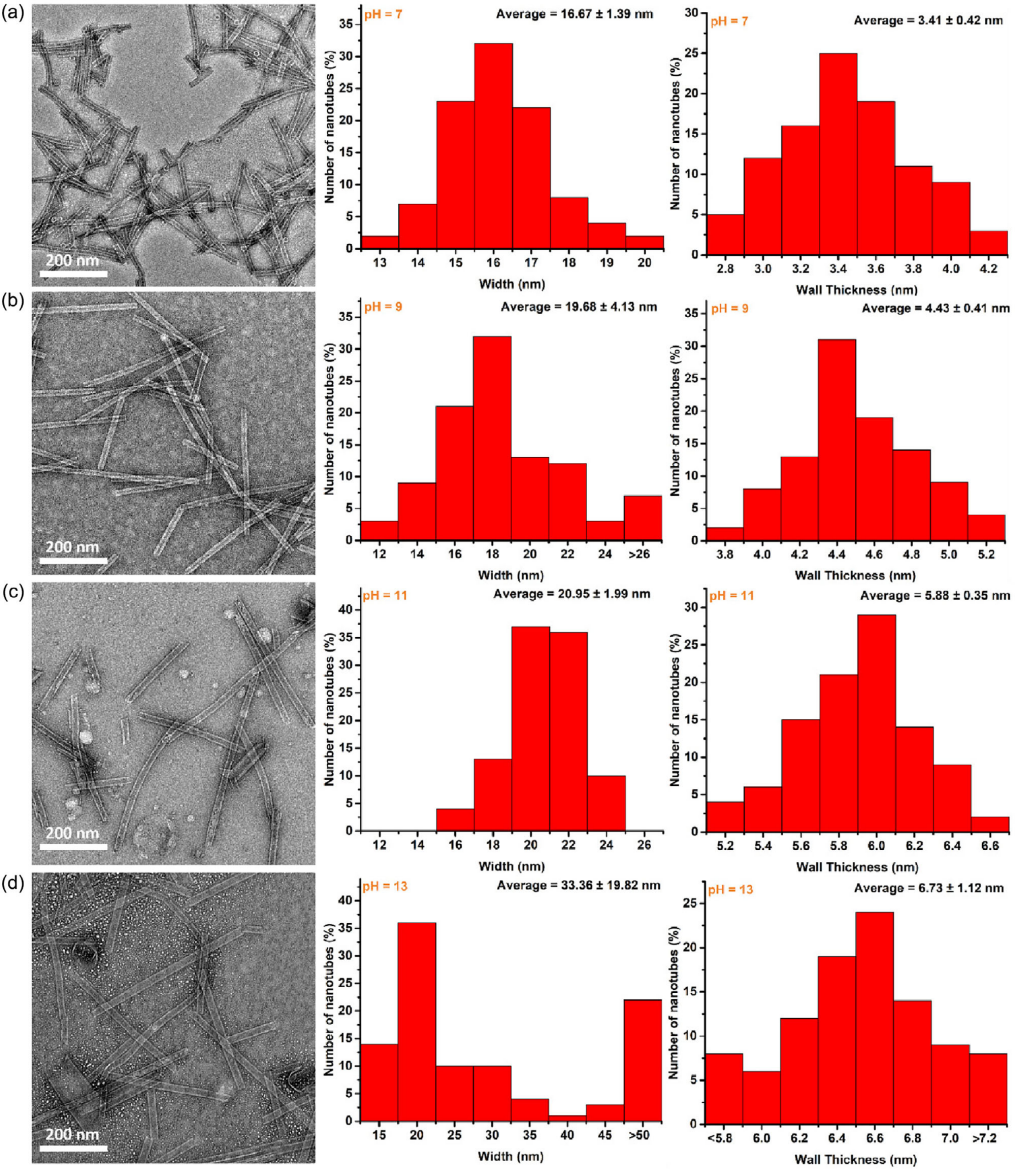
TEM images of thermally annealed solutions of **SA**_**Ala**_ (0.2 wt%, 2.58 mM), along with the corresponding histograms of width and wall thickness distribution of **SA**_**Ala**_ nanotubes formed in water at (a) pH = 7, (b) pH = 9, (c) pH = 11, and (d) pH = 13, respectively.

Amino acid end group of **SA**_**Ala**_ was deprotonated under basic conditions, the influence of pH on self‐assembly can generally be observed by transitions of nanostructures. Critical aggregation concentration (CAC) of **SAs** were estimated by static light scattering (SLS) measurement. The CACs of aqueous solutions of all **SAs** were found below <10 µM, suggesting that **SAs** could undergo supramolecular assembly at low concentration (Figure S6). To examine the pH effect on supramolecular assembly of **SA**_**Ala**_, **SA**_**Ala**_ was initially dispersed in water at different pH (concentration: 0.2 wt%, 2.58 mM); the resulting solution was subsequently annealed at 85°C for 70 min, followed by a cooling process to 20°C at a rate of 1°C/min. TEM images of the annealed solutions showed that nanotubes of **SA**_**Ala**_ were observed at different pH conditions (pH = 7, pH = 9, pH = 11, and pH = 13) (Figure 2). Over hundreds of nanotubes were analyzed with a range of width and length under different pH conditions. At neutral conditions (pH = 7), nanotubes formed by **SA**_**Ala**_ were measured as 16.7 ± 1.4 nm in width, 3.41  ± 0.42 nm in wall thickness and 207.8 ± 21.6 nm in length, respectively (Figure 2a and Figure S7), suggesting tight assembly packing of **SA**_**Ala**_ originated from the advanced *π–π* interaction between stiff‐stilbene cores with amide hydrogen bonds. Under basic condition (pH = 9), consistently, nanotubes of **SA**_**Ala**_ were observed with an increased width (19.7 ± 4.1 nm), wall thickness (4.43 ± 0.41 nm) and length (345.55 ± 142.12 nm) (Figure 2b and Figure S8). A solution of **SA**_**Ala**_ (pH = 11) was imaged and found that the width and length of nanotubes (width: 21.0 ± 2.0 nm, wall thickness: 5.88 ± 0.35 nm and length: 331.0 ± 112.8 nm) were similar to the nanotubes formed in the solution of **SA**_**Ala**_ at pH 9 (Figure 2c and Figure S9), identifying fully deprotonation of **SA**_**Ala**_ enabled with slight increase in width and length of nanotubes. Further basified the solution of **SA**_**Ala**_ (pH = 13) showed that a small portion of nanotubes were significantly widened (width: 33.4 ± 19.8 nm and wall thickness: 6.73 ± 1.12 nm) along with the length of nanotubes of **SA**_**Ala**_ increased to 400.2 ± 190.0 nm (Figure 2d and Figure S10), the significant transitions of the structural properties of **SA**_**Ala**_ nanotubes suggesting additional parameters should be considered. Given that sodium hydroxide was applied for tuning pH of aqueous solutions of **SA**_**Ala**_, the corresponding sodium ions (Na^+^) concentration in the resulting solution was increased along with the elevating pH. Consequently, increased ionic‐strength of the **SA**_**Ala**_ solution (pH 13) possibly shielded electrostatic repulsions between the negatively charged carboxylic groups of **SA**_**Ala**_, and promote tighter packing of the supramolecularly assembled **SA**_**Ala**_, leading to increased width and length of nanotubes of **SA**_**Ala**_.

To further verify the influence of the ion‐strength on the structural properties of the supramolecular assembly of **SA**_**Ala**_, different counterions (Na^+^ and Ca^2+^) to carboxylate‐groups of **SA**_**Ala**_ were introduced into the solution of nanotubes of **SA**_**Ala**_ at pH 9, respectively. Upon addition of two equivalents of Na^+^ into the aqueous solution of **SA**_**Ala**_ at pH = 9, the solution was imaged with TEM to reveal supramolecular nanotubes with similar width and length (Figure S12a) to that observed in the untreated solution (Figure 2b). The electron microscopic results are consistent with the increased width and length of nanotubes of **SA**_**Ala**_ at pH = 9 and at pH = 11. Furthermore, the ratio of Na^+^ added into the aqueous solution of **SA**_**Ala**_ at pH = 9 was increased to four equivalents, resultingly, a couple of stacked nanotubes of **SA**_**Ala**_ were observed (Figure S12b), suggesting that an increased ion‐strength promotes further aggregations of nanotubes. Two equivalents of Ca^2+^ were introduced to the solution of **SA**_**Ala**_ at pH = 9, consistently, to afford strong aggregations of nanotubes (Figure S12c). This result suggested that the increased ionic‐strength can lead to advanced aggregations of the nanostructures associated with a reduced electrostatic repulsion between the negatively charged carboxylic groups of **SA**_**Ala**_.

In considering the intrinsic structural helicity of the **SA**_**Ala**_ nanotubes, CD measurements were employed to monitor aqueous solutions of **SA**_**Ala**_ (50 µM) at different pH (pH = 7, pH = 9, pH = 11, and pH = 13). All CD spectra of the solutions showed a positive Cotton effect at 325–375 nm with comparable CD intensity (Figure S13). It is noted that the CD signals for aqueous solutions of **SA**_**Ala**_ (50 µM) at pH = 7, pH = 9 and pH = 11 were similar with a slightly reduced CD signals than that observed in the aqueous solution of **SA**_**Ala**_ at pH 13, suggesting good structural stabilities of **SA**_**Ala**_ nanotubes at high pH. To further investigate the structural stability of nanostructures at high pH, aqueous solutions of **SA**_**Ala**_ at pH 11 and pH 13 were subjected to an aging process for 1 month at room temperature. Nanotubes of **SA**_**Ala**_ were revealed with similar width and length to that observed in the freshly prepared solutions without any sign of degradation over aging processes at high pH (Figure S14), further verifying good structural stability of the **SA**_**Ala**_ nanotubes at high pH.

The structural influence of amino acid side chains on the resulting supramolecular assembled structures of **SAs** was investigated. An aqueous solution of **SA**_**Val**_, featured with increased steric bulkiness amino acid side chain, i.e., isopropyl‐group, was imaged by TEM under different pH conditions. Nanotubes were observed in the solutions of **SA**_**Val**_ at neutral (pH = 7) and slightly basic condition (pH = 9), as imaged by TEM (Figure S15a,b). However, no ordered nanostructures were observed in the solution of **SA**_**Val**_ at higher pH condition (pH ≥ 11) (Figure S15c,d), suggesting that increased steric bulkiness of the amino acid side chain destabilized the nanostructures at high pH conditions even in the presence of the advanced *π–π* interaction between stiff‐stilbene cores with amide hydrogen bonds. The impacts of the increased size of amino acid side chain on assembled nanostructures of **SAs** at different pH were illustrated. Both **SA**_**Ala**_ and **SA**_**Val**_ were able to form nanotubes at neutral conditions, in particular, **SA**_**Ala**_ was able to form nanotubes at extreme basic conditions (pH = 11 and pH = 13). In contrast, **SA**_**Val**_ can only assemble into nanotubes from pH = 7 to pH = 9, transforming to disordered nanoaggregates at pH = 11. Given that the steric hindrance of the alanine group of **SA**_**Ala**_ (~11.5) is significantly lower than valine group of **SA**_**Val**_ (~19.8), both fully deprotonated **SA**_**Ala**_ and **SA**_**Val**_ (above pH 9) should be assembled with the advanced *π–π* interaction between stiff‐stilbene cores with amide hydrogen bonds to determine the intermolecular distance. The bulkier valine group of **SA**_**Val**_ can increase intermolecular distance in providing balanced supramolecular nanostructures. Resultingly, aqueous solutions of **SA**_**Val**_ showed loss of nanotubular structures upon pH increased over 11 (Figure S15c,d), also, the observed positive Cotton effect at 325–360 nm in CD spectra of **SA**_**Val**_ was diminished at pH = 11 (Figure S16). The spectroscopic and electron microscopic results further confirmed that the advanced nanostructural stability can be finely adjusted with simply increase of steric bulkiness of the amino acid side chain.

**FIGURE 3:**
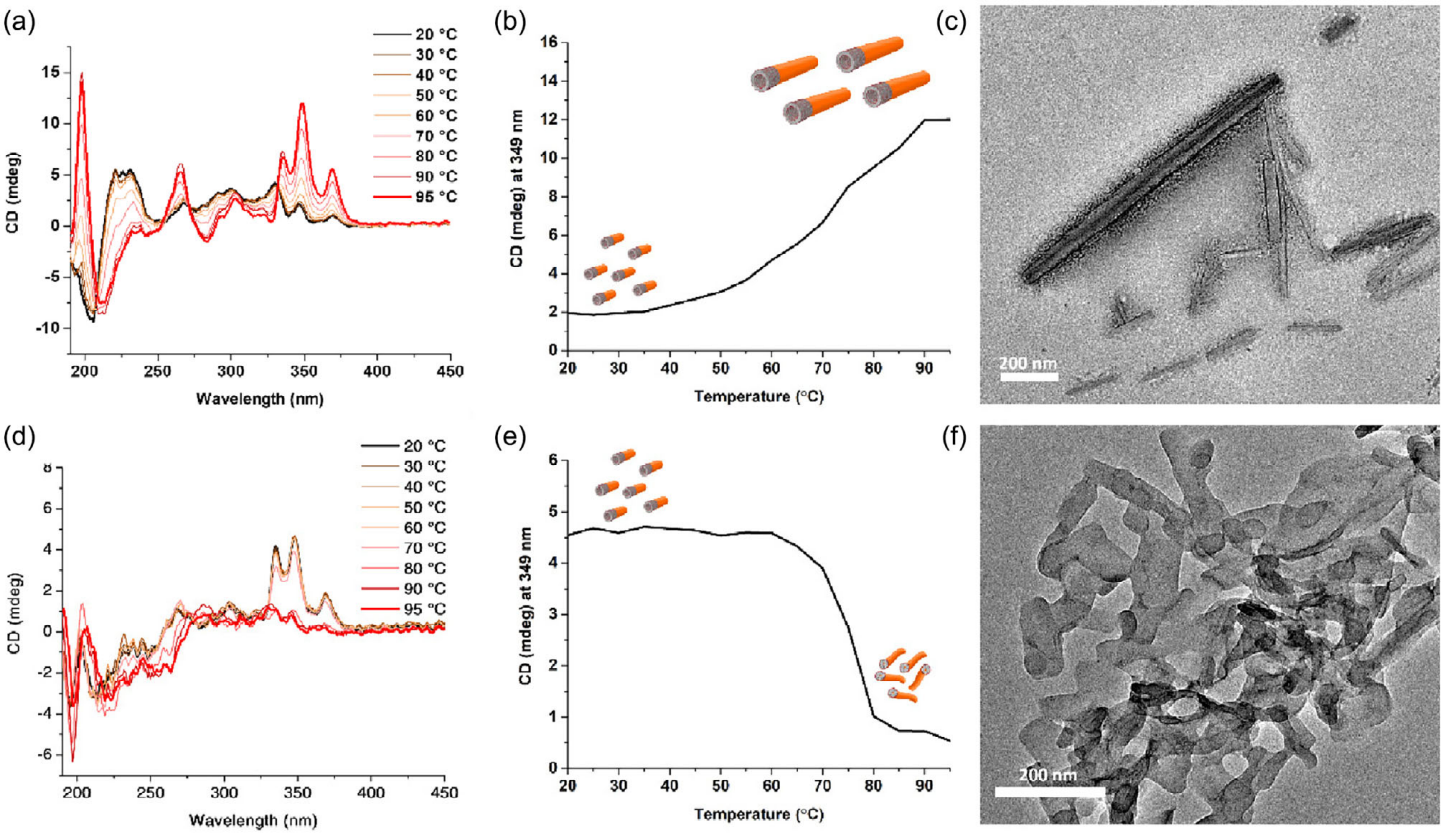
VT‐CD spectra of (a) **SA**_**Ala**_ and (c) **SA**_**Val**_ in water (50 µM, pH = 9) from 20°C to 95°C and the corresponding change in CD signals at 349 nm for (b) **SA**_**Ala**_ and (e) **SA**_**Val**_ upon elevating temperature from 20°C to 95°C at a rate of 1°C/min. TEM images of the resulting solutions of (c) **SA**_**Ala**_ and (f) **SA**_**Val**_ after thermal annealing.

Apart from the demonstrated excellent structural stability of **SA**_**Ala**_ nanotubes under various pH environments, Variable Temperature‐Circular Dichroism (VT‐CD) spectroscopy was also employed to examine the thermal stability of **SA**_**Ala**_ under aqueous media. An aqueous solution of **SA**_**Ala**_ (50 μM, pH = 9) was thermally annealed to 95°C at a heating rate of 1°C/min and stabilized at 95°C for 10 min. This thermal annealing process was monitored by VT‐CD spectroscopy, showing a gradual increase in CD signal at 325–375 nm for **SA**_**Ala**_ along with elevating temperatures (Figure 3a). The gradually increased CD signal at 190–210 nm is associated with n→π* transitions of the amide carbonyl‐group, suggesting a strengthened supramolecular helicity and structural orderliness within the **SA**_**Ala**_ nanotubes upon heating, possibly originated from increased hydrophobic interaction at close proximity between the alkyl chain linked with alanine amino acid group of **SA**_**Ala**_. Together with the increase in CD signal at around 325–375 nm upon thermal annealing, it suggested that short nanotubes of **SA**_**Ala**_ (Figure S17) were gradually elongated to longer nanotubes with the assist of hydrophobic interaction between the alkyl chains and advanced *π–π* interaction between stiff‐stilbene cores (Figure 3b). Furthermore, the resulting solution of annealed **SA**_**Ala**_ was sequentially subjected to a cooling process from 95°C to 40°C at a cooling rate of 1°C/min and the CD signals remained stable over the declining temperature (Figure S18). TEM images of the annealed solution of **SA**_**Ala**_ at 95°C were observed with similar nanotubular structures (Figure 3c), suggesting a good thermal stability of nanotubes formed by **SA**_**Ala**_ with a structural melting temperature over 95°C.

An aqueous solution of **SA**_**Val**_ (50 μM, pH = 9), followed by the identical annealing method, showed a slight decrease in CD signal upon increasing temperature from 20°C to 60°C and a significant drop in CD signal at 325–375 nm after the temperature increased over 80°C (Figure 3d). Comparing to the VT‐CD results of **SA**_**Ala**_, no obvious CD signal is observed at the region of 190–210 nm, suggesting limited intermolecular hydrogen bonding within nanotubes of **SA**_**Val**_ upon thermal annealing. The dramatic fall of CD signal of **SA**_**Val**_ originated from morphological supramolecular transformation of nanotubes of **SA**_**Val**_ upon heating, which was supported by the TEM images of the annealed solution of **SA**_**Val**_ at 95°C with disordered aggregates (Figure 3f). The results suggested that a significantly reduced thermal stability of **SA**_**Val**_ with a structural melting temperature below 80°C. It is clearly shown that advanced nanostructural thermal stability can be adjusted with simply increase of steric bulkiness of the amino acid side chain. More importantly, the molecular design of **SA**_**Ala**_, featured with advanced *π–π* interaction between stiff‐stilbene cores with amide hydrogen bonds to determine the intermolecular distance, can afford supramolecular nanostructures with thermal stability at 95°C with solely supramolecular interactions without any covalent backbone. The thermal stability of the helical nanotubular structures of **SA**_**Ala**_ provides the structural melting temperature even higher than typical DNA melting temperature into single strands [60, 61].

**FIGURE 4:**
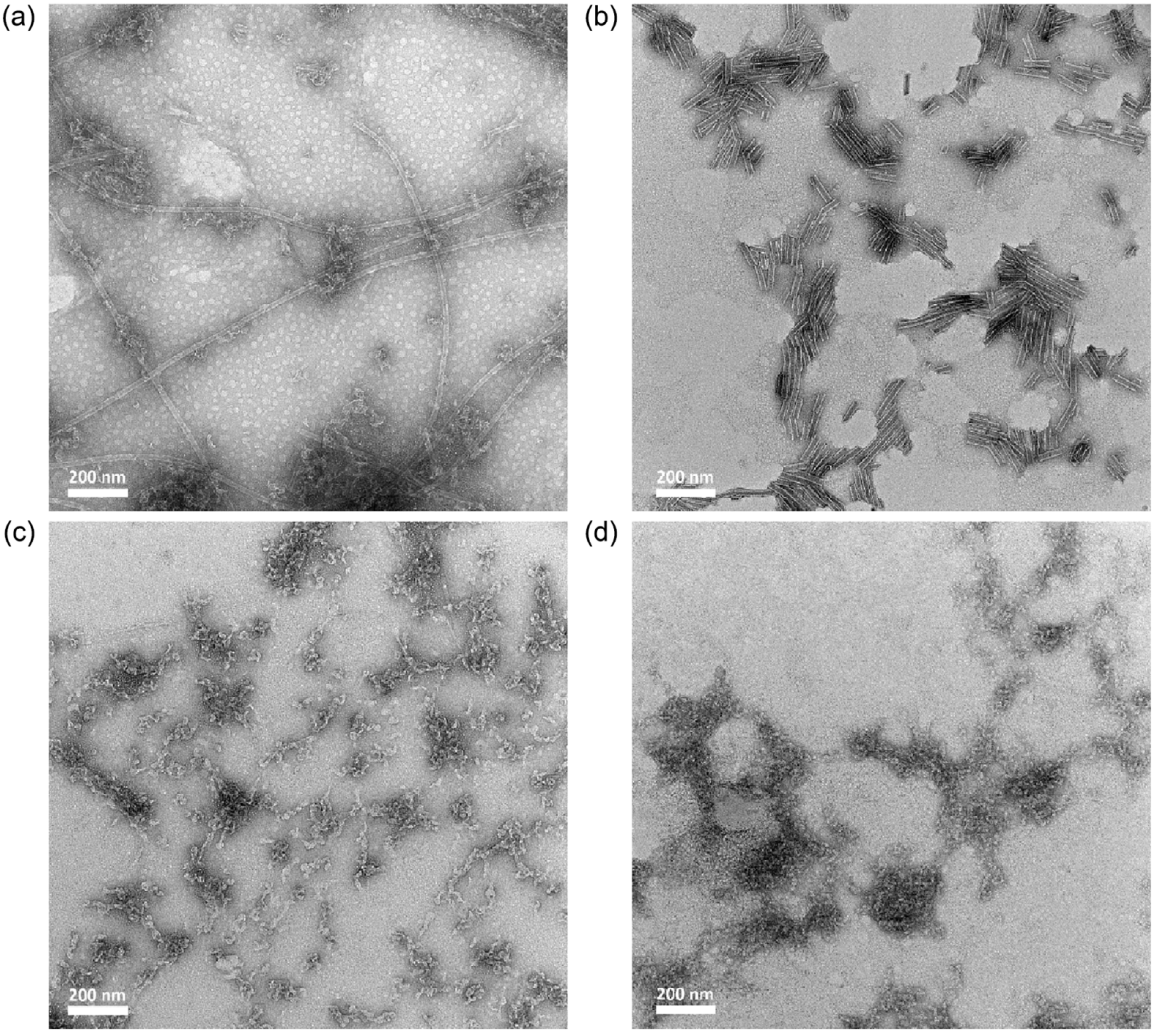
TEM images of thermally annealed solutions of **SA**_**Ala**_ (0.2 wt%, 2.58 mM) mixed with (a) 20% hexafluoroisopropanol and (b) 20% ethanol. TEM images of thermally annealed solutions of **SA**_**Val**_ (0.2 wt%, 2.41 mM) mixed with (c) 20% hexafluoroisopropanol and (d) 20% ethanol.

Considering that the significant differences are observed in the formation of nanoassemblies by **SA**_**Ala**_ and **SA**_**Val**_ under various environmental conditions, it is of particular interest to study the effects of specific interactions on supramolecular assemblies of **SA**_**Ala**_ and **SA**_**Val**_. In this connection, hexafluoroisopropanol is used to selectively hinder hydrogen bonds formation and ethanol is used to minimize the hydrophobic interactions [62, 63, 64]. 20% of hexafluoroisopropanol or 20% of ethanol were introduced to the thermally annealed solutions of **SA**_**Ala**_ and **SA**_**Val**_. The obtained solutions were subsequently annealed at 85°C for 10 min and followed by a cooling process to 20°C at a rate of 1°C/min. TEM images of the annealed solution of **SA**_**Ala**_ mixed with 20% of hexafluoroisopropanol show a mixture of long nanotubes and irregular aggregates (Figure 4a). The elongation of **SA**_**Ala**_ nanotubes is consistent with the spectral change observed in the VT‐CD spectra of **SA**_**Ala**_ upon elevating temperatures, further suggesting that suppressed formations of intermolecular hydrogen bonds lead to elongation of the **SA**_**Ala**_ nanotubes. Furthermore, TEM images of the annealed solution of **SA**_**Ala**_ mixed with 20% of ethanol showed shorter nanotubes (Figure 4b) than that of the nanotubes observed in the presence of hexafluoroisopropanol. The nanostructure results suggested that hydrophobic interaction plays a critical role in both formation and elongation of the nanotubes formed by **SA**_**Ala.**_

In contrast, both TEM images of the annealed solution of **SA**_**Val**_ mixed with either 20% of hexafluoroisopropanol (Figure 4c) or 20% of ethanol (Figure 4d) show irregular aggregates only, indicating the necessities of both hydrophobic interaction and hydrogen bonding for the formation of **SA**_**Val**_ nanotubes. The obtained results suggested that the intrinsic hydrophobicity and steric bulkiness of the amino acid side groups are the crucial factors to determine the intermolecular distance and structural orderliness of the resulting supramolecular nanostructures, thus the bulkier valine group of **SA**_**Val**_ with an increased intermolecular distance requires additional interaction to induce closer proximity intermolecularly for formation of well‐organized supramolecular nanostructures.

### 2.4 Supramolecular Transformations of SAs Upon Photoirradiation

**FIGURE 5:**
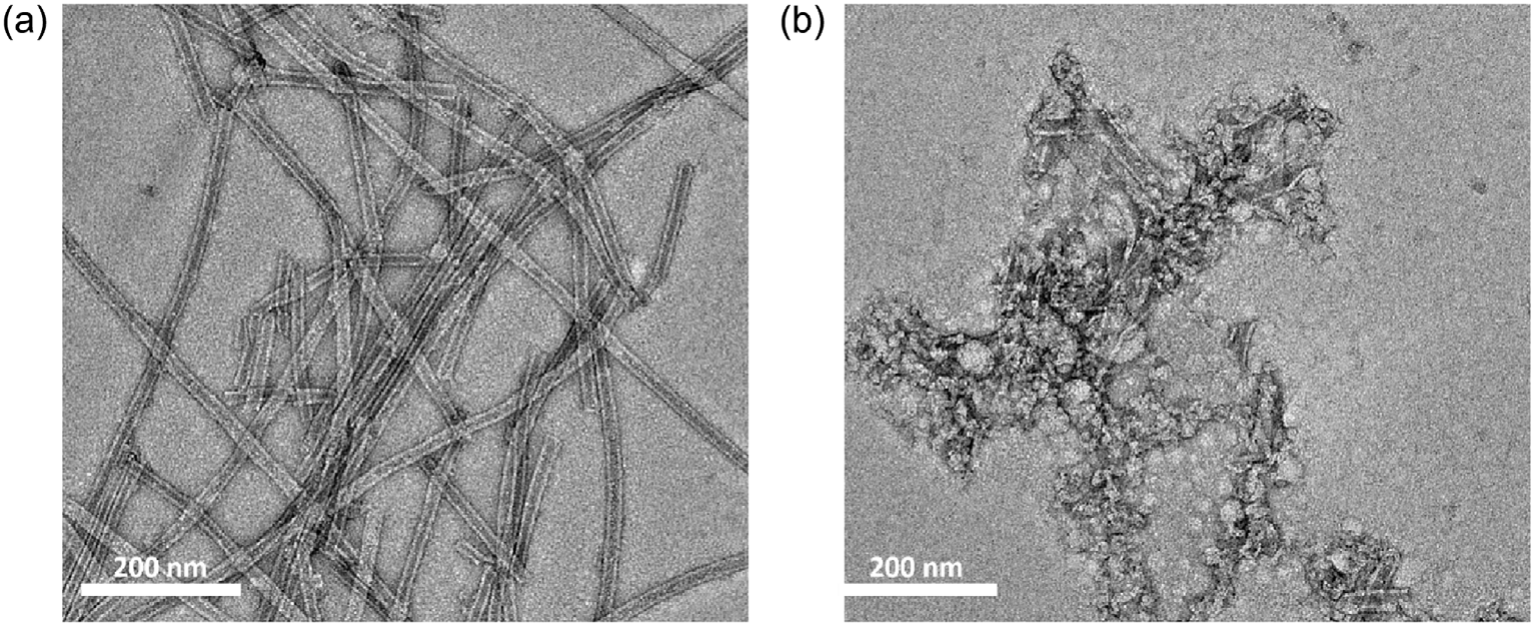
TEM images of (a) **SA**_**Ala**_ (0.2 wt%, 2.58 mM, pH = 9) and (b) **SA**_**Val**_ (0.2 wt%, 2.41 mM, pH = 9) in water after 365 nm photoirradiation for 1 h.

Supramolecular transformations of **SA**_**Ala**_ in aqueous media driven by 365 nm light irradiation was imaged by TEM. TEM image showed the nanotubes of **SA**_**Ala**_ at pH = 9 nearly remained unchanged after the 365 nm UV‐light irradiation for 1 h (Figure 5a, Figure S19), indicating that the structural stability of the nanotubular structures of **SA**_**Ala**_ hinders the morphological transformation driven by photoirradiation. This suppressed supramolecular transformation of **SA**_**Ala**_ should be associated with tightly packed nanotubes to hinder photoisomerization of the stiff‐stilbene core. The results can be further supported by the observed limited spectral changes in UV–vis spectra of **SA**_**Ala**_ over photoirradiation. An aqueous solution of **SA**_**Val**_ at pH = 9 showed nanotubes morphological transformations into irregular structures (Figure 5b, Figure S20) upon photoirradiation. The obvious morphological transformation difference between the nanotubular structures of **SA**_**Ala**_ and **SA**_**Val**_ should also be originated from the intrinsic steric bulkiness difference of the amino acid side chain of **SA**_**Ala**_ and **SA**_**Val**_ in the predefined intermolecular distance by the *π–π* interaction between stiff‐stilbene cores and amide hydrogen bonds. The bulkier valine group of **SA**_**Val**_ can increase intermolecular distance by reducing the tightly packed nanotubular structure to enable photoisomerization of the stiff‐stilbene core. Resultingly, the increased intermolecular distance within the nanotubes of **SA**_**Val**_ reduces structural stability of nanotubes of **SA**_**Val**_ in aqueous medium. To validate the structural stability of the nanotubes of **SA**_**Ala**_ in aqueous media, the DMSO solution of **SA**_**Ala**_, with confirmed photoswitchability and photoisomerization processes, was imaged by TEM to investigate the possibility of photo‐triggered supramolecular assembly transformations. Nanotubular structures of the DMSO solution of **SA**_**Ala**_ were observed (0.2 wt%, 2.58 mM) (Figure S21a). Upon photoirradiation to a degassed DMSO solution of *trans*‐**SA**_**Ala**_ with 365 nm light for an hour, partial supramolecular transformations from nanotubes into nanoribbon were observed as a result of *trans*‐**SA**_**Ala**_ to *cis*‐**SA**_**Ala**_ photoisomerization (Figure S21b). Similar supramolecular transformations were observed in the DMSO solution of **SA**_**Val**_ that **SA**_**Val**_ initially assembled into nanoribbon‐like structures and subsequently transformed into nanotubular structures upon 365 nm light irradiation (Figure S22). The results indicated that photoirradiation of **SA**_**Ala**_ can induce morphological transformations in organic medium instead of aqueous medium, suggesting that the supramolecular interactions can be significantly reduced in the presence of organic solvent. The obtained results of the structural stability and properties of **SAs** with different amino acid side chains enable further studies at the macroscopic length‐scale.

### 2.5 Structural Properties of Supramolecular Macroscopic Soft Scaffolds of SAs

**FIGURE 6:**
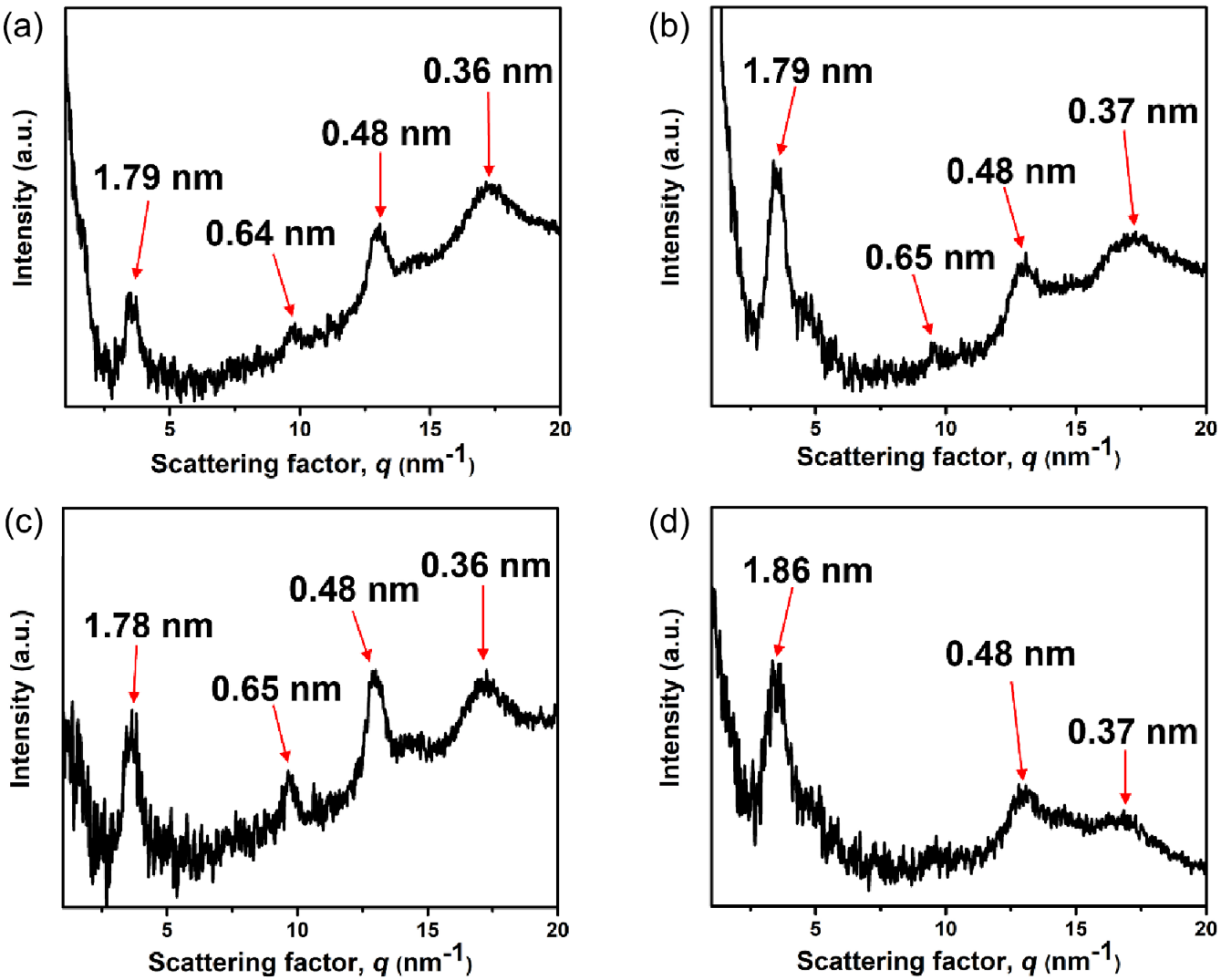
1D‐WAXD pattern of a macroscopic soft scaffold of **SA**_**Ala**_ (a) before and (c) after 365 nm UV light irradiation for 1 h. 1D‐WAXD pattern of a macroscopic soft scaffold of **SA**_**Val**_ (b) before and (d) after 365 nm UV light irradiation for 1 h.

With the demonstrated high structural stability of **SA**_**Ala**_ towards the thermal and pH stimulations, an off‐white macroscopic soft scaffold of **SA**_**Ala**_ (5.0 wt%, annealed at 85°C for 10 min) was prepared by shear‐flow method in a shallow pool of solution of CaCl_2_ (150 mM) (Figure S23a). To avoid prolonged thermal annealing (70 min) to induce sample drying with high sample concentration of **SA**_**Ala**_ (5.0 wt%, 65 mM), the shorter thermal annealing duration was applied. The macroscopic soft scaffold of **SA**_**Ala**_ was observed with rough surface and limited alignment by scanning electron microscopy (SEM) (Figure S23b). The macroscopic soft scaffold of **SA**_**Ala**_ with ~400 µm in diameter was revealed under optical microscopy (OM); however, no significant birefringence was observed with crossed polarizers (Figure S23c). Through‐view wide‐angle X‐ray diffraction (WAXD) was applied to investigate structural parameters and orientation of *trans*‐**SA**_**Ala**_ nanotubes in the macroscopic soft scaffold. The 2D‐WAXD image of a macroscopic soft scaffold of **SA**_**Ala**_ with 20 min X‐ray exposure time was observed without any sign of directional diffraction (Figure S24a). The 1D WAXD profile of the **SA**_**Ala**_ scaffold shows a diffraction peak with *d*‐spacing of 1.79 nm, possibly originating from the stacking of half of **SA**_**Ala**_ molecule within the nanotubes with respect to the symmetrical molecular design of **SAs** (Figure 6a). A diffraction peak located at *d*‐spacing of 0.64 nm corresponds to the shorter molecular axis of the **SA**_**Ala**_, suggesting a higher order tubular packing of the nanotubes formed by **SA**_**Ala**_, while the peak located at *d*‐spacings of 0.48 nm and 0.36 nm should be originated from the stacking of stiff‐stilbene moieties associated with the intermolecular hydrogen bonds between amide motifs of alanine and *π–π* packing of the stiff‐stilbene cores, respectively (Figure S24c) [59]. The results suggested that the supramolecular nanotubes of **SA**_**Ala**_ remained their structural integrity at the macroscopic length‐scale. Furthermore, the 2D‐WAXD image of a macroscopic soft scaffold of **SA**_**Ala**_ at pH = 11 with 20 min X‐ray exposure time was observed without any sign of directional diffraction (Figure S25a). The 1D WAXD profile of the **SA**_**Ala**_ scaffold at pH = 11 shows a diffraction peak shift with *d*‐spacings from 1.79 to 1.87 nm, possibly originating from the tilted stacking of **SA**_**Ala**_ molecule within the nanotubes with respect to the increased electrostatic interaction (Figure S25b).

Although **SA**_**Val**_ consists of a bulkier amino acid side chain than **SA**_**Ala,**_ **SA**_**Val**_ also formed a macroscopic soft scaffold without structural alignment and birefringence (Figures S26). Similarly, the 2D‐WAXD image of the macroscopic soft scaffold of **SA**_**Val**_ was observed without a sign of directional diffraction (Figure S27a). In contrast to the macroscopic soft scaffold of **SA**_**Ala,**_ the 1D WAXD pattern of macroscopic soft scaffold of **SA**_**Val**_ shows a diffraction peak with *d*‐spacing of 1.79 nm, originating from similar molecular length observed in the 1D WAXD pattern of **SA**_**Ala**_. However, the higher order tubular packing has almost vanished with a weak peak observed at *d*‐spacing of 0.65 nm, suggesting that the steric bulky valine group disrupts the supramolecular packing structure of **SA**_**Val**_ by increasing the intermolecular distance controlled by the *π–π* interaction between stiff‐stilbene cores and amide hydrogen bonds (Figure 6b). The diffraction peak located at *d*‐spacings of 0.48 and 0.37 nm indicated the presence of intermolecular hydrogen bonds between amide motifs of valine and *π–π* interaction between stiff stilbene cores, respectively. The WAXD results of both macroscopic soft scaffolds of **SA**_**Ala**_ and **SA**_**Val**_ were suggesting that the predefined intermolecular distance by *π–π* interaction between stiff‐stilbene cores and amide hydrogen bonds can be adjusted even the nanostructures stabilized at the macroscopic length‐scale. In considering the results observed from microscopic to macroscopic length‐scales, we anticipate that the higher order nanotubular packing of **SA**_**Ala**_ enabling advanced structural stability towards external stimulations at macroscopic length‐scale. A macroscopic soft scaffold of **SA**_**Ala**_ was subjected to WAXD study after 365 nm UV‐light irradiation for 1 h. The obtained 2D‐WAXD image shows no sign of directional alignment (Figure S24b) and the converted 1D‐WAXD profile shows a minimal diffraction peak shift with *d*‐spacings from 1.79 and 0.64 nm (Figure 6a) to 1.78 and 0.65 nm (Figure 6c), suggesting no significant disruption of the tightly packed structure of **SA**_**Ala**_ upon 365 nm UV light irradiation. In contrast, a macroscopic soft scaffold of **SA**_**Val**_ was subjected to WAXD study after 365 nm UV‐light irradiation for 1 h. 2D‐WAXD image showed no sign of directional alignment (Figure S27b), while the converted 1D‐WAXD profile shows significant diffraction peak shift with *d*‐spacings from 1.79 nm (Figure 5b) to 1.86 nm (Figure 6d), along with a diminished diffraction peak with *d*‐spacings of 0.65 nm. These significant changes in 1D‐WAXD pattern of macroscopic soft scaffold of **SA**_**Val**_ after 365 nm UV light irradiation are possibly originating from reduction of its structural planarity by photodecomposition of **SA**_**Val.**_ In addition, a significantly reduced signal for the diffraction peak with *d*‐spacings of 0.37 nm indicated the weakening of *π–π* interaction between stiff stilbene cores upon photoirradiation. The observed results further verified that in the presence of higher order nanotubular packing in the macroscopic soft scaffold of **SA**_**Ala**_ can maintain structural stability across multiple length‐scales due to its advanced supramolecular interactions. Besides, structural stability can be finely adjusted by the increase of steric bulkiness of the amino‐acid side chain from methyl‐group to isopropyl‐group, in reducing the resulting nanostructures and macroscopic soft scaffolds stabilities towards light stimulations.

Although nanotubes of **SA**_**Ala**_ demonstrated good stability against pH, temperature and photoirradiation at microscopic level, it remains difficult to investigate the thermal stability of **SA**_**Ala**_ at macroscopic level because of high water content of the macroscopic soft scaffold of **SA**_**Ala**_ (95% of water). With the observed difference between the macroscopic soft scaffolds of **SA**_**Ala**_ and **SA**_**Val**_ upon 365 nm photoirradiation in WAXD studies, it is of particular interest to examine the potential structural change of the macroscopic soft scaffold of **SA**_**Ala**_ and **SA**_**Val**_ over the course of 365 nm photoirradiation. A macroscopic soft scaffold of **SA**_**Ala**_ prepared in CaCl_2_ solution was monitored and further irradiated with 365 nm UV light for 1 h under optical microscopy (Figure S28a). After photoirradiation process, the macroscopic soft scaffold of **SA**_**Ala**_ was shrunken isotropically by less than 5% of original size (Figure S28b). In contrast, a macroscopic scaffold of **SA**_**Val**_ was shrunken isotopically by approximately 20% of the original size upon 365 nm irradiation (Figure S29). The results suggest that the isotropic shrinkage of macroscopic soft scaffolds of **SAs** can potentially be controlled by altering the packing structures of the macroscopic soft scaffolds. To enable a controllable isotropic shrinkage of macroscopic soft scaffolds of **SAs**, a coassembly strategy was applied to mix different ratios of **SA**_**Ala**_ and **SA**_**Val**_ in macroscopic soft scaffolds. The photoirradiation induced isotropic shrinkage of a macroscopic soft scaffold of **SA**_**Ala**_ : **SA**_**Val**_ (9:1) was found to remain stable (less than 5% volume reduction) upon 365 nm irradiation (Figure S30). A macroscopic soft scaffold of **SA**_**Ala**_ : **SA**_**Val**_ (1:1) was shrunken isotopically by 10% in volume upon 365 nm irradiation (Figure S31). The results suggest that by implementation of the **SA**_**Val**_ into **SA**_**Ala**_ enables the photoresponsiveness of the macroscopic soft scaffolds by disruption of the structural stability and supramolecular interactions of the **SA**_**Ala**_, which paves a way for the next generation design of controllable photoresponsive soft materials.

### 2.6 Cell‐Material Interface Study

To investigate the biocompatibility of nanotubes of **SA**_**Ala**_ at normal body temperature, stability tests were performed at 37°C for 12 h prior to MTS assay. UV–vis spectra and CD spectra of **SA**_**Ala**_ nanotubes showed no significant spectral change after incubation at 37°C for 12 h (Figure S30), suggesting that nanotubes formed by **SA**_**Ala**_ are stable at 37°C throughout the incubation period. TEM images of nanotubes of **SA**_**Ala**_ slightly were revealed to show nanotubes elongated for 12% (Figures S32), further verifying good structural stability of nanotubes formed by **SA**_**Ala**_. Aqueous solutions of **SA**_**Ala**_ and **SA**_**Val**_ were prepared after thermal annealing process and subjected for cytocompatibility studies. An MTS assay was employed for determining cell viability of HeLa cells in the presence of aqueous solution of **SA**_**Ala**_ and **SA**_**Val**_ in concentration range of 20–100 μM, respectively (Figure S33). The results were normalized to viability of HeLa cells in the absence of **SAs**. High cell viabilities (~90%) of HeLa cells over 24 h of culture were observed for both solutions of **SA**_**Ala**_ and **SA**_**Val**_ in concentration range of 20–100 μM. In this connection, both nanotubes of **SA**_**Ala**_ and **SA**_**Val**_ were revealed with good biocompatibility regardless of their structural variation from different steric bulkiness of the amino acid side chains. To explore potential applications of **SAs** as functional cell‐material interfaces, the cell adhesion ability on the macroscopic soft scaffolds of **SAs** were examined. The macroscopic soft scaffolds of **SA**_**Ala**_ (5 wt%, 65 mM, annealed at 85°C for 10 min) was prepared in calcium chloride solution (150 mM) and followed by washing with water. A growth medium containing HeLa cells was added to the macroscopic soft scaffolds of **SA**_**Ala**_. The mixture of macroscopic soft scaffolds of **SA**_**Ala**_ and HeLa cells was incubated for 3 days prior to imaging. According to the OM images (Figure S34a), rounded HeLa cells were found to show cell attachments onto the macroscopic s of **SA**_**Ala**_ as revealed by the Calcein AM stained HeLa cells emitted green fluorescence in the resulting fluorescent image (Figure S34b). The results indicated that the macroscopic soft scaffolds of **SA**_**Ala**_ have the potential to function as a cell‐culture platform, i.e., cell‐material interfaces. In contrast, the macroscopic soft scaffolds of **SA**_**Val**_ were destabilized in the growth medium, suggesting that **SA**_**Val**_ is not applicable to function as cell‐material interfaces. Besides, with reference to electron microscopic and WAXD analysis, the nanostructures and packing structure of macroscopic soft scaffolds of **SA**_**Val**_ were confirmed with lower packing structural order than that observed in **SA**_**Ala**_. The results suggested that the structural stability of the macroscopic soft scaffolds of **SA**_**Ala**_ is adequate for the basic requirements as cell‐material interfaces.

## 3 Conclusion

In summary, our study underscored the critical role of molecular design in development of robust supramolecular systems with exceptional structural stability. Through meticulous analyses using circular dichroism spectroscopy and electron microscopy, we demonstrated that nanotubes formed by stiff‐stilbene amphiphile bearing an alanine terminal group (**SA**_**Ala**_) exhibited extraordinary structural stability in aqueous medium when subjected to various external stimulus at microscopic scale. Specifically, the molecular design of **SA**_**Ala**_, featured with advanced *π–π* interaction between stiff‐stilbene cores with amide hydrogen bonding that governs the intermolecular distance, enabled the formations of supramolecular nanostructures with outstanding pH stability and thermal stability at 95°C, achieved solely through supramolecular interactions without reliance on covalent backbones. X‐ray diffraction studies of the macroscopic soft scaffolds of **SA**_**Ala**_ further revealed that higher‐order tubular packing within the nanotubes of **SA**_**Ala**_ is the key factor contributing to the extraordinary resistance to structural deformations under external stimulations. Notably, we demonstrated that structural stabilities of these assemblies can be fine adjusted by increasing the steric bulkiness of the amino‐acid side chain from methyl‐group to isopropyl‐group, thereby modulating the structural stability of both nanostructures and macroscopic soft scaffolds in response to light stimulations at macroscopic length‐scale. In addition, macroscopic soft scaffold of **SA**_**Ala**_ exhibited good biocompatibility to function as a cell‐material interfacial platform. By elucidation of the fundamental principles of structural design in stable supramolecular amphiphilic systems, our findings could open up new prospects towards the development of biocompatible robust supramolecular materials.

## Supporting Information

Additional supporting information can be found online in the Supporting Information section.

## Funding

This study was supported by the Croucher Foundation (Croucher Innovation Award‐2021), the Hong Kong Research Grants Council General Research Fund (GRF 15305822), the Hong Kong Polytechnic University (BC7W, CDMU, WZCT, WZCA), the Hong Kong Special Administrative Region Government (InnoHK) Centre for Eye and Vision Research (CEVR), and the CREST, Japan Science and Technology Agency (JPMJCR23L2).

## Conflicts of Interest

The authors declare no conflicts of interest.

## Supporting information

Supplementary Material

## Data Availability

The data that support the findings of this study are available in the Supporting Information of this article.
