# Supplementary material for: Engineering Robust Supramolecular Nanoassemblies from Amino‐Acid Functionalized Stiff‐Stilbene Amphiphiles
Source: Small Sci. 2026 May 11;6(5):e70305. doi: 10.1002/smsc.70305 (PMC13165923; doi:10.1002/smsc.70305)
Supplement: Supplementary file 1 — Supplementary Material [file SMSC-6-e70305-s001.pdf]

Supplementary Information

Engineering Robust Supramolecular Nanoassemblies from Amino-Acid Functionalized Stiff-Stilbene Amphiphiles

Khloe Shuk-Ying Kwan†, Ming-Hin Chau†, Wai-Ki Wong, Takashi Kajitani, Franco King-Chi Leung*

E-mail: kingchifranco.leung@polyu.edu.hk

Table of Contents

1. Materials and Methods 2
2. Synthesis 6
3. Supporting Figures 10
4. Analytical Data 44

1. Materials and Methods

Materials

All commercial reagents were purchased from Acros Organics, Aladdin, Alfa Aesar, Bidepharm, Dieckmann, Macklin, Sigma Aldrich and Tokyo Chemical Industry Co. Ltd, and were used as received. All solvents used in the reactions were purchased from Acros, Anaqua and Duksan. Analytical thin layer chromatography (TLC) was performed on ALUGRAM Xtra SIL G UV254 plates and visualized by UV light or staining with phosphomolybdic acid (PMA) solution followed by heating. Flash column chromatography was performed using Macherey-Nagel Silica gel 60 (230–400 mesh). Deuterated solvents were purchased from Cambridge Isotope Laboratories Inc.

General Characterization

NMR spectra were recorded at 25 °C on a Bruker Advance-III 400 MHz FT-NMR spectrometer (^{1}H: 400 MHz, ^{13}C: 101 MHz) and a Bruker Advance-III 600 MHz FT-NMR spectrometer (^{1}H: 600 MHz, ^{13}C: 151 MHz). The deuterated DMSO-d_6 was degassed by argon prior to use. Chemical shifts (δ) are expressed relative to the resonances of the residual non-deuterated solvent for ^{1}H NMR [$CDCl_3$: ^{1}H(δ) = 7.26 ppm, DMSO-d_6: ^{1}H(δ) = 2.50 ppm] and ^{13}C NMR [$CDCl_3$: ^{13}C(δ) = 77.2 ppm, DMSO-d_6: ^{13}C (δ) = 39.5 ppm]. Absolute values of the coupling constants are given in Hertz (Hz), regardless of their sign. Multiplicities are abbreviated as singlet (s), doublet (d), doublet of doublets (dd), triplet (t), triplet of doublets (td), quartet (q), multiplet (m), and broad (br). High-resolution mass spectrometry (HRMS) was performed on an Agilent 6540 LC-ESI Quadrupole-Time-of-Flight Mass Spectrometer.

UV-vis Absorption Spectroscopy

UV-vis measurements were performed on an Agilent Cary 60 UV-Vis spectroscopy in a 1.0 cm path length quartz cuvette. Irradiation of samples in organic media were conducted at 293 K using Thorlabs M365F1 LED (4.1 mW) and M385F1 LED (10.7 mW) positioned at a distance of 1.0 cm from the samples. Irradiation of samples in aqueous media were carried out by using Thorlabs M365LP-C1 (745 mW), M385LP-C1 (795 mW) and M405LP-C1 (750 mW) at 25 °C.

Circular Dichroism

Circular dichroism (CD) measurement was performed on a JASCO J-1500 CD Spectrophotometer at 25 °C in a 1.0 mm path length quartz cuvette. CD spectra were recorded in the range of 190–450 nm. A scanning rate of 200 nm/min, a bandwidth of 1.0 nm, a data pitch of 1.0 nm, and single accumulation were applied.

Transmission Electron Microscopy

To study the morphology of self-assembled **SAs**, the sample solutions (5.0 μL) were deposited onto a carbon grid (Micro to Nano, EMR Carbon support film on copper, 400 square mesh) for 20 s. After blotting, UranyLess EM stain solution (Electron Microscopy Science, 5.0 μL) was directly deposited onto the grid for 20 s and blotting to remove the stain. Grids were observed in a JEOL Model JEM-2010 Transmission Electron Microscope with hair pin type tungsten filament operating at 120 kV equipped with Gatan 794 CCD camera or JEOL Model JEM-2100F operating at 200 kV FE (field emission) analytical electron microscope.

Scanning Electron Microscopy and Polarized Optical Microscopy Analysis

Polarized optical microscopy (POM) was performed on a Leica DM2700-P optical polarizing microscope. Scanning electron microscopy (SEM) was performed on a Tescan VEGA3 Scanning Electron Microscope. Preparation of a string of $\mathbf{SA_{Ala}}$ on a glass substrate: $\mathbf{SA_{Ala}}$ (5 wt.%, 65 mM, 20 μL) with seven equivalents of NaOH was annealed at 85 °C for 10 min, then cooled down to room temperature to form a milky solution. When an aqueous solution of $\mathbf{SA_{Ala}}$ (5.0 wt.%, 65 mM, 2 μL) was manually drawn into an aqueous solution of $CaCl_2$ (150 mM) from a pipette, a string with arbitrary length was formed. After removal of the solution of metal chloride, the string was washed with MilliQ water (three times), the resulting string was used directly for POM and SEM experiments. A string for SEM was directly prepared on conductive carbon adhesive tape and dried in air for 72 h before measurement. The air-dried samples were subject to gold sputtering (MCM-200 ion sputter coater) for 30 min prior to SEM measurement.

Static Dynamic Light Scattering

The scattering intensities of the samples were determined by dynamic light scattering measurement on a Wyatt Technology DynaPro NanoStar. The scattering intensities were recorded as a parameter for assembly size, since the objects in solutions are anisotropic and the models used by Wyatt software are fitting for spherical objects. To determine the critical aggregation concentration (CAC) of **SAs**, the scattering intensities of the solutions of **SA$_{\mathbf{Ala}}$** (concentration: 5.0 x 10^{-4} to 0.08 mM) were recorded at 20 °C. This scattering rate was normalized by the concentration of the solution to yield the molar scattering intensity (on M Counts s^{-1} M^{-1}). Five replications were performed and the data was averaged to show the molar scattering intensity and its error standard deviation.

Small-Angle X-ray Scattering and Wide-Angle X-ray Diffraction

Small-angle X-ray scattering (SAXS) and wide-angle X-ray diffraction of a string of **SAs** (5.0 wt.%, 65 mM) were measured on a sapphire substrate (φ = 2.0 cm) using the Rigaku NANOPIX equipped with a HyPix-6000 (Rigaku) detector. The scattering vector ($q = 4\pi\sin\theta/\lambda$), scattering angle θ and the position of the incident X-ray beam on the detectors were calibrated using several orders on layer reflections from silver behenate (d = 58.380 Å), where λ refers to the wavelength of the X-ray beam (Cu$K\alpha$, 1.54 Å). The sample-to-detector distances were 729 mm for SAXS and 101 mm for WAXD. The obtained diffraction patterns were integrated along the Debye-Scherrer ring to afford 1D intensity data using the Rigaku 2DP software.

MTS Assay for Cell Viability

HeLa cell was cultured in growth medium: Dulbecco's Modified Eagle Medium (DMEM, Gibco™ thermofisher:11965092) supplemented with Fetal bovine serum (1:10 to DMEM, Gibco™ thermofisher:10270106) and Antibiotic-Antimycotic (1.1:100 to DMEM, Gibco™, thermofisher:15240062), under 37 °C, 5% CO_2. 5,000 HeLa cells were seeded on 96-well. For the treatment, cells were incubated with 140 μL of **SA$_{\mathbf{s}}$** in growth medium for one day. After that, each well received 28 μL MTS solution (CellTiter 96® AQueous One Solution Cell Proliferation Assay, Promega) and further incubated for one hour. Absorbance at 492 nm of the samples was measured via Labexim Products LEDETECT 96 Microplate Reader, referenced with 620 nm (n = 3).

Cytotoxicity Studies of Macroscopic Strings

The hydrogel was prepared by injecting 1 μL of **SA$_{\mathbf{Ala}}$** (5 wt.%, 65 mM) in 150 mM $CaCl_2$ on bioinert culture dish (μ-Dish. ibidi 81150). Hydrogels were prepared in each dish. 2.5 mL growth medium containing 500,000 HeLa cells was added to each bioinert culture dish and the sample was incubated for 3 days. The sample was incubated for 3 days. After that, 1 mL medium was extracted and 1 mL growth medium with 1 μM Calcein AM (Invitrogen™, thermofisher: C3100MP) was added to the samples. Live HeLa cells were labelled by incubating for 30 min. The Calcein AM containing medium was extracted and growth medium was added to the sample. Finally, the growth medium was removed and the samples were imaged under Leica DM 2700P.

2. Synthesis

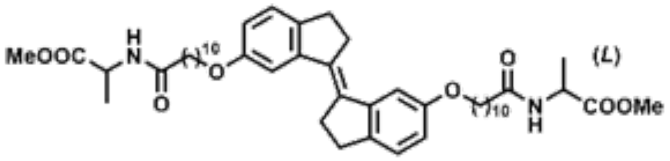

Compound **1**

A mixture of **5SA** (100 mg, 0.16 mmol), *L*-alanine methyl ester hydrochloride (43 mg, 0.31 mmol), hexafluorophosphate benzotriazole tetramethyl uronium (136 mg, 0.36 mmol), hydroxybenzotriazole monohydrate (48 mg, 0.36 mmol) and *N,N*-diisopropylethylamine (98 μL, 0.56 mmol) in dimethylformamide (5 mL) was stirred at 20 °C for 24 h. The reaction mixture was then washed with water (40 mL x 3) and ethyl acetate (40 mL x 3). The combined organic layers were washed with brine, dried over Mg_2SO_4, and the solvent was evaporated at reduced pressure. The residue was recrystallized in ethyl acetate/hexane (1:9) to afford *trans*-**1** as an off-white solid (53 mg, 0.07 mmol, 42%).

^{1}H NMR (600 MHz, $CDCl_3$) δ 7.20 (d, J = 8.2 Hz, 2H), 7.16 (d, J = 2.3 Hz, 2H), 6.78 (m, 2H), 5.98 (d, J = 7.5 Hz, 2H), 4.61 (m, 2H), 3.99 (t, J = 6.6 Hz, 4H), 3.75 (s, 6H), 3.18 (m, 4H), 3.09 – 2.99 (m, 4H), 2.24 – 2.14 (m, 4H), 1.79 (m, 4H), 1.63 (m, 5H), 1.58 (s, 17H), 1.47 (m, 4H), 1.40 (d, J = 7.2 Hz, 6H), 1.30 (m, 20H).

^{13}C NMR (151 MHz, $CDCl_3$) δ 173.8, 172.6, 158.1, 144.4, 139.3, 135.7, 125.2, 113.5, 111.0, 68.4, 52.5, 47.9, 36.6, 32.5, 30.2, 29.5, 29.4, 29.3, 29.2, 26.1, 25.6, 18.6.

HR-MS (ESI+) calculated for $C_{48}H_{70}N_2O_8$ $[M+H]^+$ 803.5205, found 803.5204.

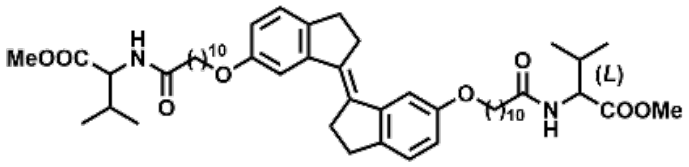

Compound **2**

A mixture of **5SA** (100 mg, 0.16 mmol), *L*-valine methyl ester hydrochloride (52 mg, 0.31 mmol), hexafluorophosphate benzotriazole tetramethyl uronium (136 mg, 0.36 mmol), hydroxybenzotriazole monohydrate (48 mg, 0.36 mmol) and *N,N*-diisopropylethylamine (98 μL, 0.56 mmol) in dimethylformamide (5 mL) was stirred at 20 °C for 24 h. The reaction mixture was then washed with water (40 mL x 3) and ethyl acetate (40 mL x 3). The combined organic layers were washed with brine, dried over Mg_2SO_4, and the solvent was evaporated at reduced pressure. The residue was recrystallized in ethyl acetate/hexane (1:9) to afford *trans*-**2** as an off-white solid (98 mg, 0.1 mmol, 72%).

^{1}H NMR (400 MHz, $CDCl_3$) δ 7.20 (d, J = 8.2 Hz, 2H), 7.16 (d, J = 2.3 Hz, 2H), 6.77 (dd, J = 8.2, 2.3 Hz, 2H), 5.90 (d, J = 8.9 Hz, 2H), 4.59 (dd, J = 8.8, 4.9 Hz, 2H), 3.99 (t, J = 6.6 Hz, 4H), 3.74 (s, 6H), 3.18 (m, 4H), 3.05 (m, 3H), 2.28 – 2.20 (m, 4H), 2.15 (td, J = 6.9, 5.0 Hz, 2H), 2.05 (s, 1H), 1.79 (m, 4H), 1.64 (m, 4H), 1.59 (s, 12H), 1.53 – 1.40 (m, 4H), 1.39 – 1.21 (m, 20H), 0.92 (dd, J = 14.2, 6.9 Hz, 12H).

^{13}C NMR (101 MHz, $CDCl_3$) δ 173.4, 172.7, 158.1, 144.4, 135.7, 126.6, 126.5, 125.2, 116.8, 113.5, 111.5, 111.0, 68.4, 56.9, 52.2, 36.8, 32.5, 31.3, 30.2, 29.5, 29.4, 29.3, 29.2, 26.1, 25.7, 18.9, 17.8.

HR-MS (ESI+) calculated for $C_{52}H_{78}N_2O_8$ $[M+H]^+$ 859.5831, found 859.5832.

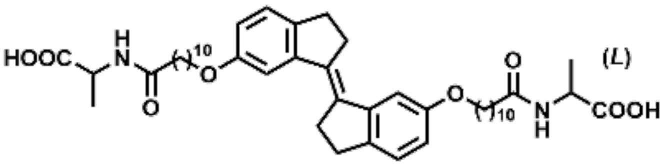

SA$_{Ala}$

To a solution of compound **1** (30 mg, 0.04 mmol) in methanol (3 mL) and tetrahydrofuran (3 mL), an aqueous NaOH solution (4 M, 2 mL) was added and the reaction mixture was then heated at 80 °C for 1 h. The reaction mixture was cooled down to 20 °C and the solvent was evaporated under reduced pressure. The reaction mixture was neutralized to pH~7 by adding HCl solution (1 M), off-white precipitate was then formed. The filtered precipitate was washed with H_2O (10 mL) to afford an off-white solid (11 mg, 0.01 mmol, 38%).

^{1}H NMR (600 MHz, DMSO-d_6) δ 7.87 (s, 2H), 7.21 (d, J = 8.4 Hz, 2H), 7.07 (s, 2H), 6.80 (d, J = 8.4 Hz, 2H), 4.05 (s, 2H), 3.98 (s, 4H), 3.10 (s, 4H), 2.98 (s, 4H), 2.18 (t, J = 7.5 Hz, 2H), 2.06 (s, 4H), 1.71 (s, 5H), 1.52 – 1.38 (m, 11H), 1.37 – 1.08 (m, 40H).

^{13}C NMR (151 MHz, DMSO-d_6) δ 174.6, 174.4, 171.8, 157.7, 143.8, 138.8, 135.3, 125.3, 113.9, 110.2, 69.8, 67.6, 47.7, 35.1, 33.7, 31.8, 29.7, 29.0, 28.9, 28.8, 28.8, 28.6, 28.6, 25.6, 25.3, 24.5, 22.1, 17.6.

HR-MS (ESI+) calculated for $C_{46}H_{66}N_2O_8$ $[M+H]^+$ 775.4892, found 775.4909.

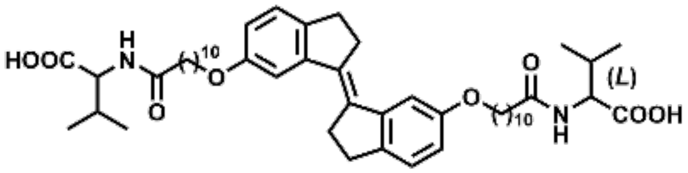

SA$_{Val}$

To a solution of compound **2** (41 mg, 0.04 mmol) in methanol (3 mL) and tetrahydrofuran (3 mL), an aqueous NaOH solution (4 M, 2 mL) was added and the reaction mixture was then heated at 80 °C for 1 h. The reaction mixture was cooled down to 20 °C and the solvent was evaporated under reduced pressure. The reaction mixture was neutralized to pH~7 by adding HCl solution (1 M), off-white precipitate was then formed. The filtered precipitate was washed with H_2O (10 mL) to afford an off-white solid (20 mg, 0.02 mmol, 69%).

^{1}H NMR (600 MHz, DMSO-d_6) δ 7.79 (s, 2H), 7.22 (d, J = 8.4 Hz, 2H), 7.07 (s, 2H), 6.80 (s, 2H), 4.07 (s, 2H), 3.98 (s, 4H), 3.10 (s, 4H), 2.98 (s, 4H), 2.20 – 2.07 (m, 6H), 2.01 (d, J = 7.9 Hz, 3H), 1.71 (s, 6H), 1.54 – 1.37 (m, 10H), 1.25 (m, 27H), 0.83 (d, 16H).

^{13}C NMR (151 MHz, DMSO-d_6) δ 173.3, 172.5, 137.8, 127.8, 123.7, 105.4, 67.8, 57.1, 54.9, 36.6, 35.0, 31.0, 29.8, 29.0, 28.9, 28.8, 28.6, 25.4, 24.7, 22.1, 19.2, 18.1, 14.0.

HR-MS (ESI+) calculated for $C_{50}H_{74}N_2O_8$ $[M+H]^+$ 831.5518, found 831.5509.

3. Supporting Figures

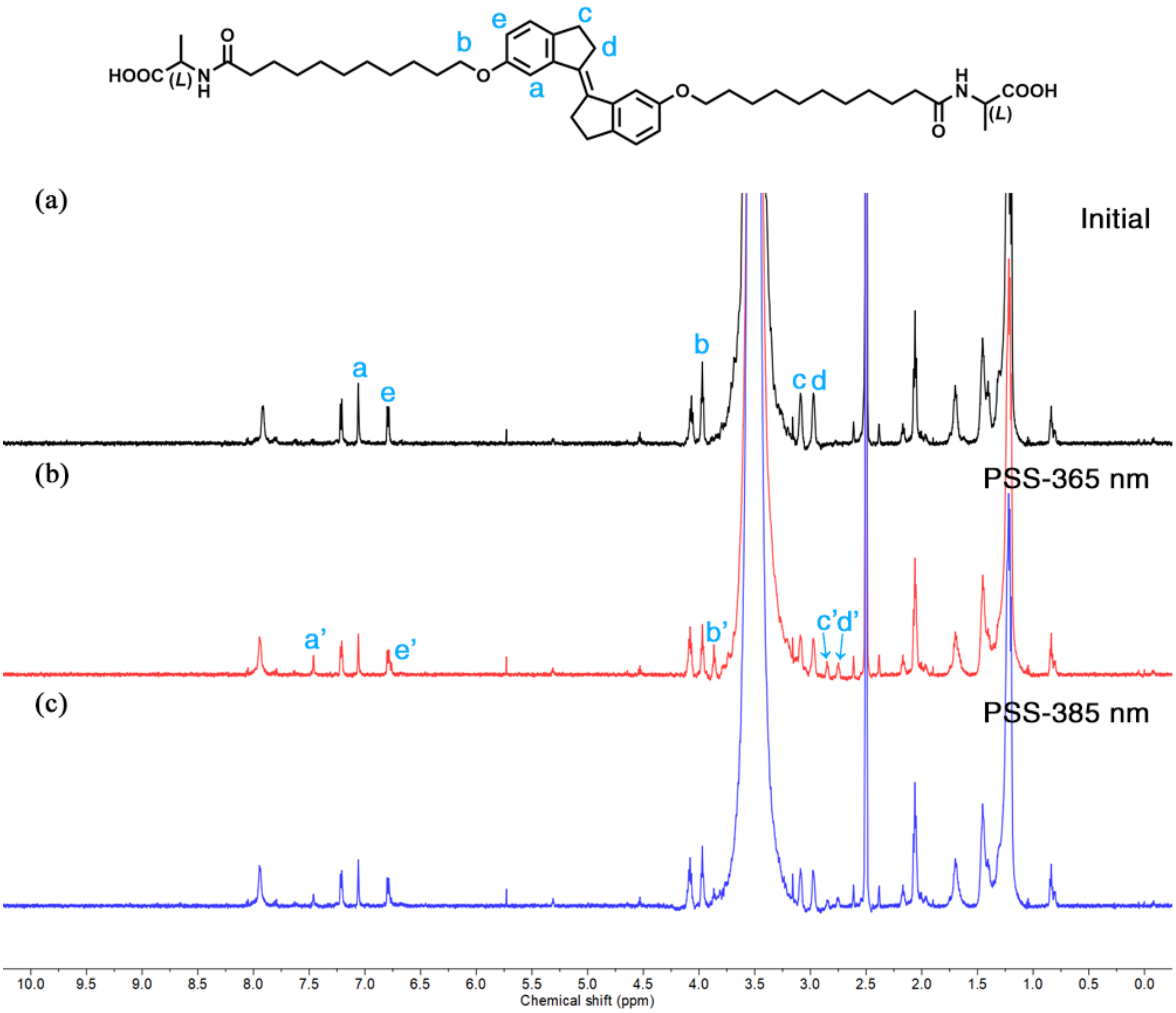

Figure S1: ^{1}H NMR spectra during isomerization process of **SA$_{Ala}$** (5.5 mM, DMSO-*d*$_6$, 600 MHz), (a) before photoirradiation, (b) irradiated with 365 nm for 1 h at 20 °C, (c) followed by 385 nm irradiation for 1 h at 20 °C. Irradiation of sample was carried out at 20 °C using a Thorlabs model fiber-coupled high-power LED (M365F1, 365 nm, 0.7 A; M385F1, 385 nm, 0.7 A) positioned at a distance of 1 cm from the sample. PSS$_{365}$ ratio of *trans*-**SA$_{Ala}$**/*cis*-**SA$_{Ala}$** was 75:25 and PSS$_{385}$ ratio of *trans*-**SA$_{Ala}$**/*cis*-**SA$_{Ala}$** was 85:15. The ^{1}H NMR signals at 0.77 – 0.86 ppm are from grease oil; 5.7 ppm is originated from DCM.

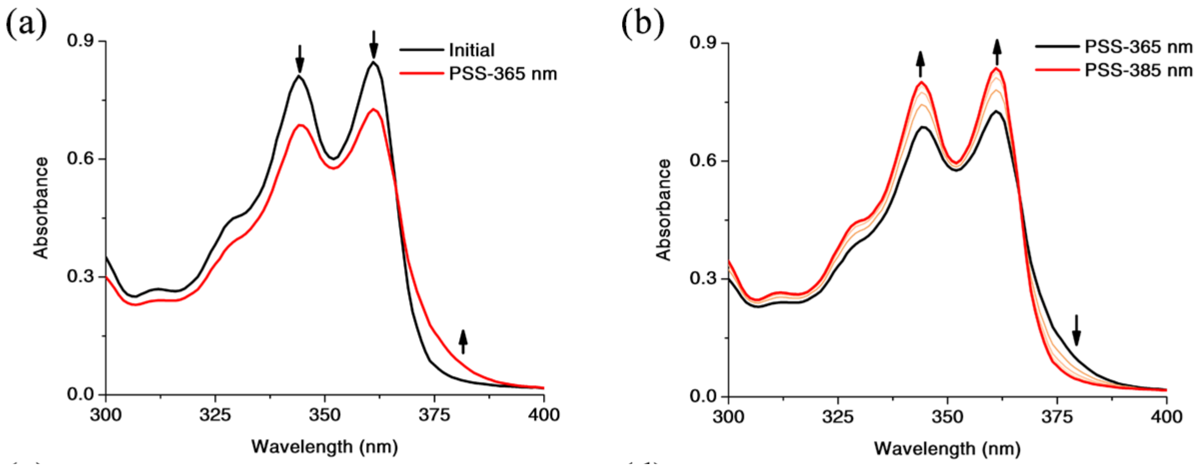

Figure S2: UV-vis absorption spectra of the $\mathbf{SA_{Val}}$ in DMSO (90 μM). (a) Absorption spectra of $\mathbf{SA_{Val}}$ in PSS (red) after 365 nm irradiation for 1 min, (b) in PSS (red) after 385 nm irradiation around 2 min (orange).

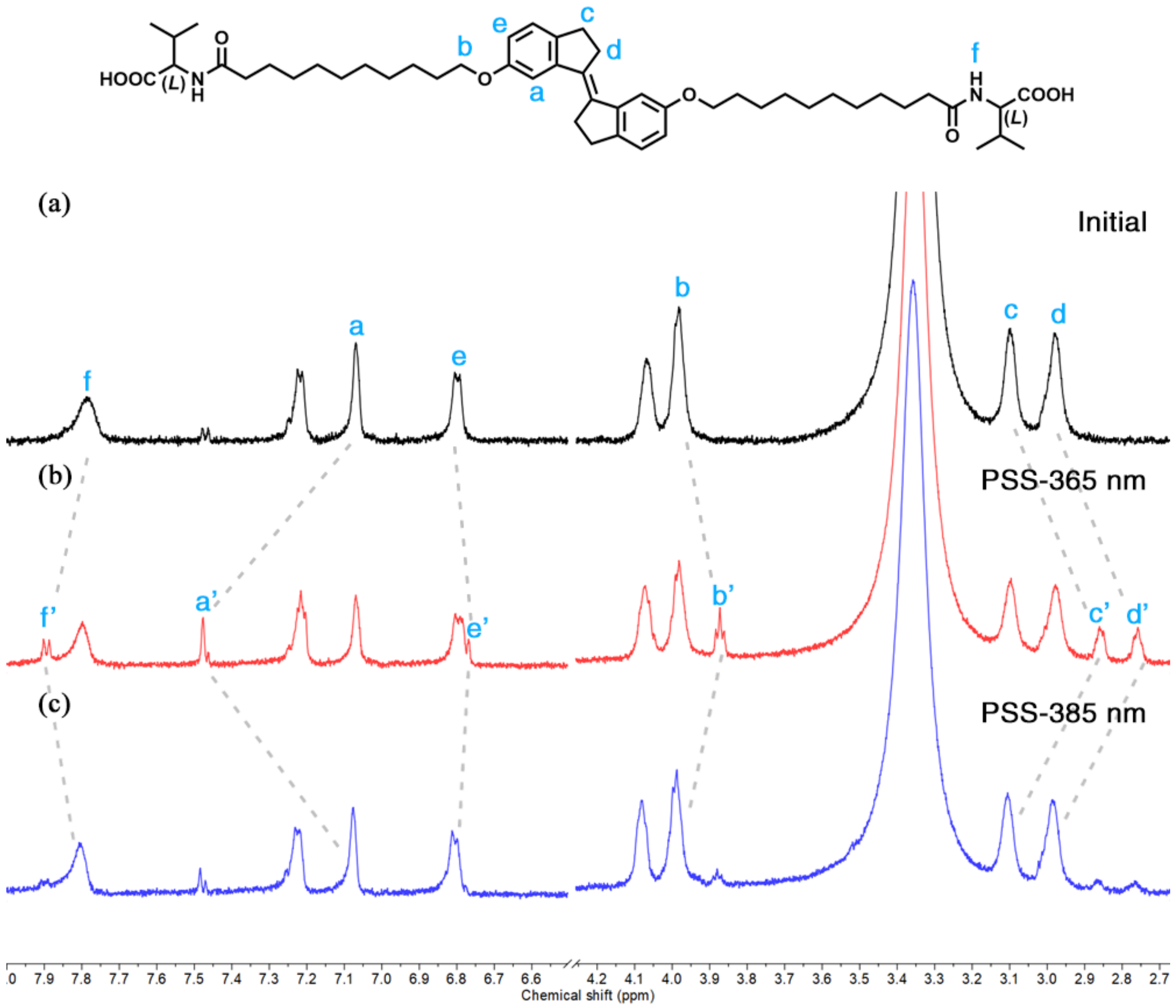

Figure S3: Aromatic and aliphatic region of ^{1}H NMR spectra during isomerization process of **SA**$_{\mathbf{Val}}$ (5.5 mM, DMSO-d_6, 600 MHz), (a) before photoirradiation, (b) irradiated with 365 nm for 1 h at 20 °C, (c) followed by 385 nm irradiation for 2 h at 20 °C. Irradiation of sample was carried out at 20 °C using a Thorlabs model fiber-coupled high-power LED (M365F1, 365 nm, 0.7 A; M385F1, 385 nm, 0.7 A) positioned at a distance of 1.0 cm from the sample. PSS_{365} ratio of *trans*-**SA**$_{\mathbf{Val}}$/*cis*-**SA**$_{\mathbf{Val}}$ was 74:26 and PSS_{385} ratio of *trans*-**SA**$_{\mathbf{Val}}$/*cis*-**SA**$_{\mathbf{Val}}$ was 90:10.

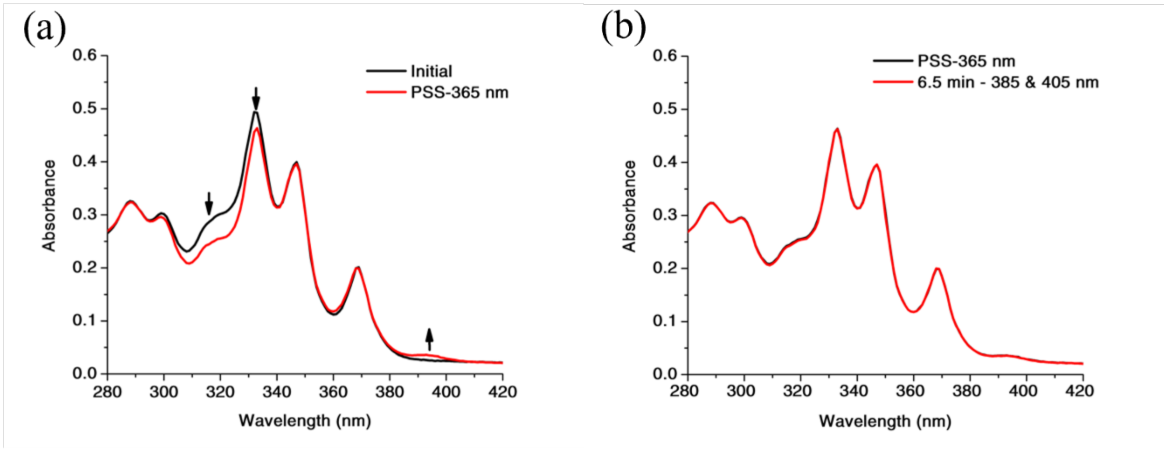

Figure S4. UV-vis absorption spectra of **SA$_{\mathbf{Ala}}$** in water (0.22 mM, pH = 9). (a) Absorption spectra of **SA$_{\mathbf{Ala}}$** in PSS (red) after 365 nm irradiation over 6.5 min (orange) (b) after a combination of 385 nm and 405 nm irradiation for 4 min (red). Irradiation of UV-vis sample was carried out at 20 °C using Thorlabs model collimated LEDs (M365LP-C1, 365 nm, 1.2 A; M385LP-C1, 385 nm, 1.2 A and 405 nm, M405LP-C1, 1.2 A) positioned at a distance of 1.0 cm from the sample.

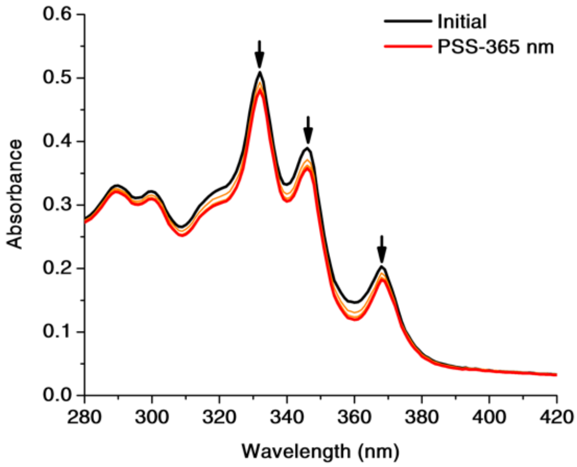

Figure S5. UV-vis absorption spectra of $\mathbf{SA_{Val}}$ in water (0.22 mM, pH = 9) after 365 nm irradiation over 10 min (orange). Irradiation of UV-vis sample was carried out at 20 °C using a Thorlabs model collimated LEDs (M365LP-C1, 365 nm, 1.2 A) positioned at a distance of 1.0 cm from the sample.

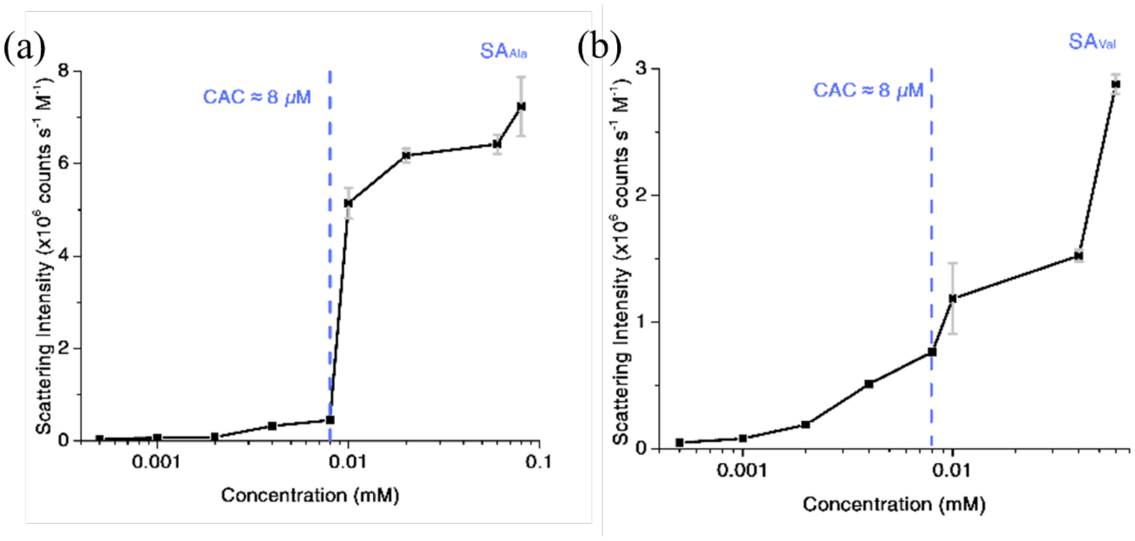

Figure S6. Static dynamic light scattering assay for determination of critical aggregation concentration of (a) **SA_{Ala}** (concentration: 5.0 x 10^{-4} to 0.08 mM) and (b) **SA_{Val}** (concentration: 5.0 x 10^{-4} to 0.06 mM).

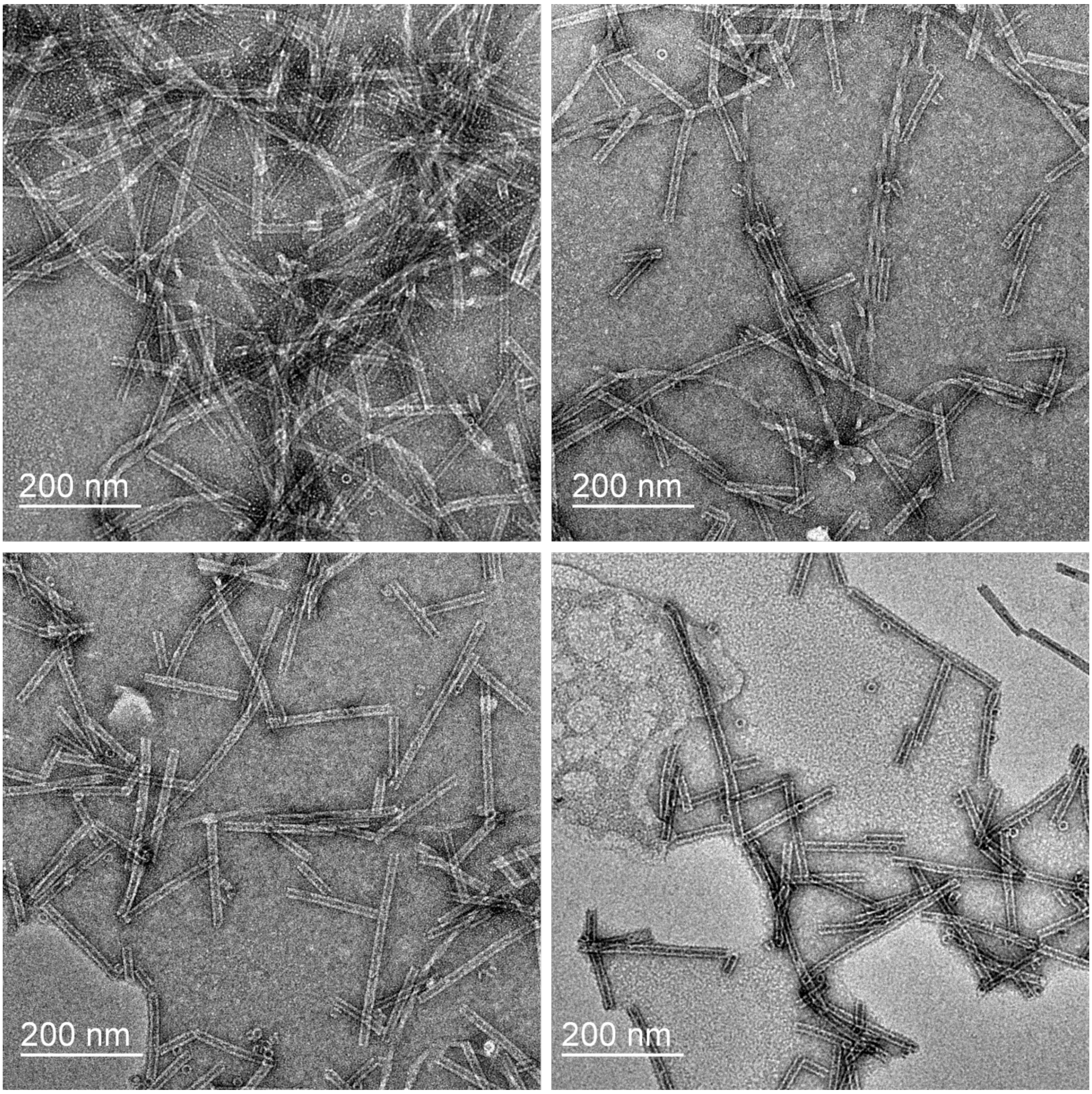

Figure S7. TEM images of $\mathbf{SA_{Ala}}$ (0.2 wt.%, 2.58 mM) in water at pH = 7.

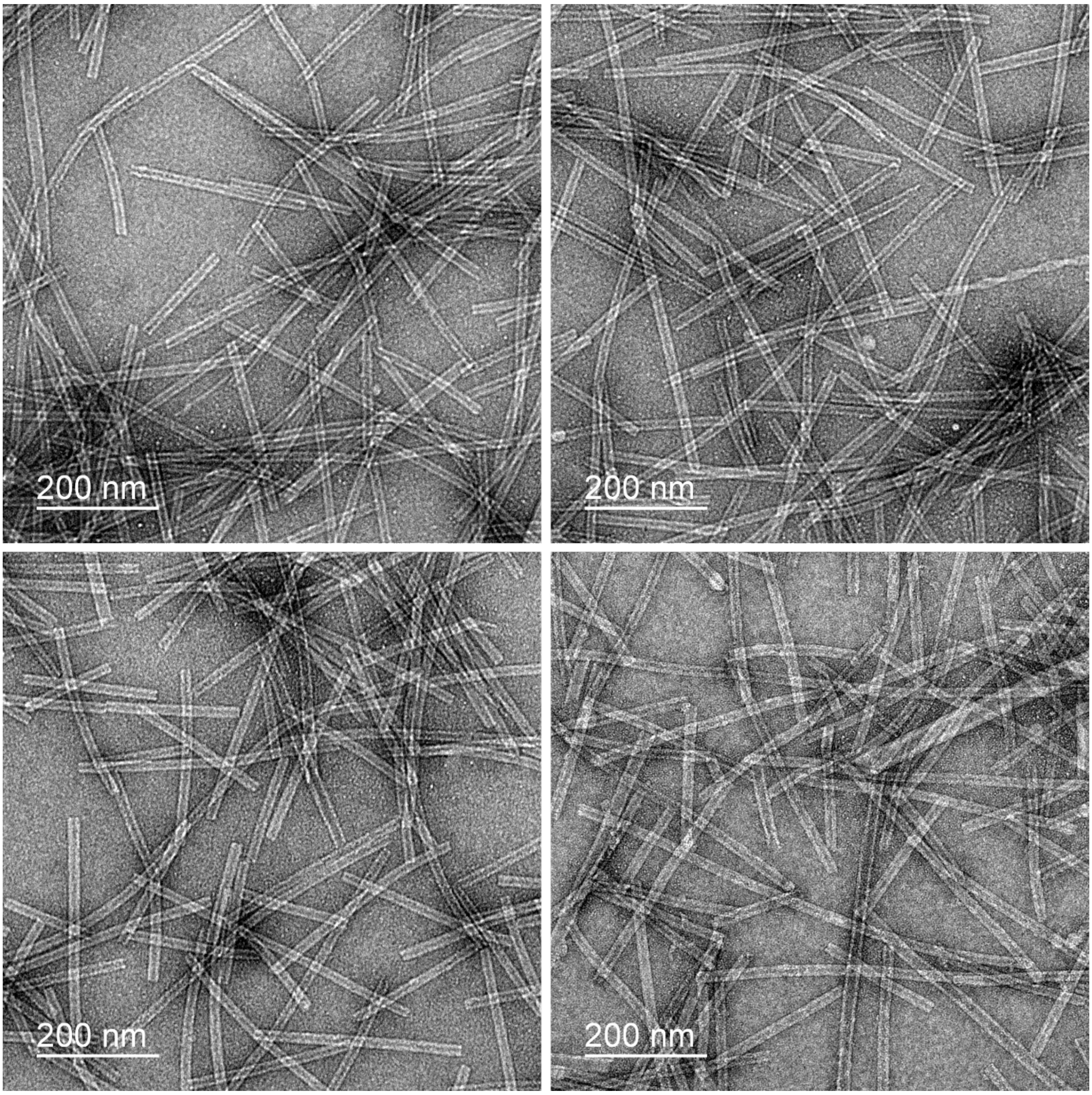

Figure S8. TEM images of $\mathbf{SA_{Ala}}$ (0.2 wt.%, 2.58 mM) in water at pH = 9.

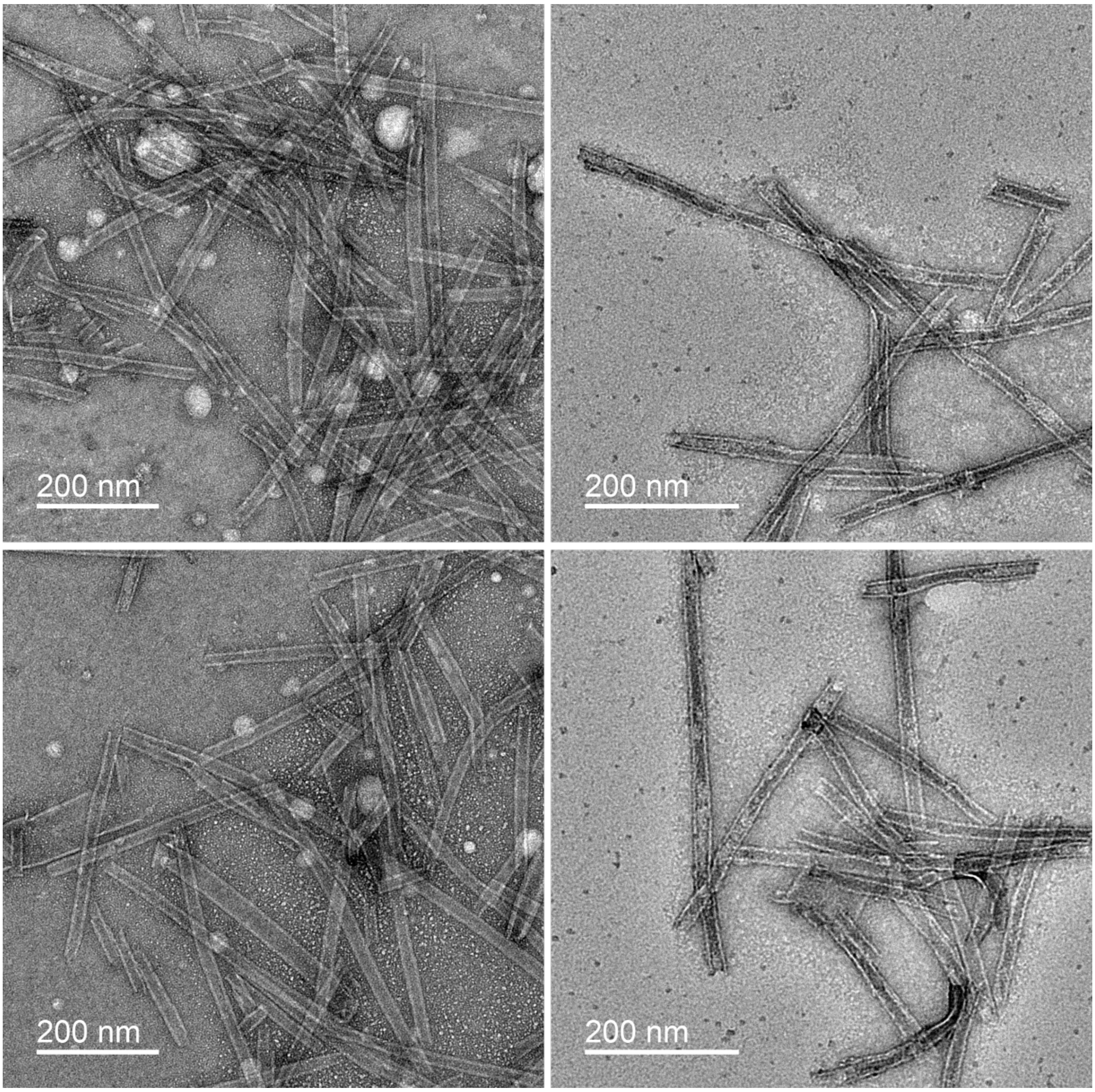

Figure S9. TEM images of $\mathbf{SA_{Ala}}$ (0.2 wt.%, 2.58 mM) in water at pH = 11.

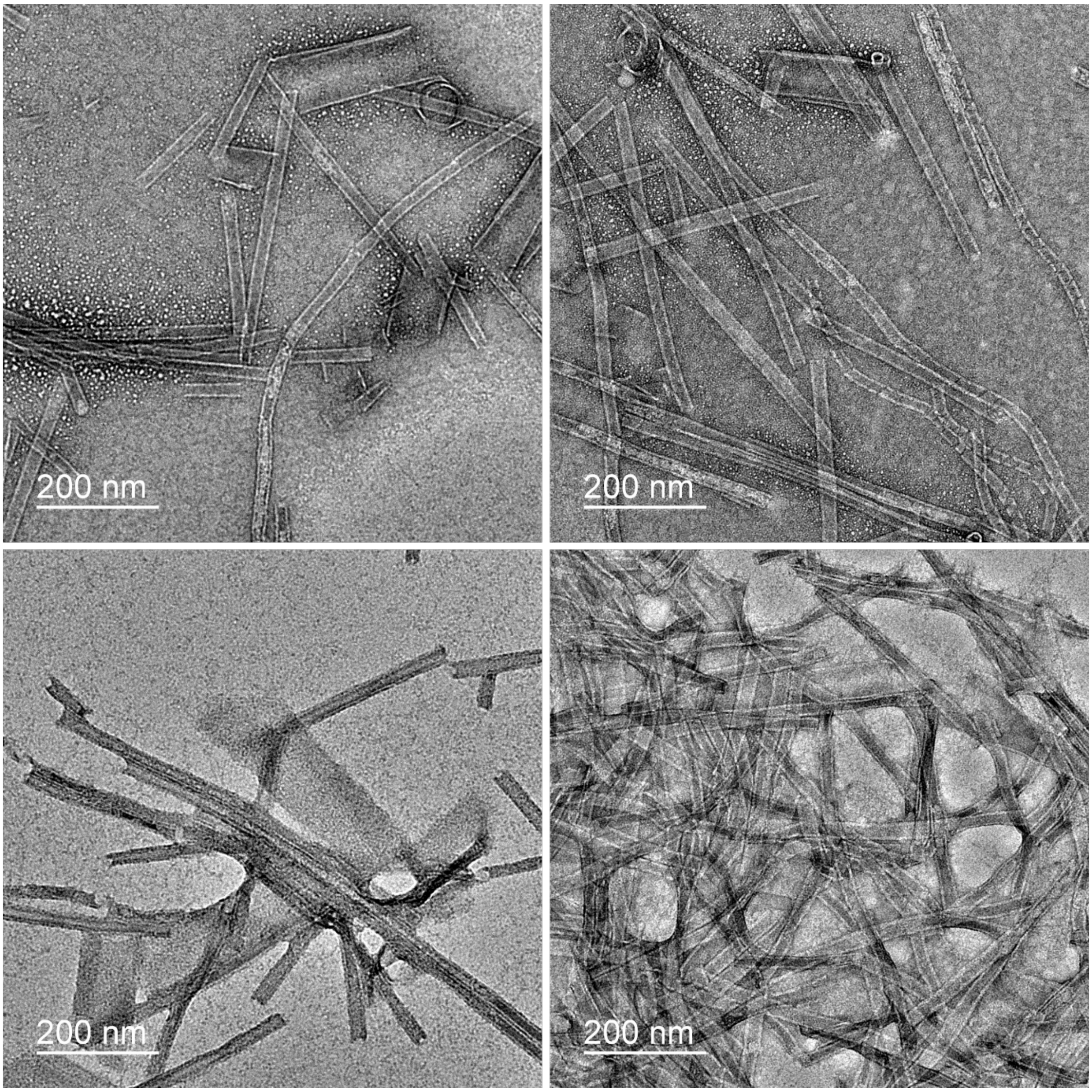

Figure S10. TEM images of **SA$_{\mathbf{Ala}}$** (0.2 wt.%, 2.58 mM) in water at pH = 13.

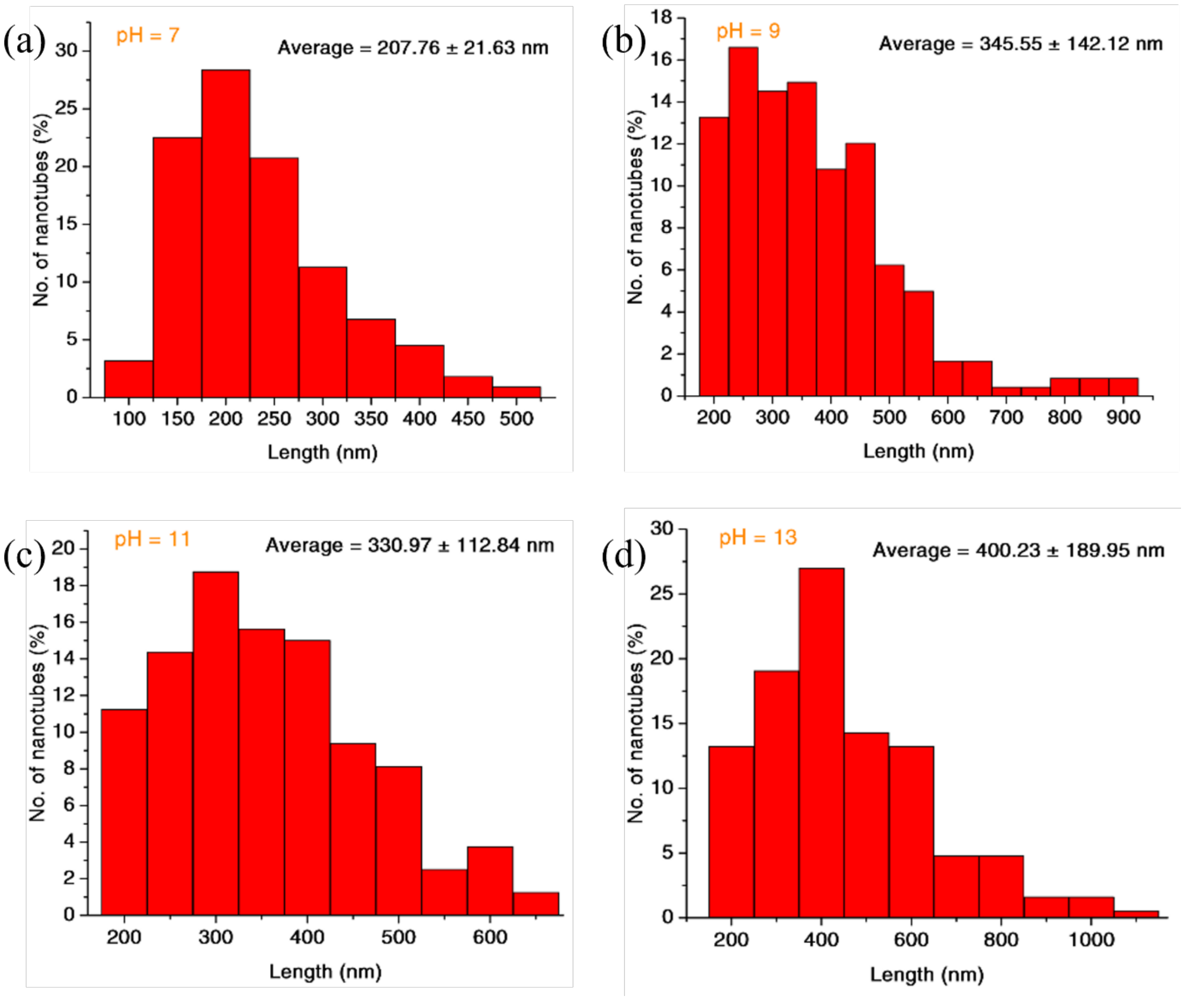

Figure S11. Histograms of length distribution of $\mathbf{SA_{Ala}}$ nanotubes formed in water at (a) pH = 7, (b) pH = 9, (c) pH = 11, and (d) pH = 13, respectively.

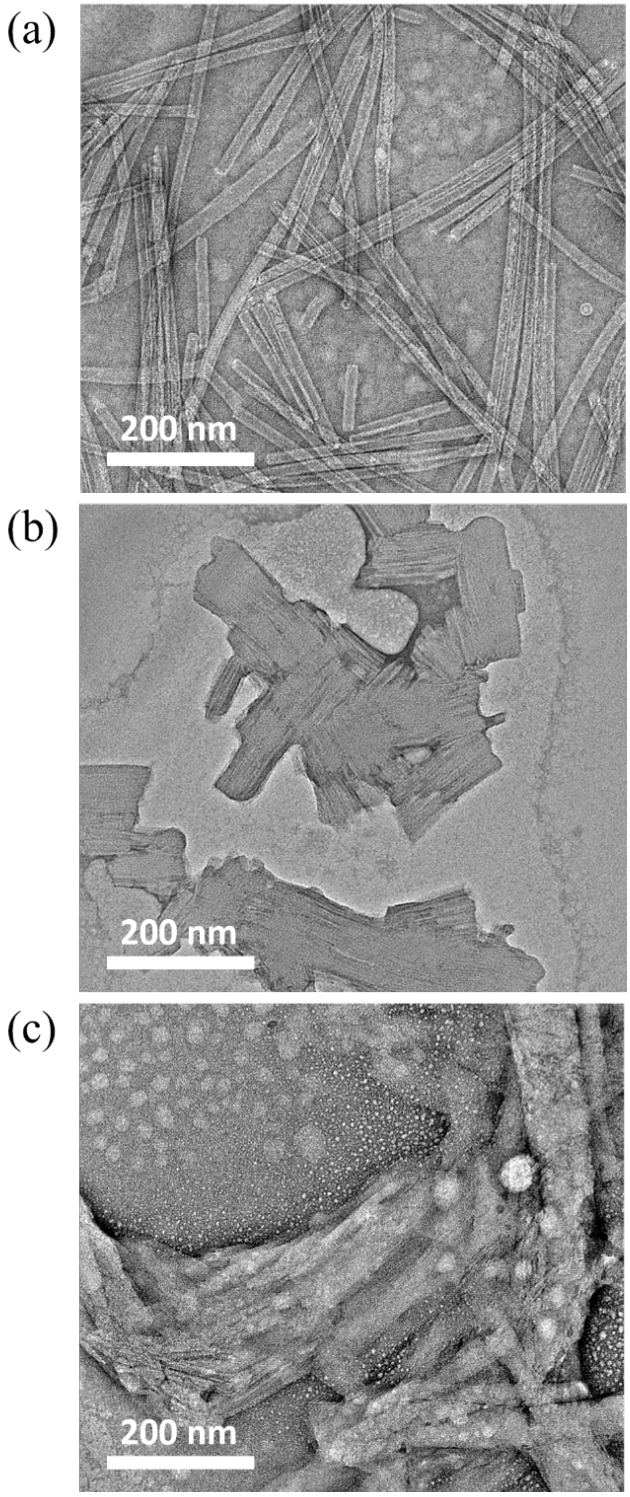

Figure S12. TEM image of $\mathbf{SA_{Ala}}$ (0.2 wt.%, 2.58 mM) in water at pH = 9 after addition of (a) 2 equivalents of NaCl, (b) 4 equivalents of NaCl, and (c) 2 equivalents of $CaCl_2$, respectively.

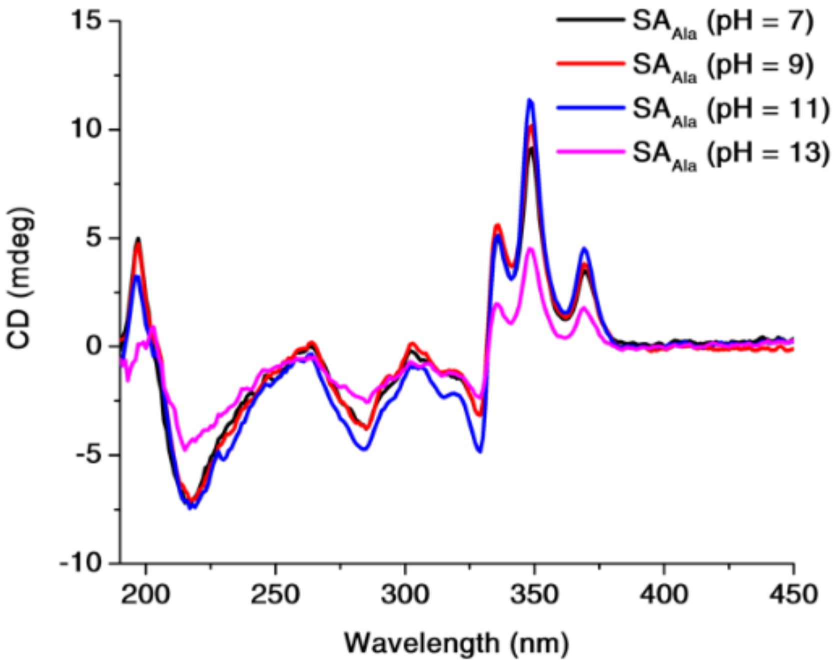

Figure S13. CD spectra of annealed **SA$_{Ala}$** (50 μM) in water at pH = 7, 9, 11, and 13.

(a) (b)

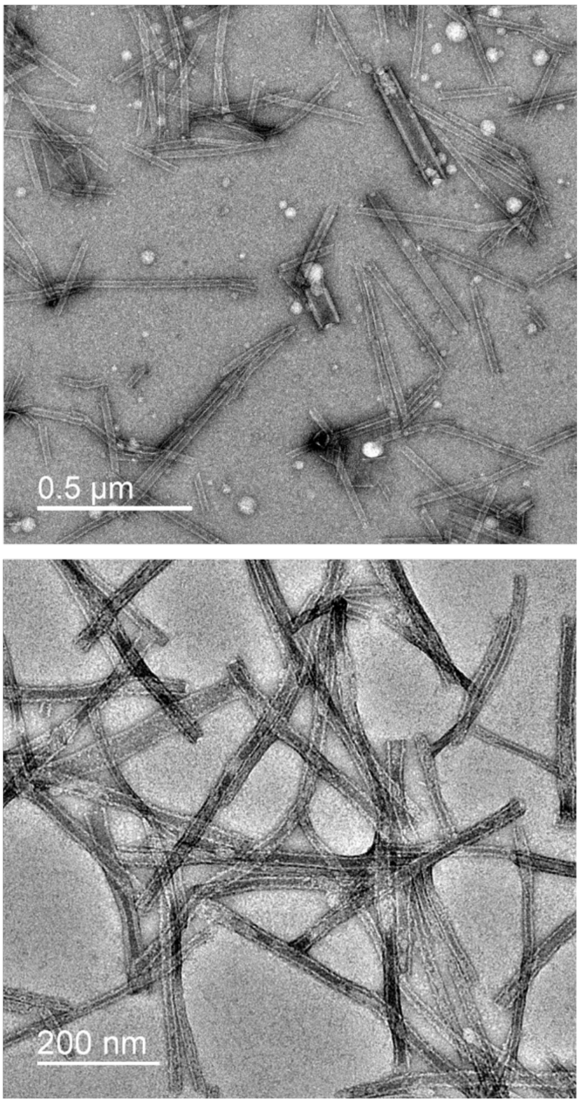

Figure S14. TEM image of $\mathbf{SA_{Ala}}$ (0.2 wt.%, 2.58 mM) in water at (a) pH = 11 after aging for 1 month and (b) pH = 13 after ageing for 1 month.

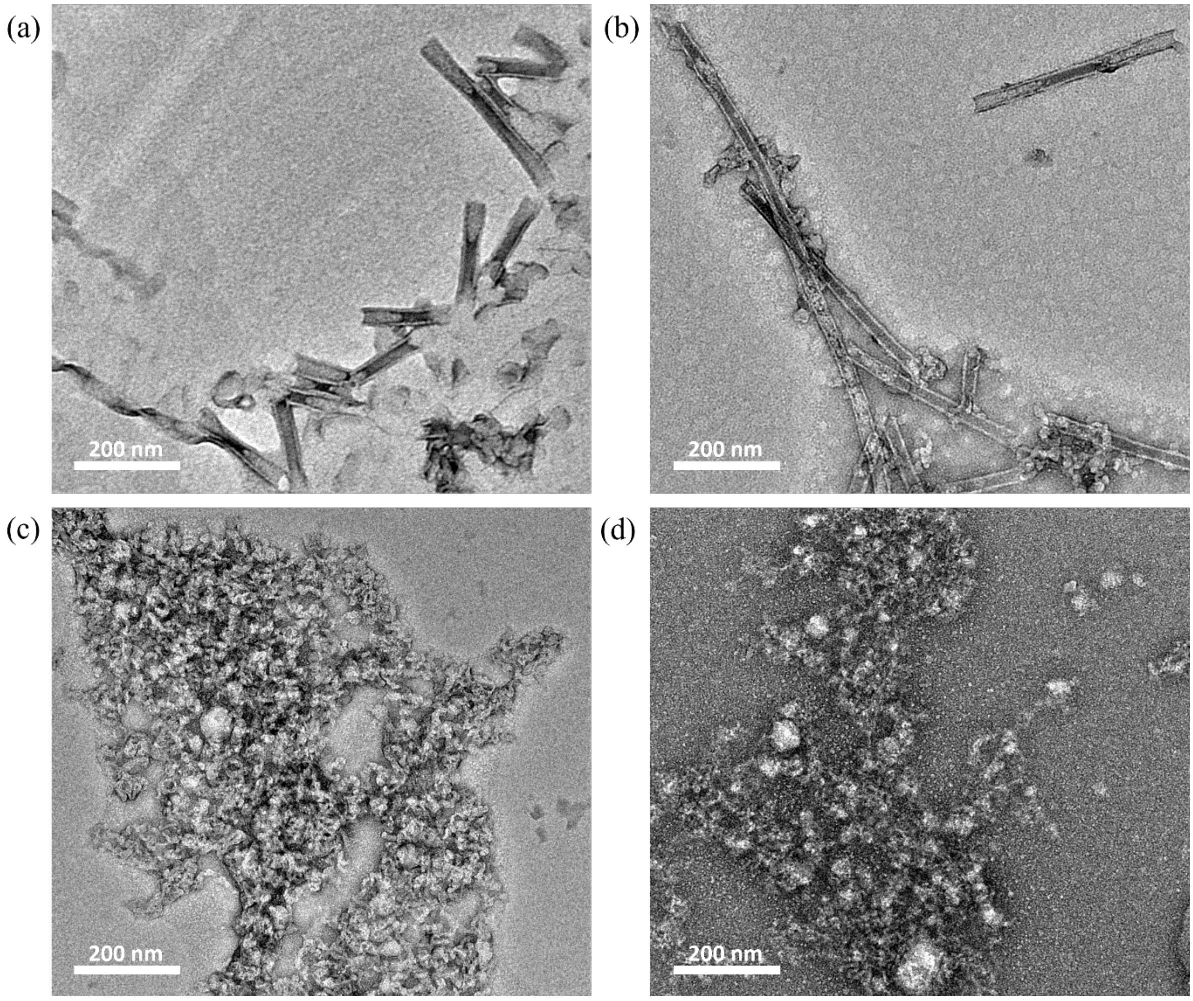

Figure S15. TEM images of $\mathbf{SA_{Val}}$ (0.2 wt.%, 2.41 mM) in water at (a) pH = 7, (b) pH = 9, (c) pH = 11, and (d) pH = 13.

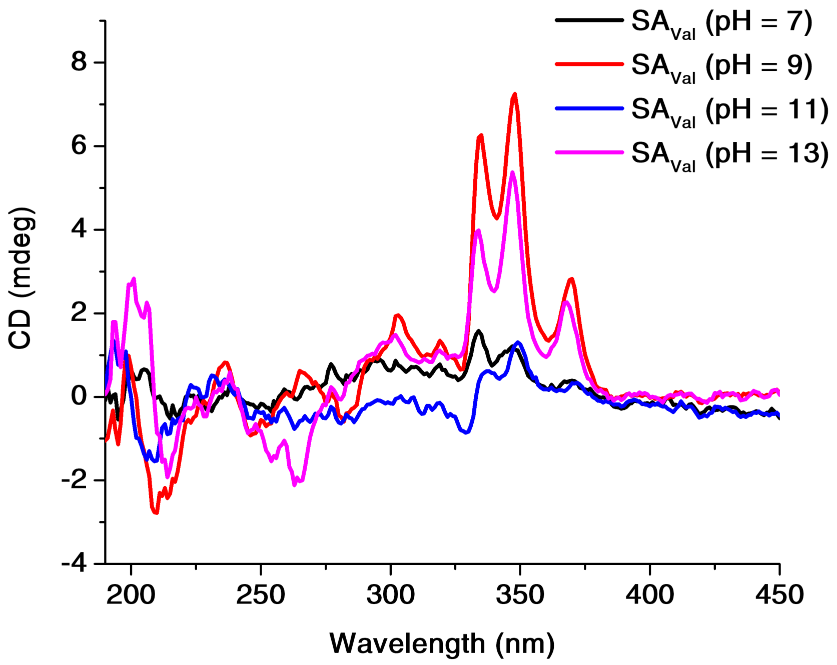

Figure S16. CD spectra of **SA$_{Val}$** (50 μM) in water at pH = 7, 9, 11, and 13.

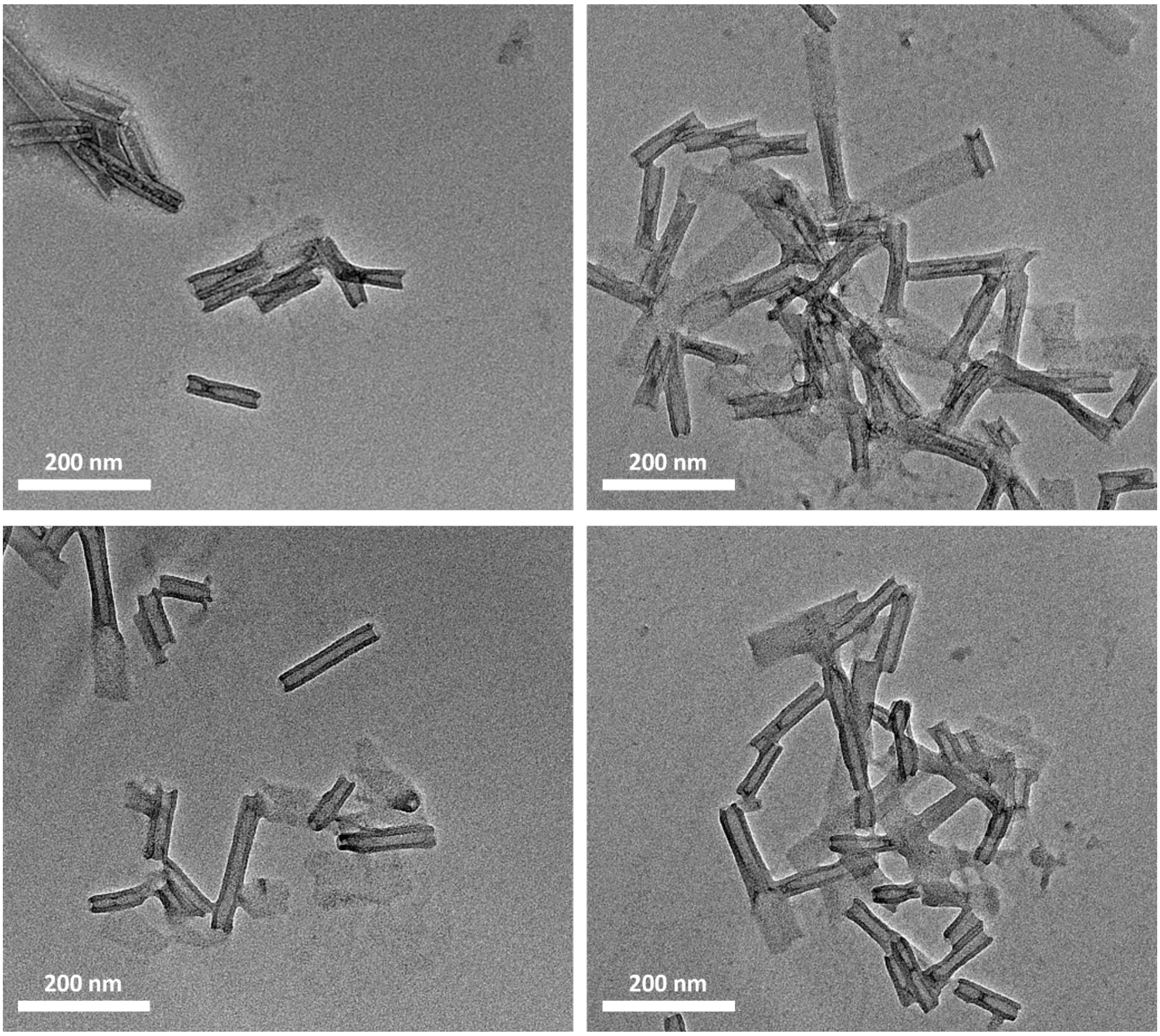

Figure S17. TEM images of short nanotubes of $\mathbf{SA_{Ala}}$ (0.2 wt.%, 2.58 mM, pH = 9) in water, prepared by annealing a high concentration of $\mathbf{SA_{Ala}}$ (65 mM, 5.0 wt.%) for 10 min followed by fast cooling.

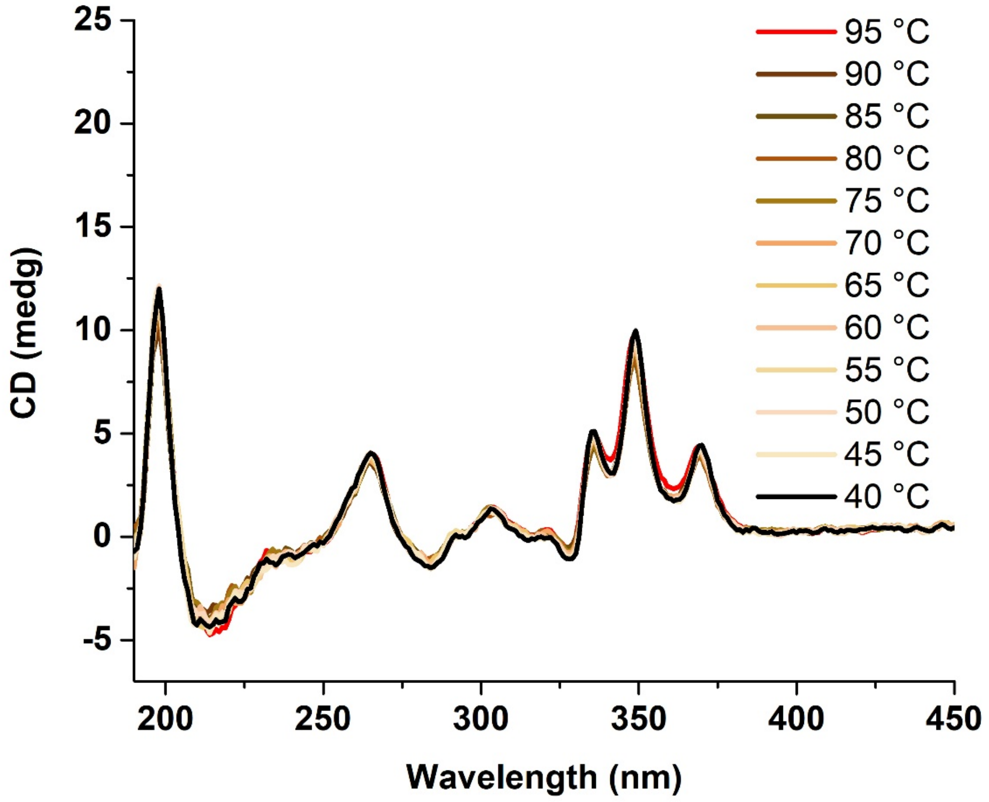

Figure S18. VT-CD spectra of **SA**$_{\mathbf{Ala}}$ in water (50 μM, pH = 9) from 95 °C to 20 °C at a cooling rate of 1 °C/min.

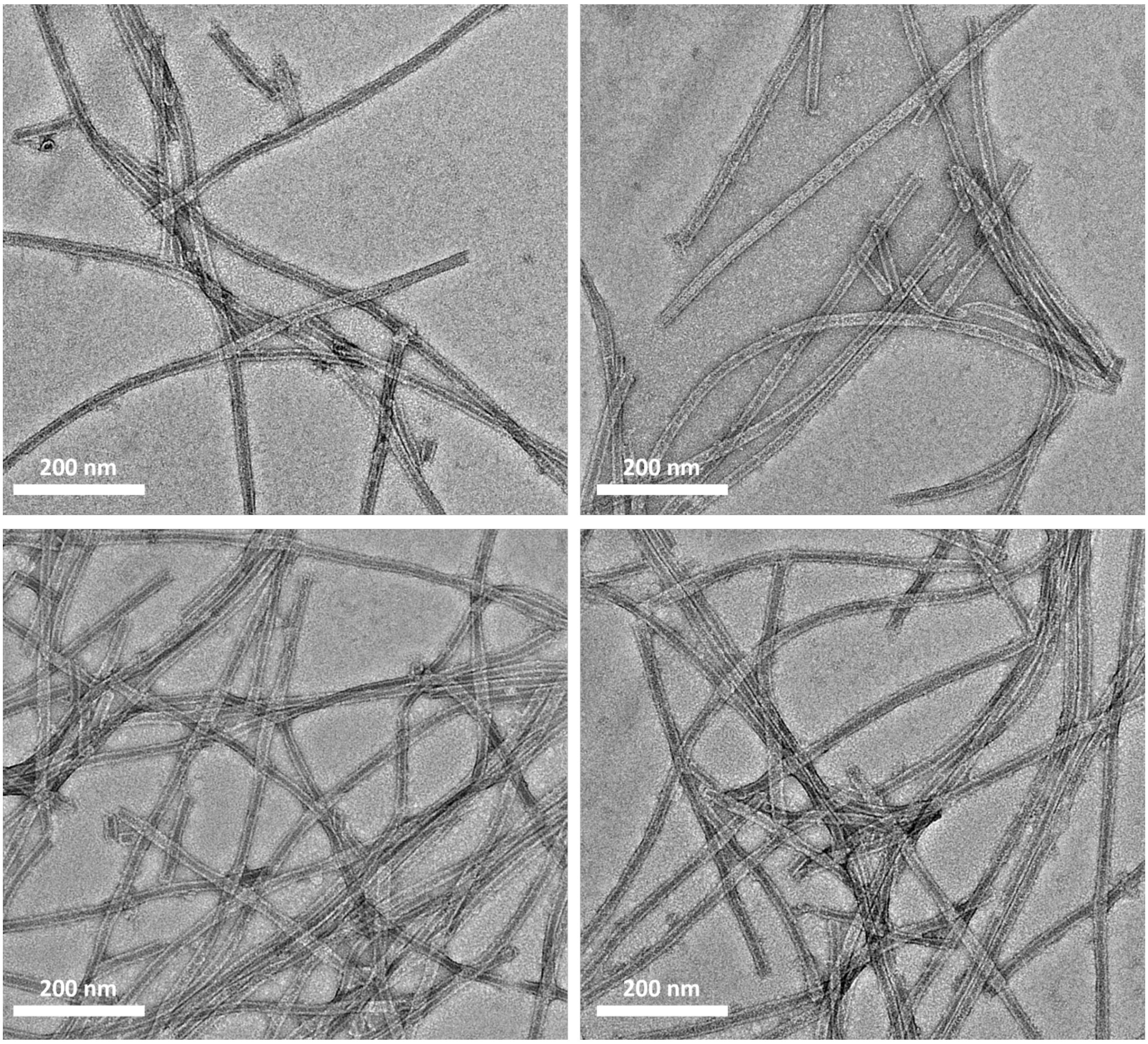

Figure S19. TEM images of $\mathbf{SA_{Ala}}$ (0.2 wt.%, 2.58 mM, pH = 9) in water after 365 nm photoirradiation for 1 h.

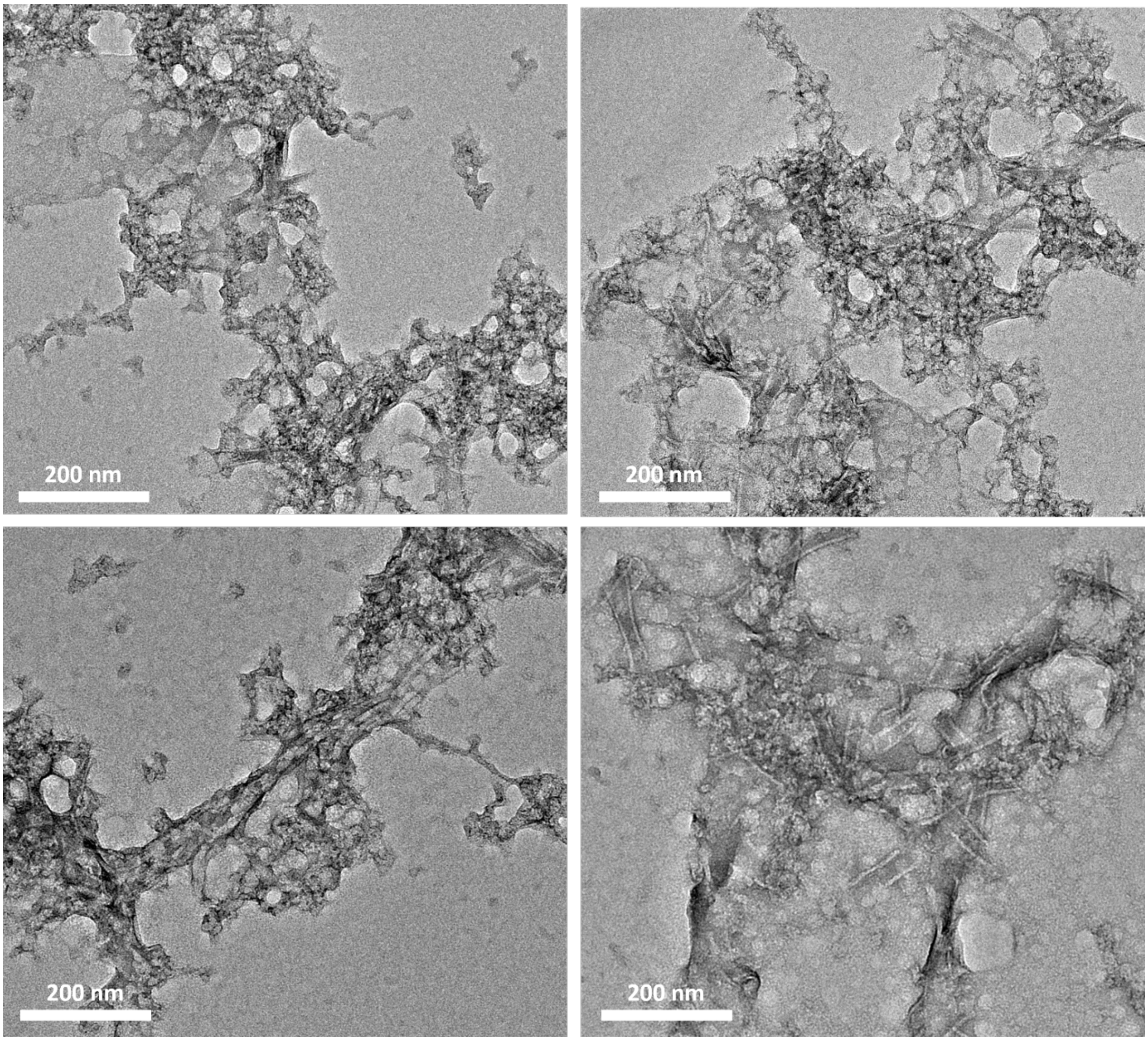

Figure S20. TEM images of **SA$_{Val}$** (0.2 wt.%, 2.41 mM, pH = 9) in water after 365 nm photoirradiation for 1 h.

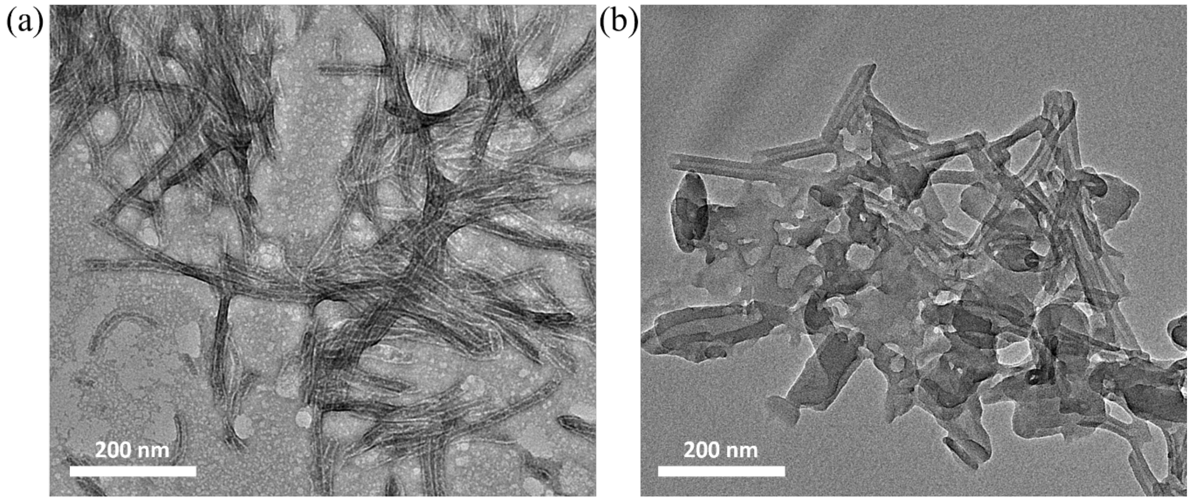

Figure S21. TEM image of **SA**$_{\mathbf{Ala}}$ (0.2 wt.%, 2.58 mM) in DMSO (a) without photoirradiation, (b) after 365 nm photoirradiation for 0.5 h.

(a) (b)

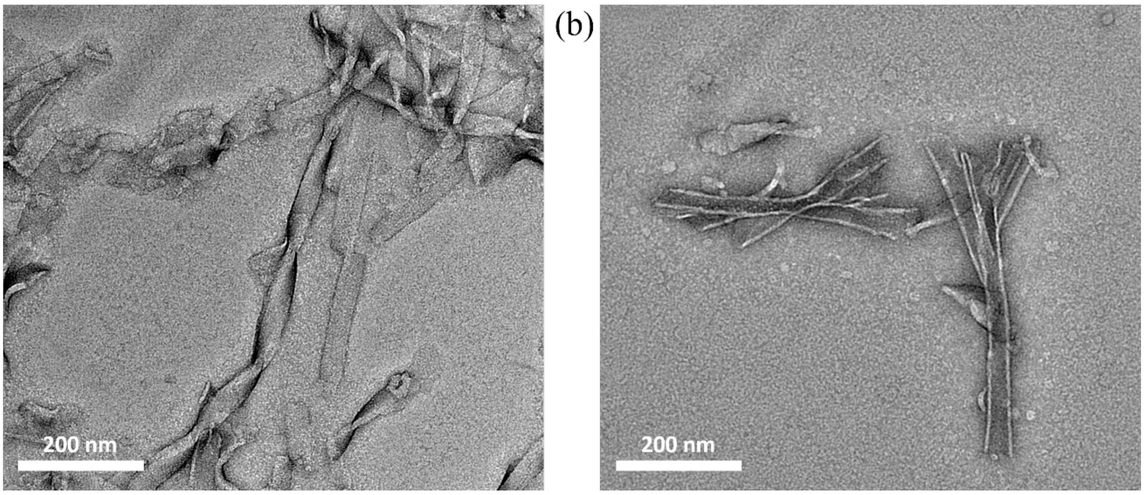

Figure S22. TEM image of $\mathbf{SA_{Val}}$ (0.2 wt.%, 2.41 mM) in DMSO (a) without photoirradiation, (b) after 365 nm photoirradiation for 0.5 h.

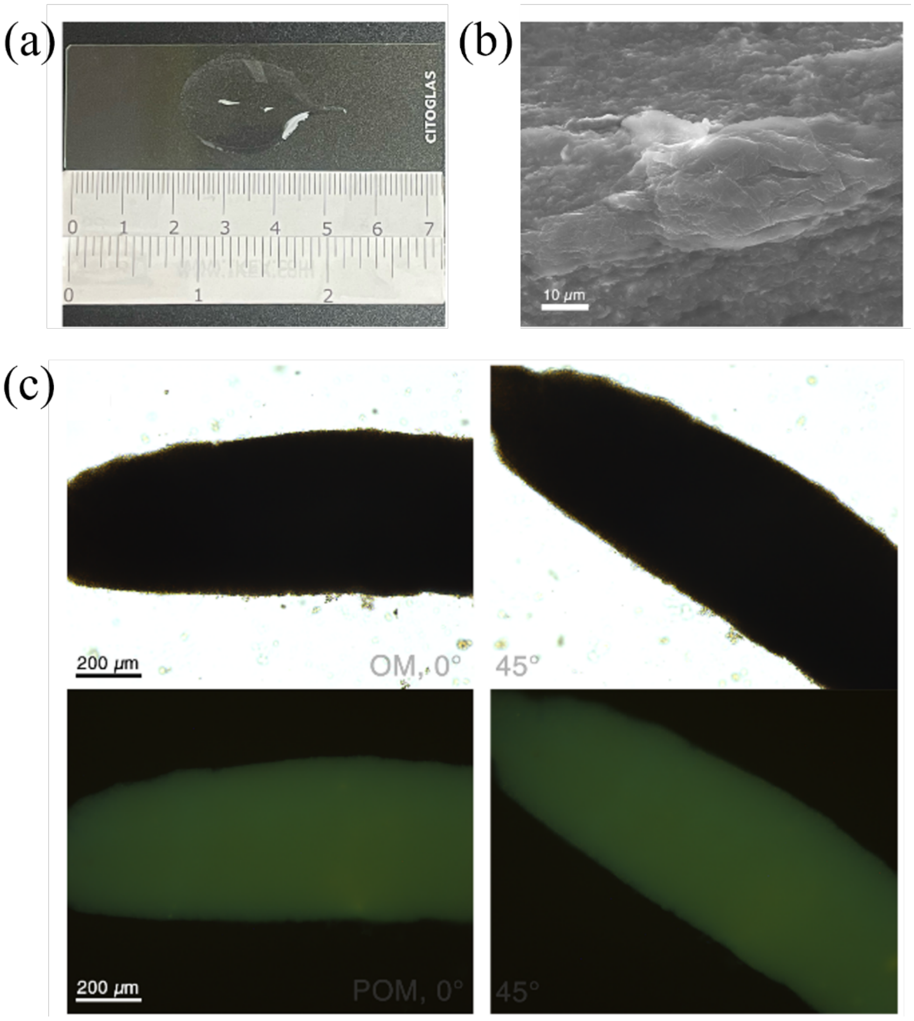

Figure S23. (a) Photograph of aqueous solutions of **SA$_{Ala}$** (5.0 wt.%, 64.5 mM) was ejected into a shallow pool of $CaCl_2$ solution (150 mM). (b) SEM image of **SA$_{Ala}$** (5.0 wt.%, 64.5 mM) prepared from a solution of $CaCl_2$ (150 mM). (c) Optical microscopic images of a macroscopic string composed of **SA$_{Ala}$** prepared from a solution of $CaCl_2$ (150 mM) without (top) and with (bottom) polarizers at 0° and 45°.

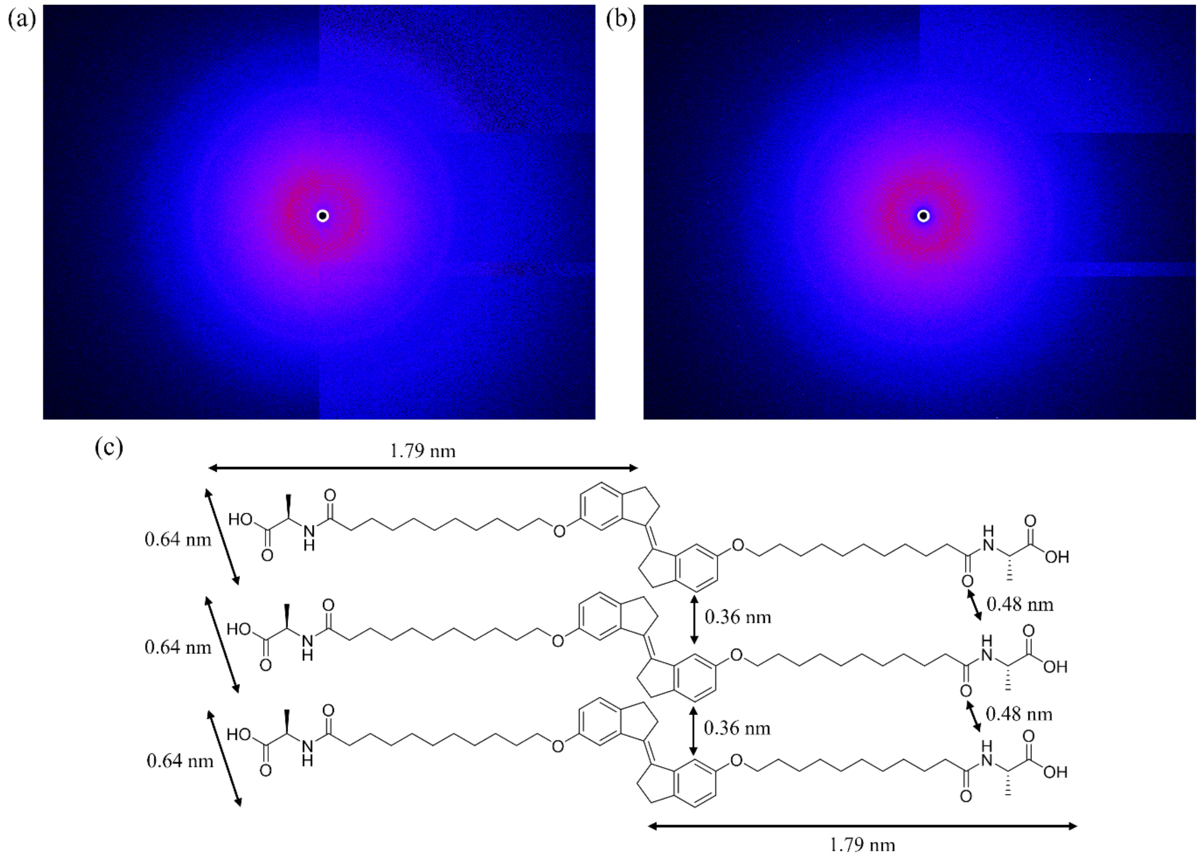

Figure S24. 2D-WAXD image of a macroscopic soft scaffold of **SA$_{Ala}$** (5.0 wt.%, 64.5 mM) (a) before and (b) after irradiation with 365 nm UV light. (c) Schematic illustration of assembled **SA$_{Ala}$** leads to diffraction in WAXD.

(a)

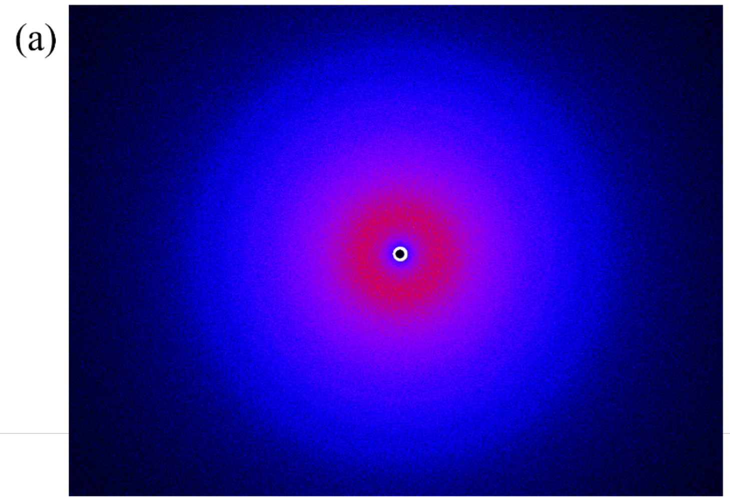

(b)

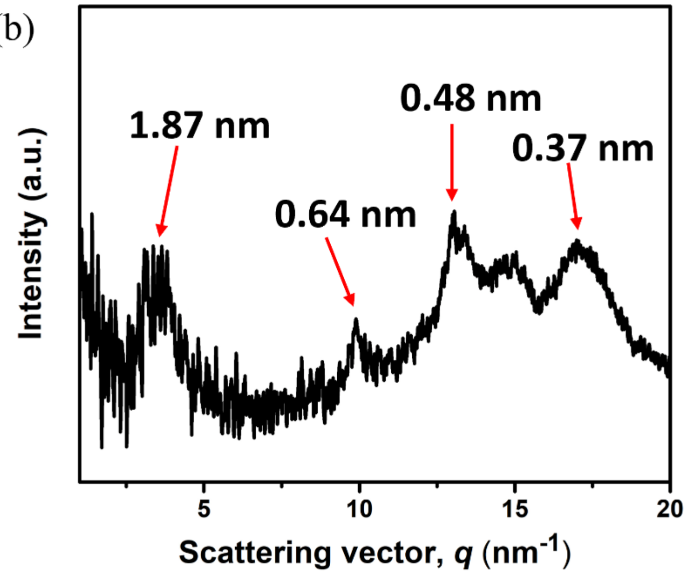

Figure S25. (a) 1D-WAXD pattern of a macroscopic soft scaffold of **SA$_{Ala}$** at pH = 11. (b) 2D-WAXD image of a macroscopic soft scaffold of **SA$_{Ala}$** at pH = 11

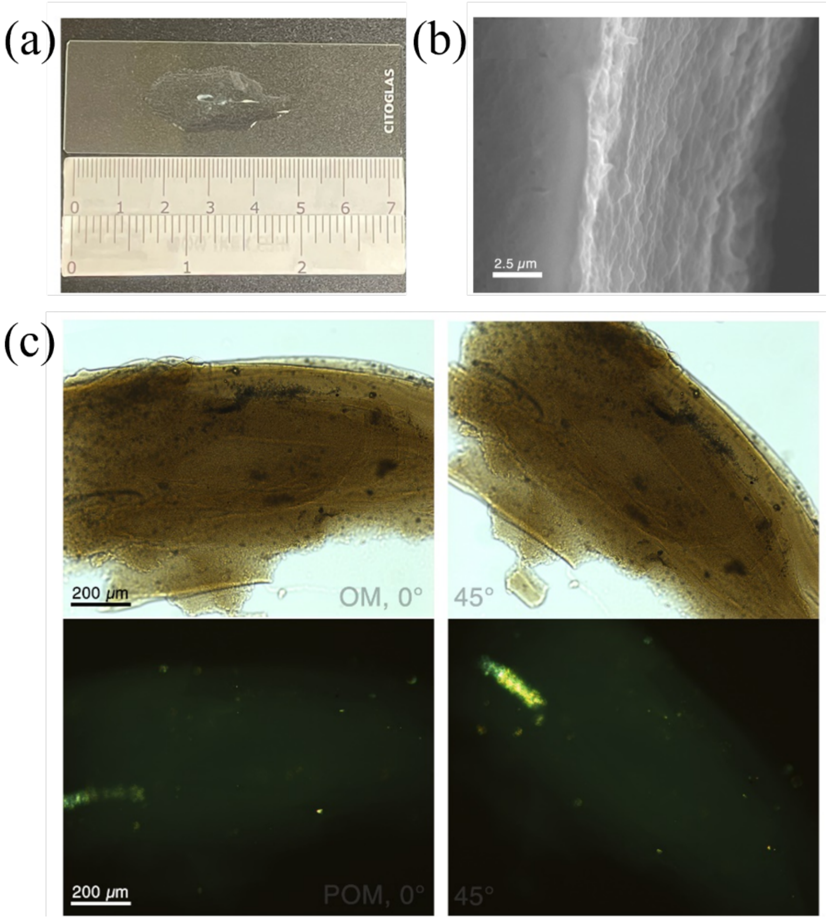

Figure S26. (a) Photograph of aqueous solutions of **SA$_{Val}$** (5.0 wt.%, 60.2 mM) was ejected into a shallow pool of $CaCl_2$ solution (150 mM). (b) SEM image of **SA$_{Val}$** (5.0 wt.%, 60.2 mM) prepared from a solution of $CaCl_2$ (150 mM). (c) Optical microscopic images of a macroscopic string composed of **SA$_{Val}$** prepared from a solution of $CaCl_2$ (150 mM) without (top) and with (bottom) crossed polarizers at 0°, 45°.

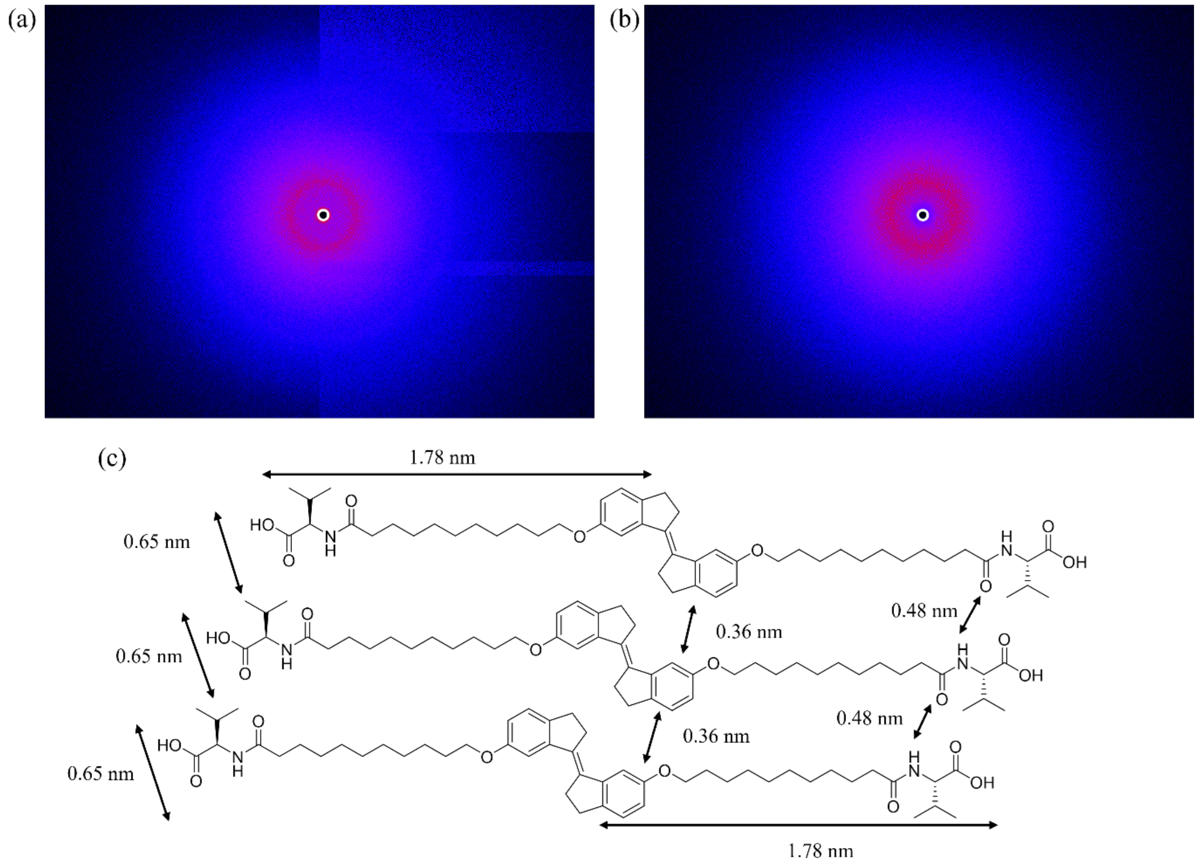

Figure S27. 2D-WAXD image of a macroscopic soft scaffold of **SA$_{\mathbf{Val}}$** (5.0 wt.%, 60.2 mM) (a) before and (b) after irradiation with 365 nm UV light. (c) Schematic illustration of assembled **SA$_{\mathbf{Val}}$** leads to diffraction in WAXD.

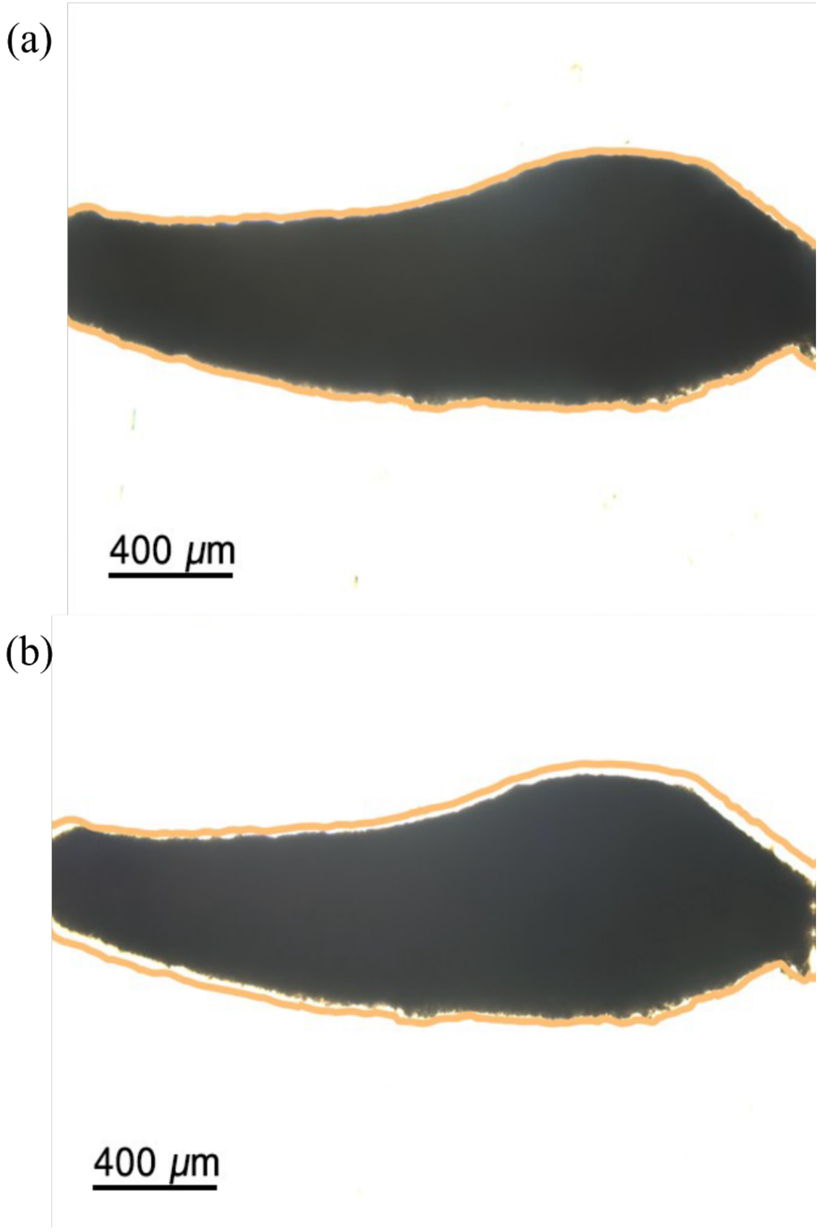

Figure S28. Snapshots of a macroscopic soft scaffold of **SA$_{Ala}$** (5.0 wt.%, 64.5 mM) (a) before and (b) after irradiation with 365 nm UV light.

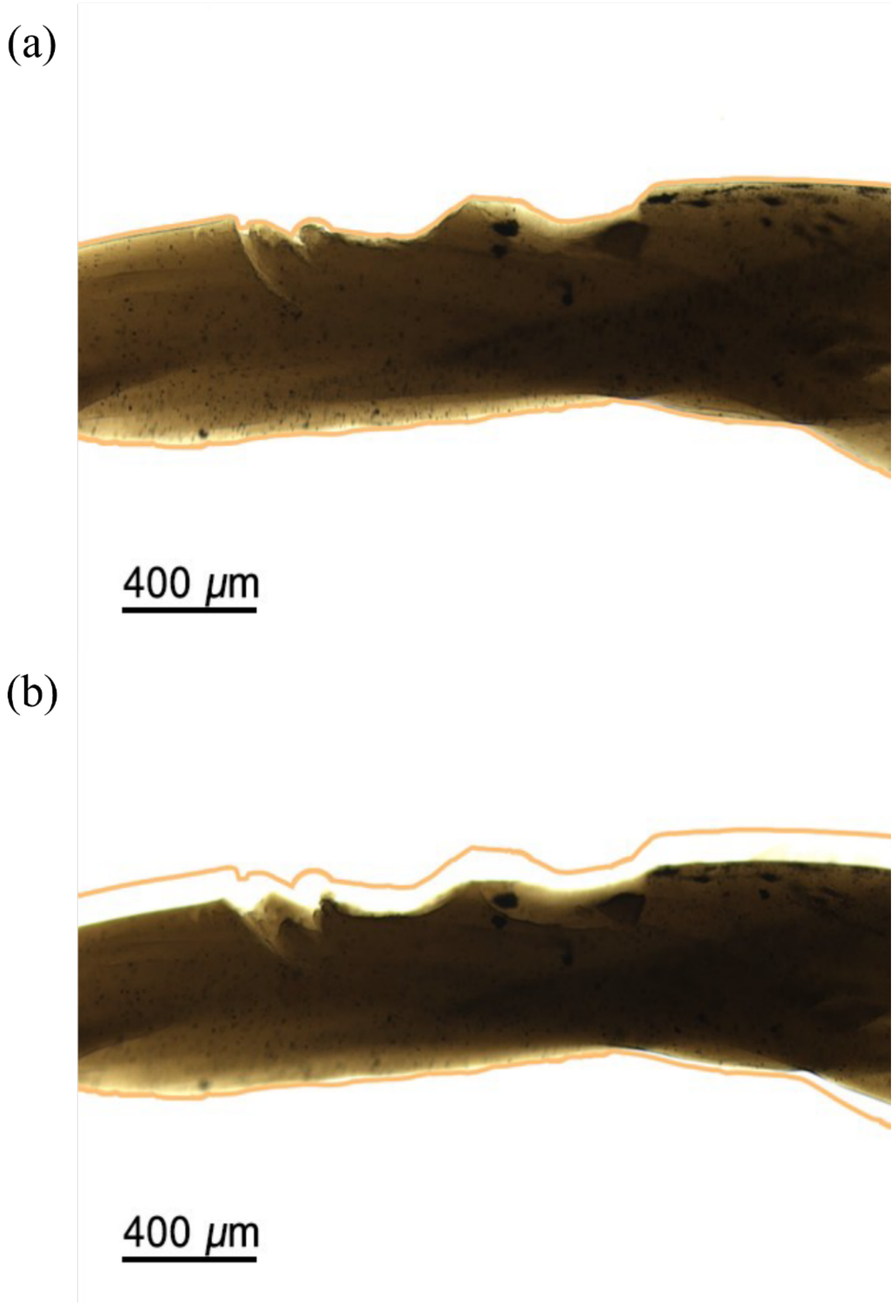

Figure S29. Snapshots of a macroscopic soft scaffold of **SA$_{\mathbf{Val}}$** (5.0 wt.%, 60.2 mM) (a) before and (b) after irradiation with 365 nm UV light.

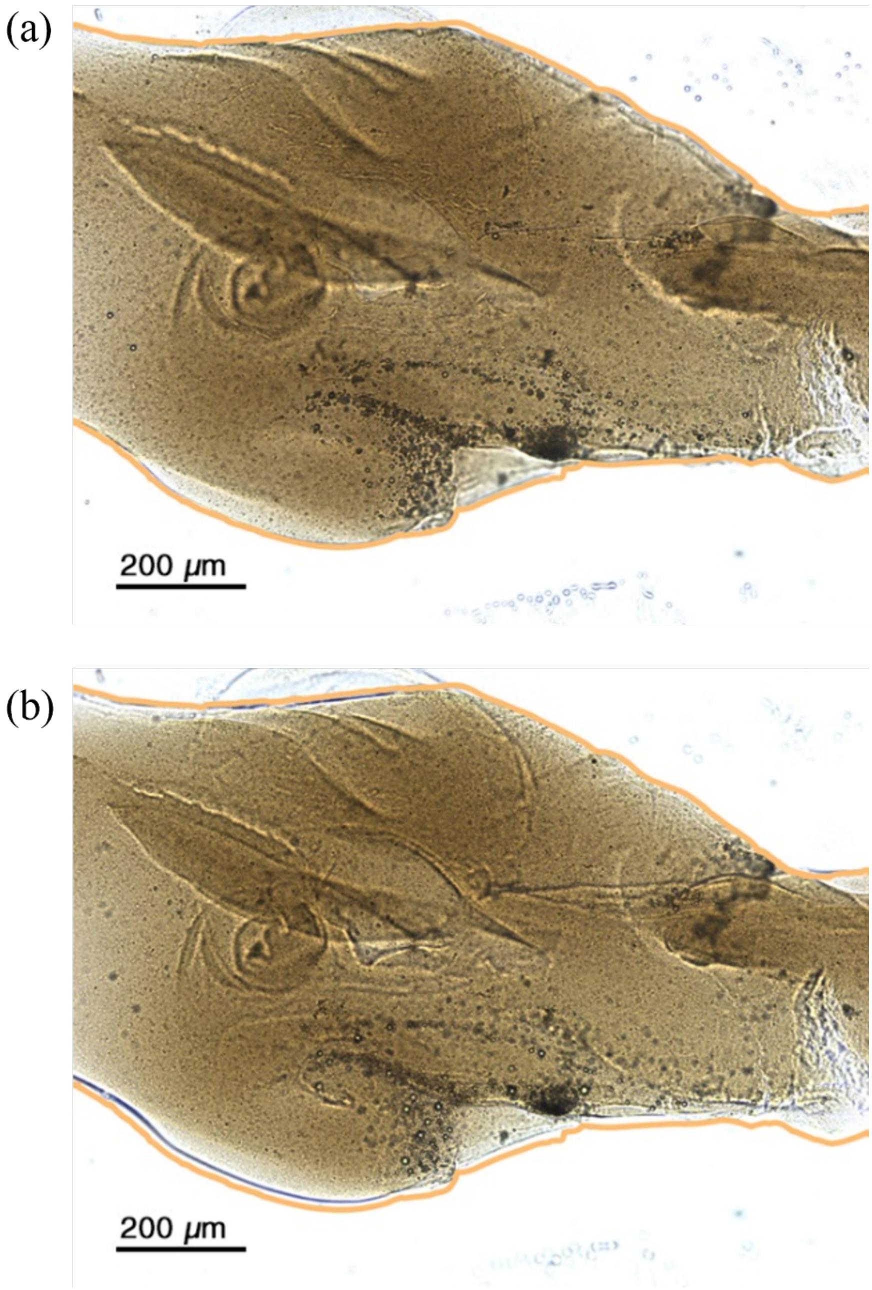

Figure S30. Snapshots of a macroscopic soft scaffold of $\mathbf{SA_{Ala}}$: $\mathbf{SA_{Val}}$ (ratio of 9:1) (a) before and (b) after irradiation with 365 nm UV light.

(a)

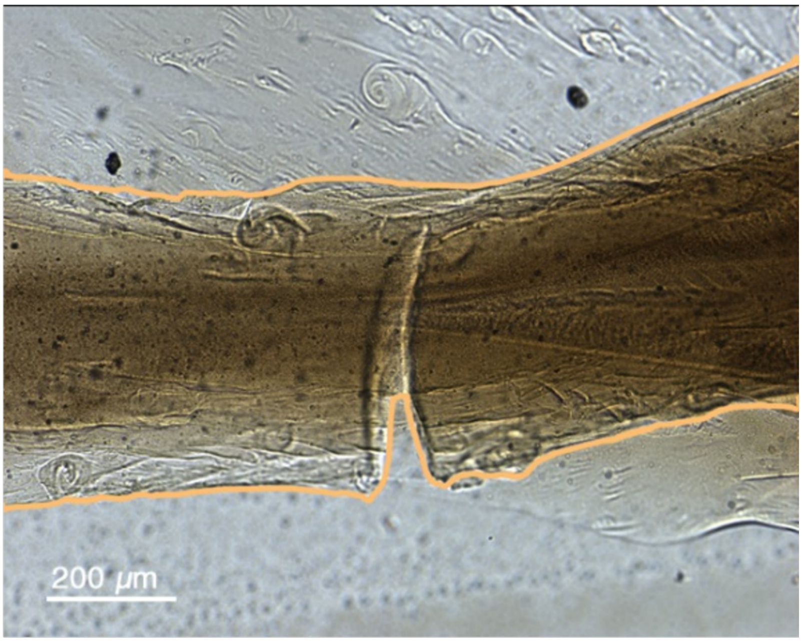

(b)

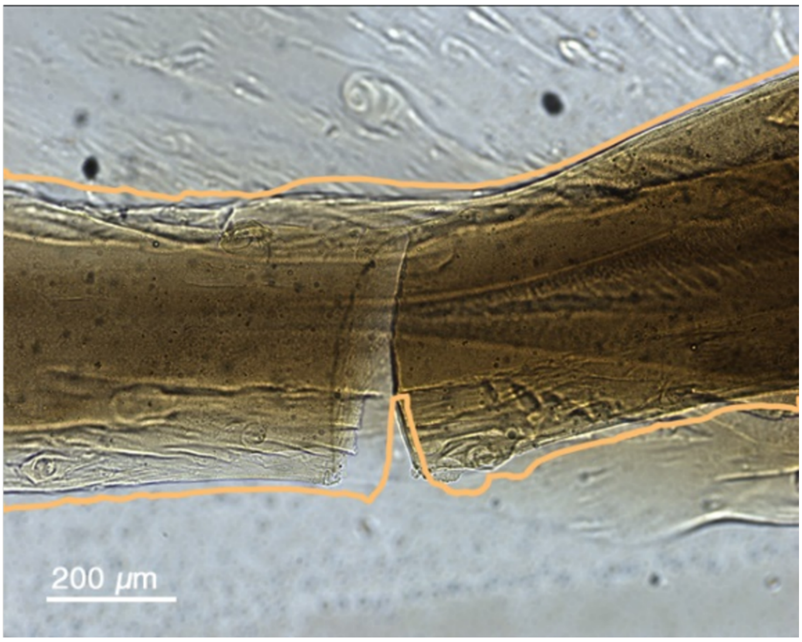

Figure S31. Snapshots of a macroscopic soft scaffold of $\mathbf{SA_{Ala}}$: $\mathbf{SA_{Val}}$ (ratio of 1:1) (a) before and (b) after irradiation with 365 nm UV light.

(a)

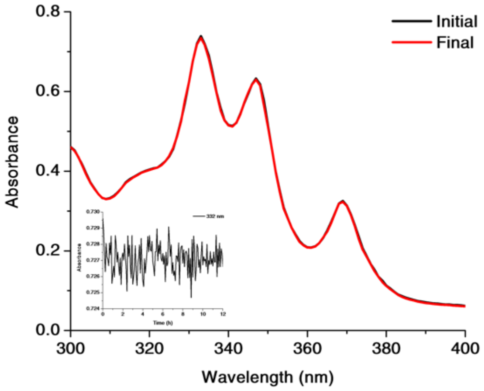

(b)

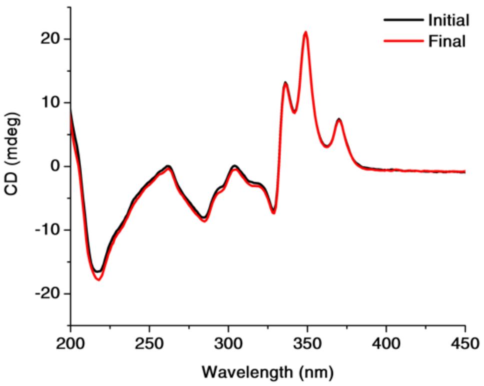

(c)

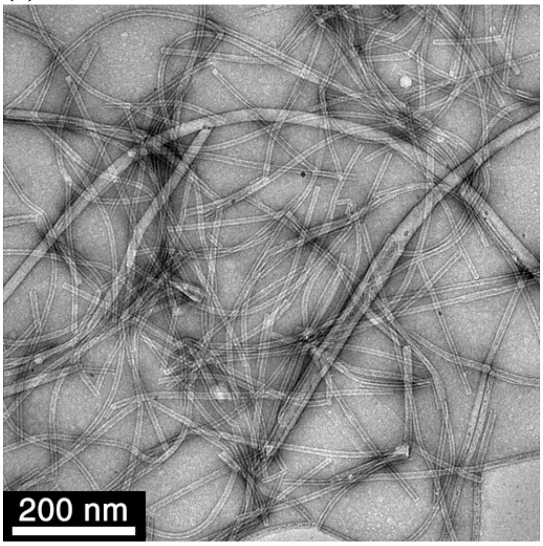

Figure S32. Stability test of nanotubes of $\mathbf{SA_{Ala}}$ prepared by annealing a 0.2 wt.% solution at 85 °C for 70 min followed by 1 °C/min to 20 °C. (a) UV-vis absorption spectra of $\mathbf{SA_{Ala}}$ (50 μM, pH = 7) at 37 °C for 12 h (inset: time-course of the stability experiment of $\mathbf{SA_{Ala}}$ monitored at λ_{max} 332 nm) and (b) corresponding CD spectra. (c) TEM images of aqueous solutions of $\mathbf{SA_{Ala}}$ (50 μM, pH = 7) after stability test at 37 °C for 12 h.

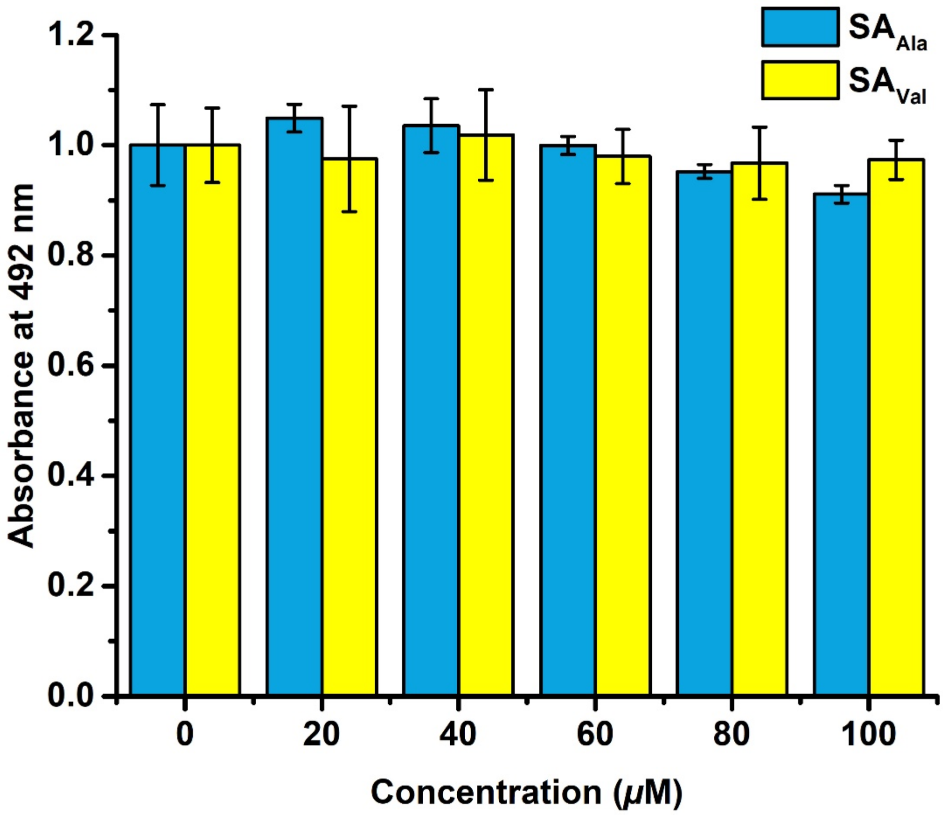

Figure S33. Bar chart of MTS assay of nanotubes of **SA_{Ala}** and **SA_{Val}**.

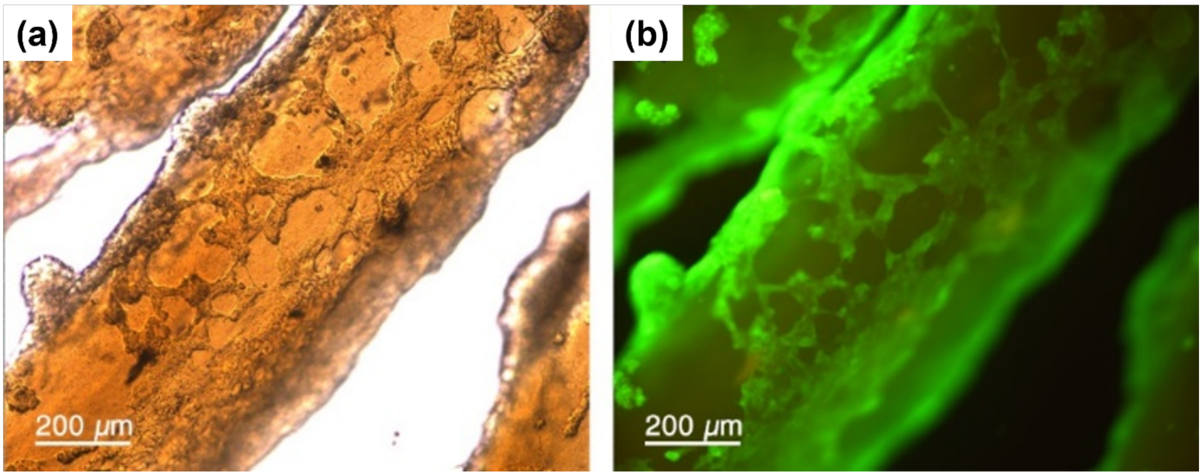

Figure S34. (a) OM and (b) fluorescent images of HeLa cell attached on a macroscopic string of $\mathbf{SA_{Ala}}$ (5 wt.%, 65 mM) at 10x magnification.

4. Analytical Data

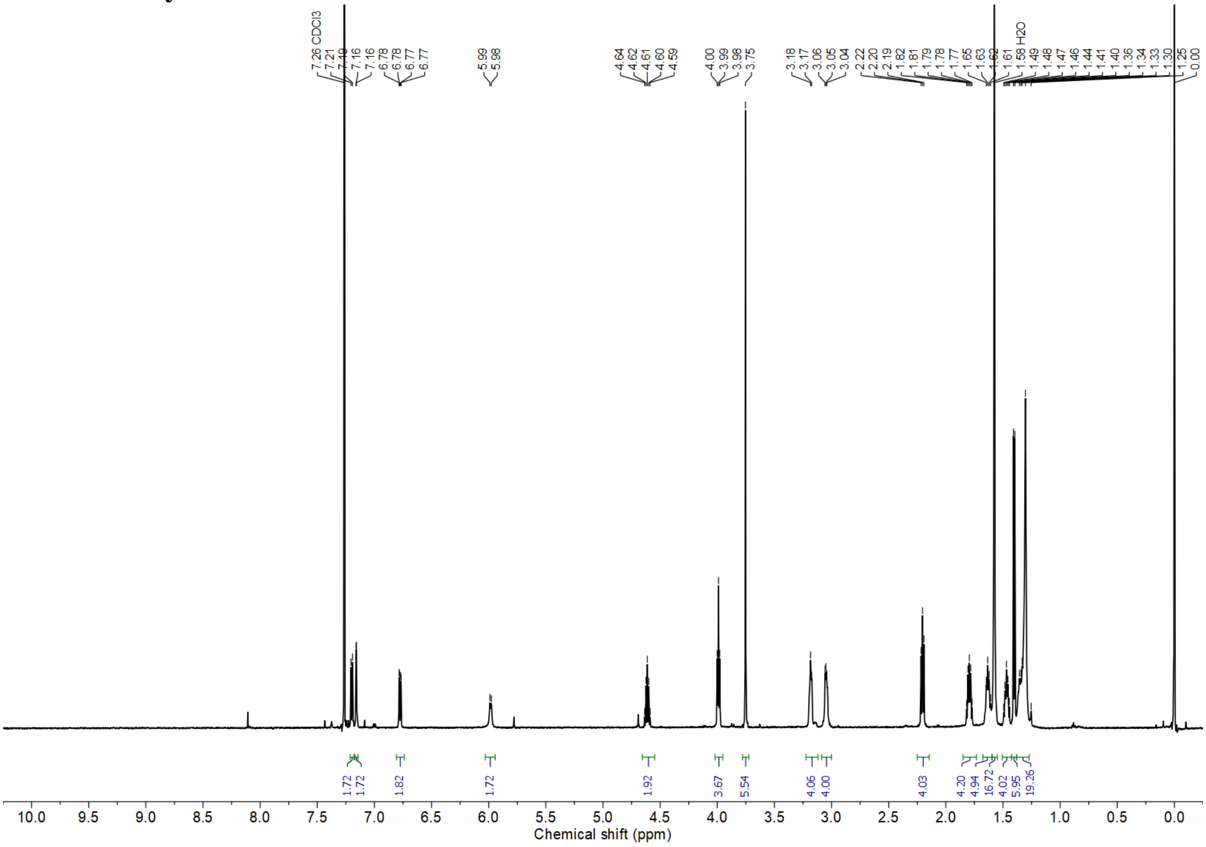

Figure S35. ^{1}H NMR spectrum (600 MHz) of compound **1** in $CDCl_3$ at 25 °C.

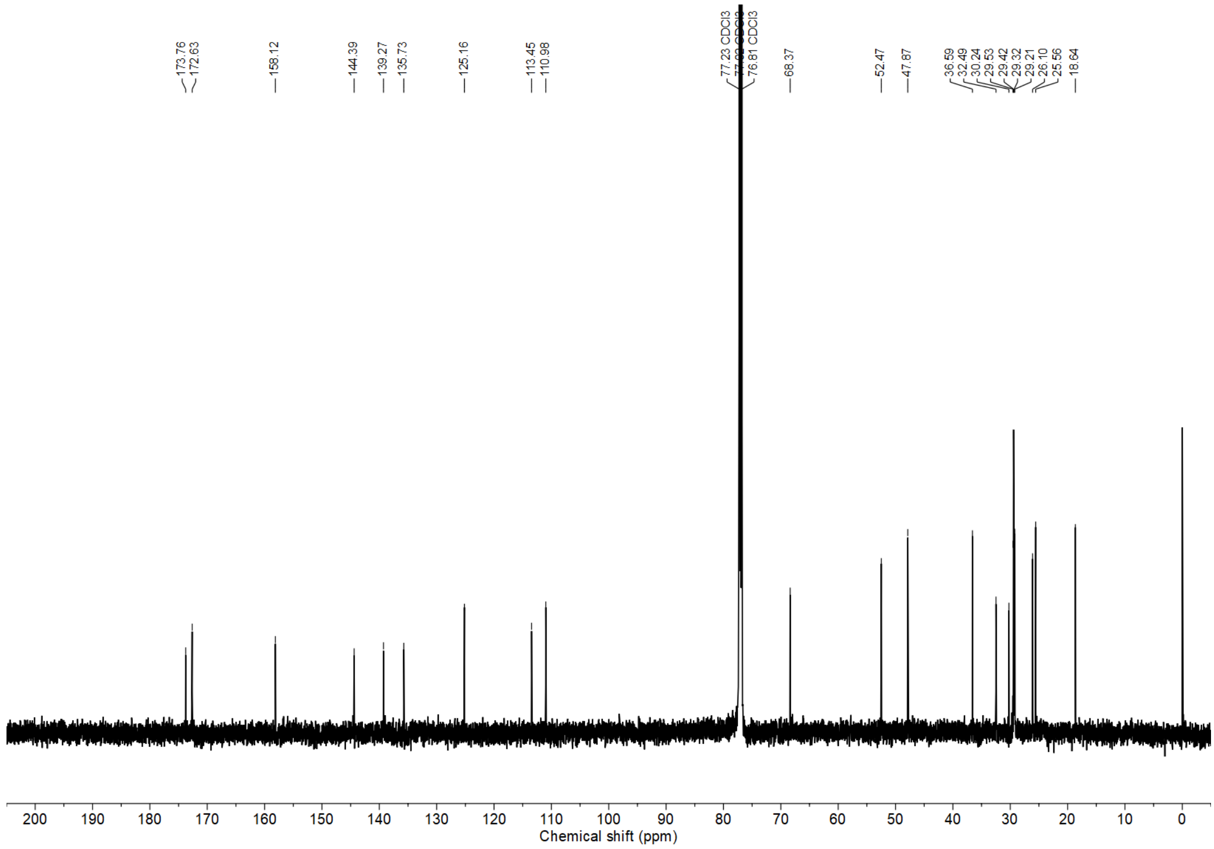

Figure S36. ^{13}C NMR spectrum (151 MHz) of compound **1** in $CDCl_3$ at 25 °C.

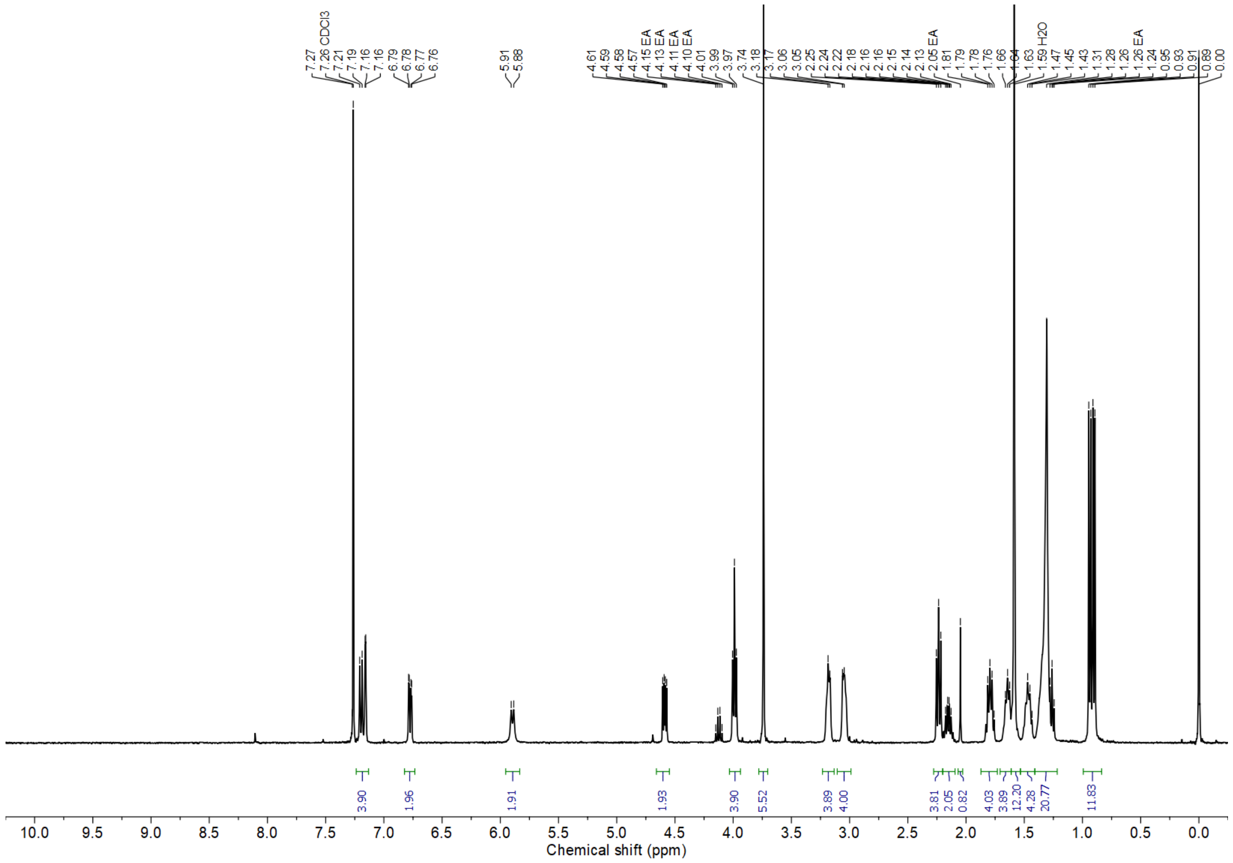

Figure S37. ^{1}H NMR spectrum (400 MHz) of compound **2** in $CDCl_3$ at 25 °C.

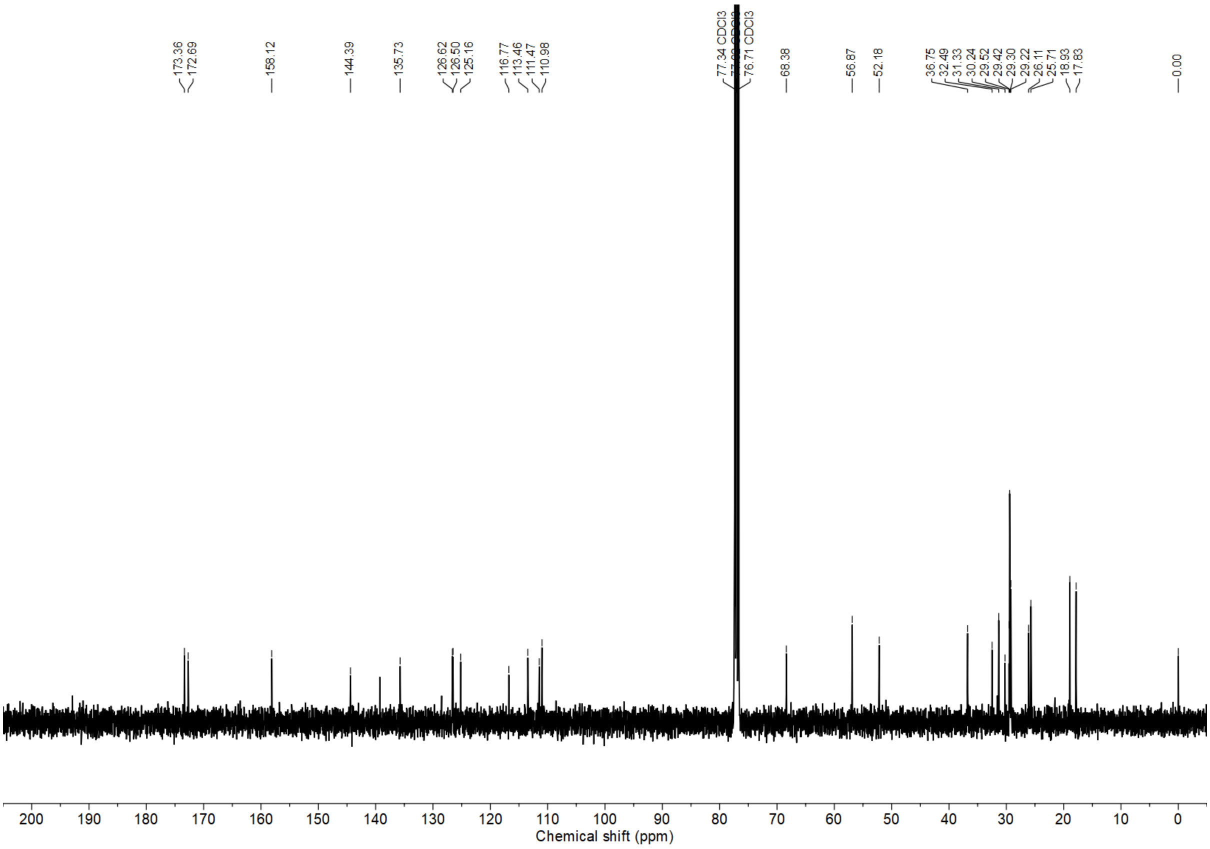

Figure S38. ^{13}C NMR spectrum (101 MHz) of compound **2** in $CDCl_3$ at 25 °C.

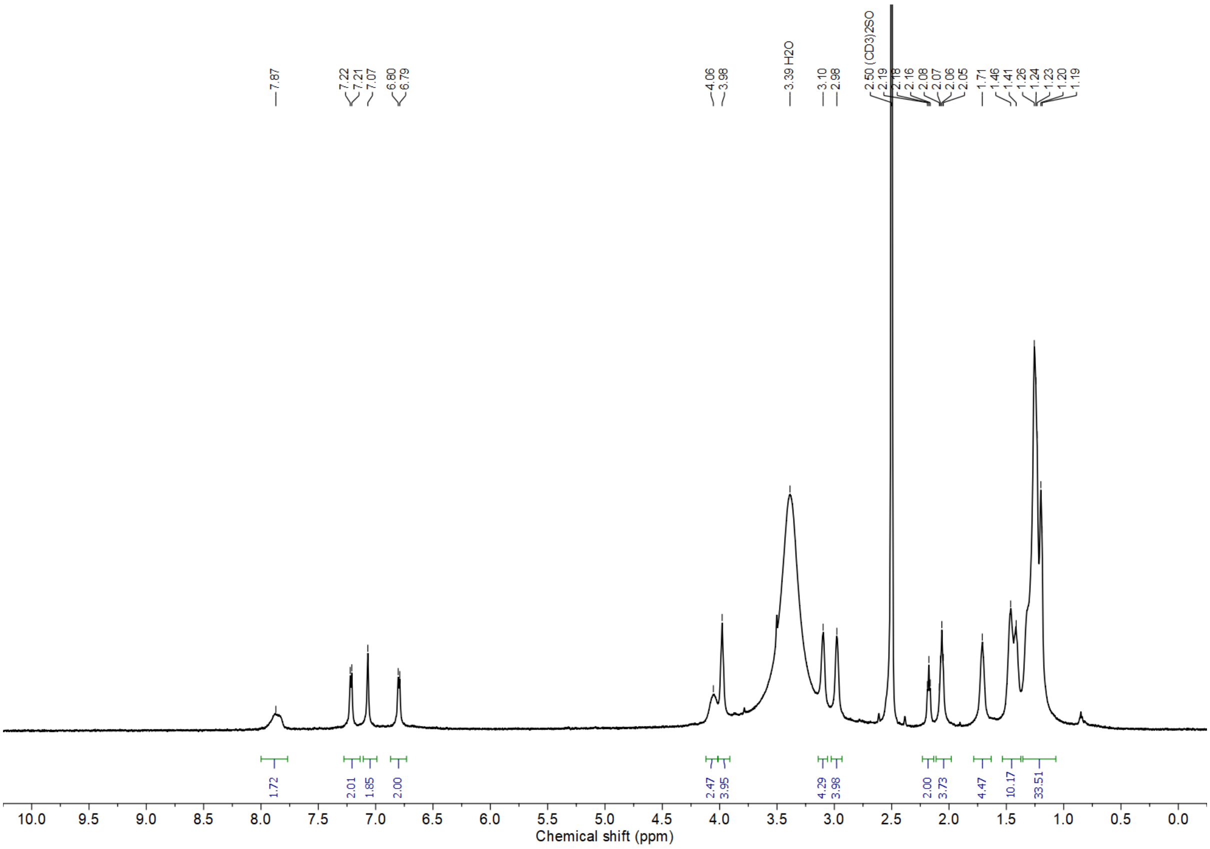

Figure S39. ^{1}H NMR spectrum (600 MHz) of compound **SA_{Ala}** in $(CD_3)_2SO$ at 25 °C.

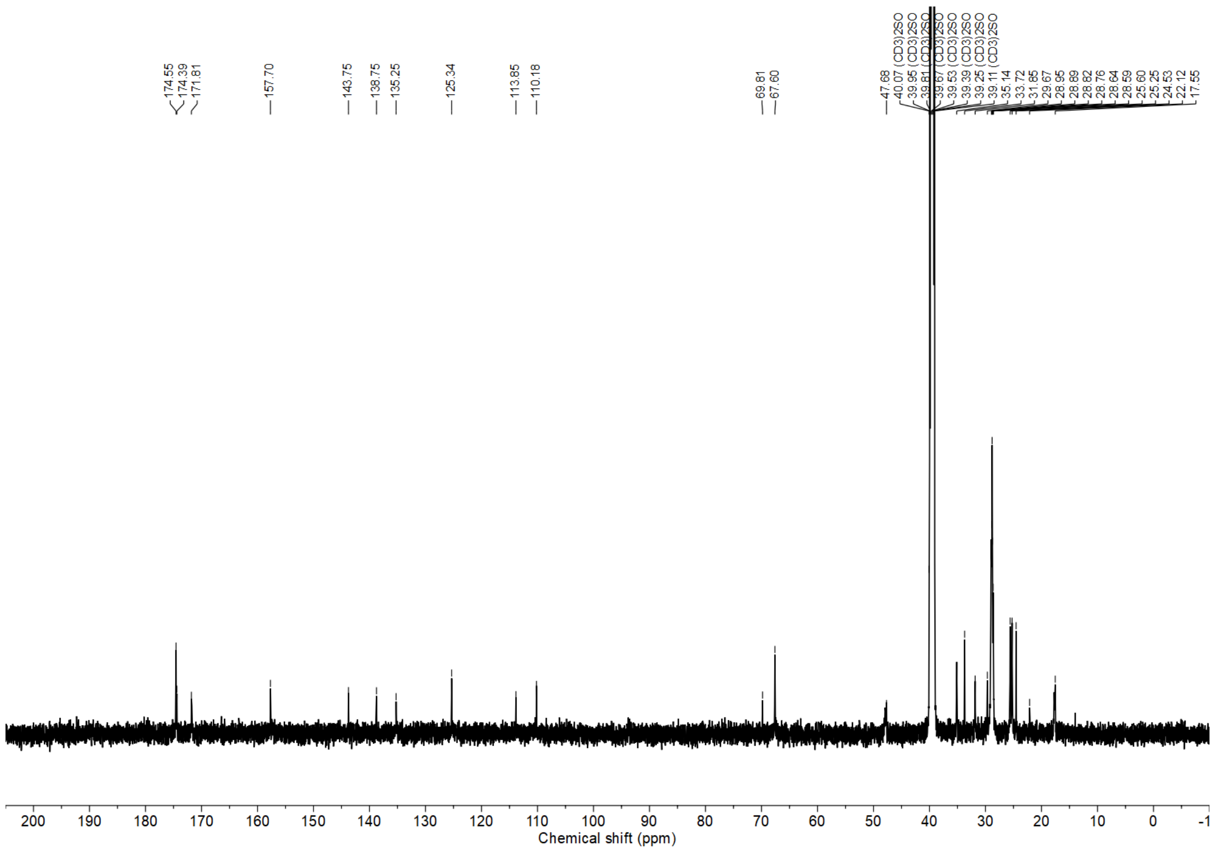

Figure S40. ^{13}C NMR spectrum (151 MHz) of $\mathbf{SA_{Ala}}$ in $(CD_3)_2SO$ at 25 °C.

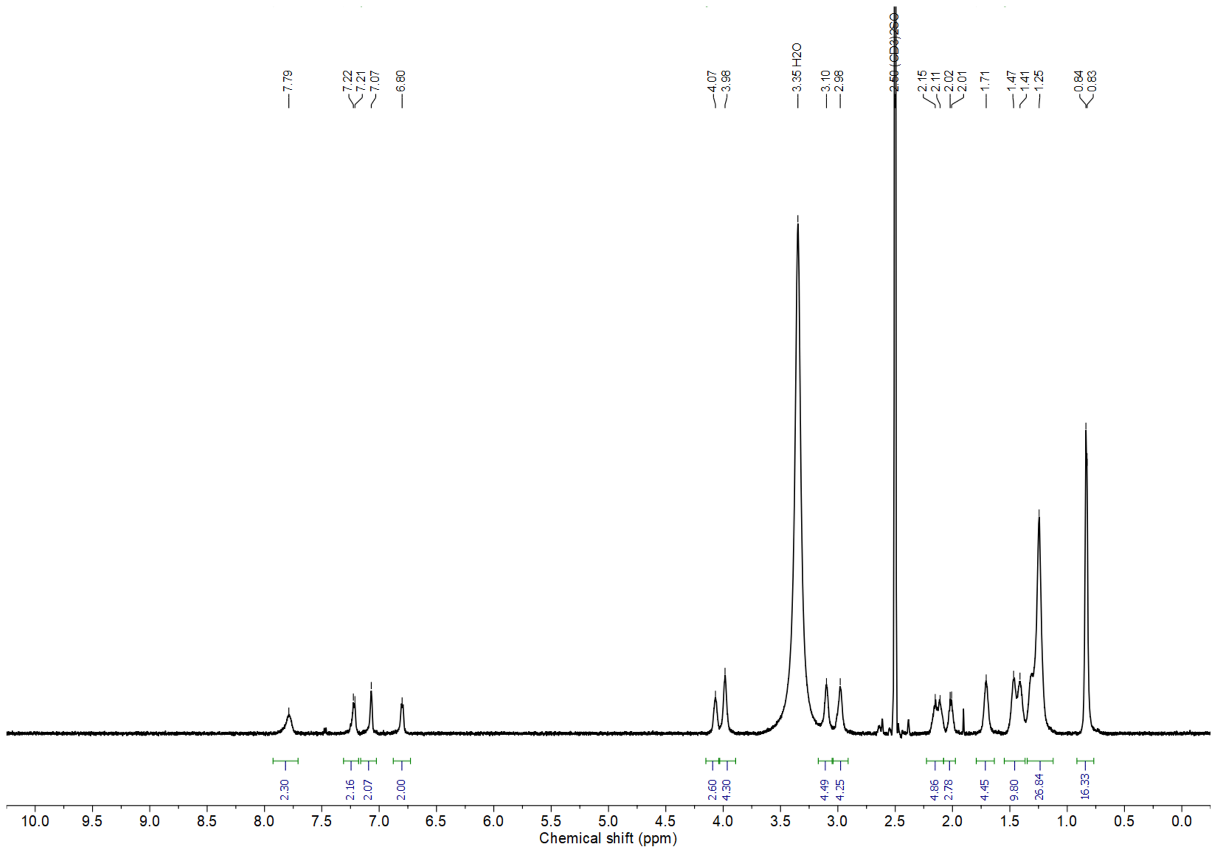

Figure S41. ^{1}H NMR spectrum (600 MHz) of compound **SA$_{Val}$** in $(CD_3)_2SO$ at 25 °C.

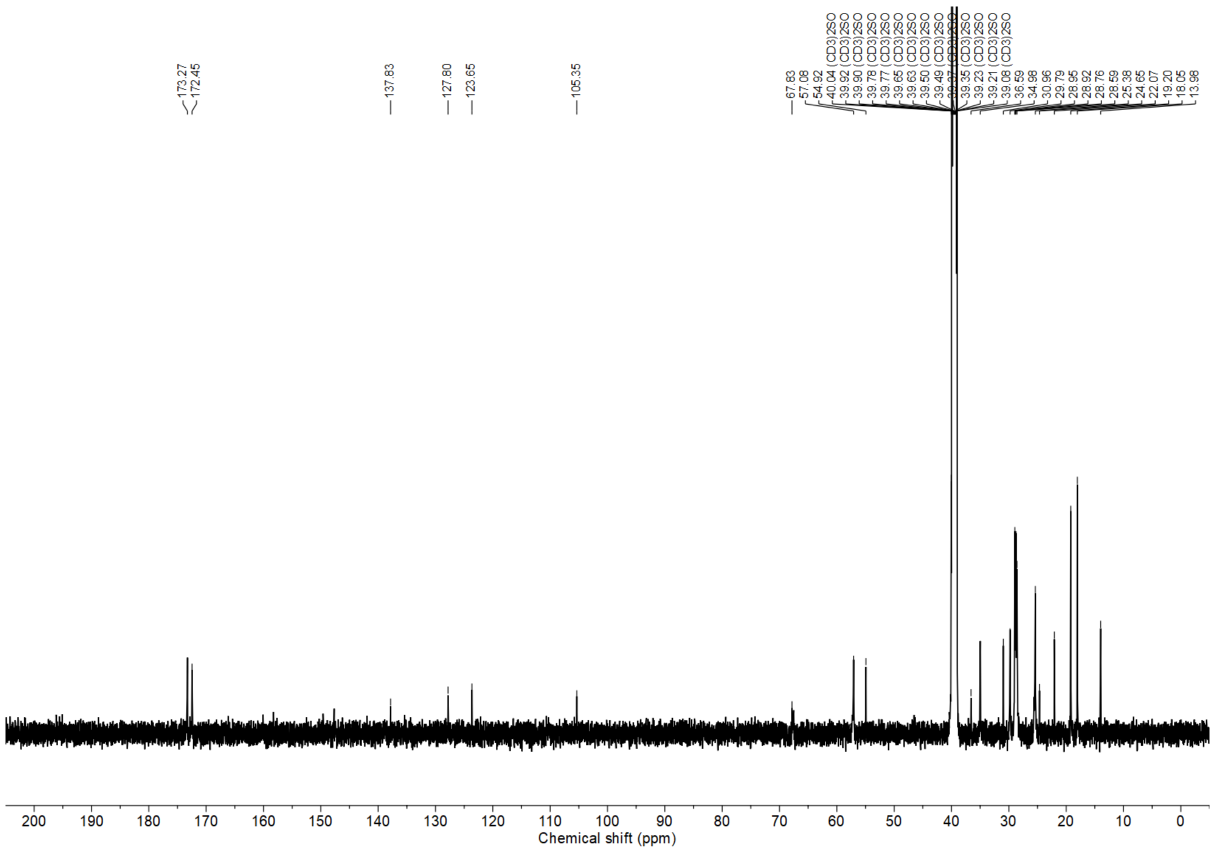

Figure S42. ^{13}C NMR spectrum (151 MHz) of **SA$_{Val}$** in $(CD_3)_2SO$ at 25 °C.
